# Reassessing the causal role of obesity in breast cancer susceptibility – a comprehensive multivariable Mendelian randomization investigating the distribution and timing of exposure

**DOI:** 10.1093/ije/dyac143

**Published:** 2022-07-15

**Authors:** Yu Hao, Jinyu Xiao, Yu Liang, Xueyao Wu, Chenghan Xiao, Li Zhang, Nan Wang, Xunying Zhao, Haoyu Zhang, Stephen Burgess, Peter Kraft, Jiayuan Li, Xia Jiang

**Affiliations:** 1 Department of Epidemiology and Biostatistics, West China School of Public Health and West China Fourth Hospital, Sichuan University, Chengdu, Sichuan, China; 2 Department of Maternal, Child and Adolescent Health, West China School of Public Health and West China Fourth Hospital, Sichuan University, Chengdu, Sichuan, China; 3 Division of Cancer Epidemiology and Genetics, National Cancer Institute, National Institutes of Health, Department of Health and Human Services, Bethesda, MD, USA; 4 Department of Biostatistics, Johns Hopkins Bloomberg School of Public Health, Baltimore, MD, USA; 5 MRC Biostatistics Unit, University of Cambridge, Cambridge, UK; 6 Department of Epidemiology, Harvard T.H. Chan School of Public Health, Boston, MA, USA; 7 Department of Nutrition and Food Hygiene, West China School of Public Health and West China Fourth Hospital, Sichuan University, Chengdu, Sichuan, China

## Abstract

**Background:**

Previous Mendelian randomization (MR) studies on obesity and breast cancer (BC) risk adopted a small number of instrumental variables and mainly focused on crude total causal effects. We aim to investigate the independent causal effect of obesity-related exposures on breast cancer susceptibility, taking into consideration the distribution of fat, covering both early and late life.

**Methods:**

Using an enlarged set of female-specific genetic variants associated with adult general (body mass index, BMI) and abdominal obesity (waist-to-hip ratio with and without adjusted for BMI, WHR and WHR_adj_BMI) as well as using sex-combined genetic variants of childhood obesity (childhood BMI), we performed a two-sample univariable MR (UVMR) to re-evaluate the total effect of each obesity exposure on BC overall (N_case_ = 133,384, N_control_ = 113,789). We further looked into its estrogen receptor (ER)-defined subtypes (N_ER+_ = 69,501, N_ER−_ = 21,468, N_control_ = 105,974). Multivariable MR (MVMR) was applied to estimate the independent causal effect of each obesity-related trait on BC taking into account confounders as well as to investigate the independent effect of adult and childhood obesity taking into account their inter-correlation.

**Results:**

In UVMR, significant protective effects of both adult BMI (OR = 0.89, 95%CI = 0.83-0.96) and childhood BMI (OR = 0.78, 95%CI = 0.70-0.87) were observed for BC overall. Comparable effects were found in ER+ and ER− subtypes. Similarly, genetically predicted adult WHR was also associated with a significantly decreased risk of BC overall (OR = 0.88, 95%CI = 0.80-0.98), restricting to ER+ subtype (OR = 0.88, 95%CI = 0.80-0.98). Conditional on childhood BMI, the effect of adult general obesity on BC overall attenuated to null (OR = 1.00, 95%CI = 0.90-1.10), while the effect of adult abdominal obesity attenuated to some extent (WHR: OR = 0.90, 95%CI = 0.82-0.98; WHR_adj_BMI: OR = 0.92, 95%CI = 0.86-0.99). On the contrary, an independent significant protective effect of childhood BMI was observed in BC overall, irrespective of adult measures (adjusted for adult BMI: OR = 0.86, 95%CI = 0.77-0.95; adjusted for adult WHR: OR = 0.86, 95%CI = 0.78-0.95; adjusted for adult WHR_adj_BMI: OR = 0.82, 95%CI = 0.75-0.90).

**Conclusions:**

While successfully replicating the inverse causal relationship between obesity-related exposures and risk of BC, our study demonstrated the protective effect of adult obesity to be largely (adult BMI) or partly (adult WHR or WHR_adj_BMI) attributed to childhood obesity. Our findings highlight an independent role of childhood obesity in affecting the risk of BC as well as the importance of taking into account the complex interplay underlying correlated exposures.

## Introduction

Obesity, a widely recognized public health challenge, plays a complex role in the development of female breast cancer (BC) ^1^, with associations differ depending on its distribution (e.g., general vs. abdominal) and timing (e.g., childhood vs. adulthood). Traditional epidemiological studies have consistently observed an increased risk of post-menopausal BC as well as a decreased risk of pre-menopausal BC to be associated with adult body-mass index (BMI, a measure of adult general obesity), while results for waist-to-hip ratio (WHR, a measure of adult abdominal obesity) and childhood BMI remain conflicting ^2–4^. These intricate associations motivate the need for understanding the causality and interaction of multiple obesity-related traits on BC risk.

Mendelian randomization (MR) is a powerful tool that uses genetic variants (single nucleotide polymorphisms, SNP) as instrumental variables (IVs) to make causal inference ^5^ and has been widely applied to determine the causal associations between obesity and BC risk ^6–10^. Using 15,748 BC cases and a limited number of IVs (77 SNPs for adult BMI; 15 SNPs for childhood BMI), Gao et al. conducted a univariable MR (UVMR) which found a suggestive protective effect of general obesity (adult BMI: OR = 0.66, 95%CI = 0.57-0.77; childhood BMI: OR = 0.71, 95%CI = 0.60-0.80) on BC overall ^7^. In an enlarged MR study conducted by Ooi et al. using 122,977 BC cases and the same number of IVs (77 SNPs for adult BMI as in Gao et al.), a consistent protective effect of BMI on BC overall was identified (OR = 0.81, 95%CI = 0.74-0.89). Such an effect remained significant in subtypes defined by estrogen receptor (ER) status (ER+: OR = 0.81, 95%CI = 0.74-0.89; ER−: OR = 0.78, 95%CI = 0.67-0.91) ^10^. Expanding the number of IVs into 166 or ~700 BMI-associated SNPs, another two studies drew similar conclusions ^6,8^. Opposite to general obesity, MR studies on abdominal obesity remain sparse. No significant association was detected for WHR ^7^, while a decreased risk of BC (OR = 0.85, 95%CI = 0. 79-0.91) was reported by Shu et al. for BMI-adjusted WHR (WHR_adj_BMI, representing abdominal body fat independent of general body fat) using 54 WHR_adj_BMI-associated SNPs ^6^. Furthermore, to understand the independent effect of correlated exposures on the outcome, multivariable MR^11^ (MVMR, an extension to UVMR) has been developed. In the hitherto only available MVMR study ^9^ which modeled simultaneously adult and childhood body size using composite IVs (191 SNPs for adult body size plus 124 SNPs for childhood body size), a protective effect of childhood body size with BC was observed conditioning on adult body size ^9^, while the protective effect of adult body size turned to null conditioning on childhood body size, highlighting the importance of taking into account multiple obesity-related traits over life-course simultaneously.

Despite existing MR studies having advanced our knowledge on an intrinsic link underlying obesity and BC, a few gaps need to be filled. First, most studies did not use female-specific IVs to match with a female disease BC – heterogeneity derived from sex-combined IVs would lead to a biased MR estimate ^12^. Second, existing studies using a handful of IVs were thus of poor statistical power – the most updated GWAS of BMI and WHR has identified a four-fold enlarged number of female-specific IVs, which would greatly improve the statistical power and the accuracy of estimates. Third, the only available MVMR study to date, used retrospective questionnaire-based categorized data for perceived childhood obesity, potentially yielding to measurement error. Last but not least, despite BC is a complex disease with distinct subtypes, most studies did not phenotype BC by ER status.

As the sample size of genome-wide association studies (GWASs) continues to grow and the data continue to accumulate ^13^, it is timely to conduct a comprehensive re-assessment on the causal role of obesity in BC through an MR design. Therefore, in this study, we used a largely increased set of sex-specific IVs derived from the hitherto largest GWAS(s) conducted for exposure and outcome ^13–16^ to (1) re-evaluate the total effect of obesity-related traits (general and abdominal obesity, adult and childhood obesity) on BC overall and its ER-defined subtypes; (2) estimate the independent causal effect of each obesity-related trait after accounting for the confounding effects from four major risk factors, including smoking, drinking, age at menarche (AAM) and age at natural menopause (ANM); (3) investigate the independent effect of adult and childhood obesity on BC taking into account their inter-correlation.

## Methods

### Data sources

#### Exposure GWAS(s)

The hitherto largest GWAS(s) ^13^ of general obesity (BMI) and abdominal obesity (WHR and WHR_adj_BMI) in adults were conducted via a collaborative effort of the UK Biobank (UKBB) and the Genetic Investigation of Anthropometric Traits (GIANT) consortium in 2019, including ~ 700,000 individuals of European ancestry. Anthropometric parameters, including height, weight, waist, and hip circumferences were measured according to standard protocols. BMI was calculated dividing weight by squared height and WHR was calculated dividing waist circumference by hip circumference. WHR_adj_BMI was generated from the regression of WHR on BMI by including BMI as an additional independent variable. Due to the large sample size, sex-specific analysis was performed, based on 434,794 women for BMI, 381,152 women for WHR and 379,501 women for WHR_adj_BMI.

As for childhood BMI, the latest and the largest GWAS was conducted by the Early Growth Genetics (EGG) consortium in 2020 ^14^ combining data of 41 studies, involving 39,620 children aged 6-10 years and of European ancestry. Unfortunately, sex-specific results of childhood BMI were not available due to data restrictions.

#### Outcome GWAS(s)

Summary-level data were available for three BC phenotypes – the overall BC, the ER+ and the ER− subtype. For BC overall, we retrieved data from the most updated GWAS conducted in 2020 involving 133,384 cases and 113,789 controls of European ancestry combining results from 82 studies participating the Breast Cancer Association Consortium (BCAC) and 11 other breast cancer genetic studies ^16^. This GWAS expanded upon a previous BCAC GWAS ^15^ (2017) with an additional 10,407 cases and 7,815 controls (10% increase), and identified 32 novel susceptibility loci upon the previously detected 153 loci.

For BC subtypes, we used data from a previous BCAC GWAS^15^ (2017) including 69,501 ER+ cases, 21,468 ER− cases and 105,974 controls, which is the hitherto largest GWAS performed for ER subtypes.

#### Other GWAS(s)

We included four risk factors (AAM, ANM, smoking and drinking) as potential confounders to be controlled for in our MR study. For AAM, we used GWAS from the Reproductive Genetics (ReproGen) consortium published in 2017 comprising 329,345 European women from 40 participating studies (N = 179,117), 23andMe (N = 76,831) and UKBB (N = 73,397)^17^. For ANM, we used the most updated GWAS from ReproGen in 2021 involving data of 201,323 European women^18^. For smoking and drinking, we used data published in 2019 from GWAS & Sequencing Consortium of Alcohol and Nicotine (GSCAN), with 1,232,091 participants for smoking initiation and 941,280 participants for drinks per week^19^, of all European ancestry.

#### Instrument selection

We extracted IVs that reached genome-wide significance from GWASs for the exposures. This yielded to 281 independent SNPs for BMI, 203 independent SNPs for WHR, 266 independent SNPs for WHR_adj_BMI (all restricted to females, *P*-value < 5×10^−9^) and 25 independent SNPs for childhood BMI (both sexes combined, *P*-value < 5×10^−8^). We then matched and harmonized these SNPs with the outcome GWAS (BC overall and its ER-defined subtypes). For details please also see Supplementary Table 1.

To avoid weak instrument bias, we calculated the strength of instruments (Table 1) using the formula $\text{F}=(\frac{\text{N}-\text{K}-1}{\text{K}})(\frac{{\text{R}}^{2}}{1-{\text{R}}^{2}})$. An instrument was considered sufficiently strong if a corresponding F-statistic was larger than 10 ^20^. R^2^ (phenotypic variance explained by genetic instruments) was extracted from the original GWAS or calculated using $\hat{\beta }$ (estimated genetic association of SNP with the exposure) and MAF via the formula ${\text{R}}^{2}=\sum^{\text{​}}2\times {\hat{\beta }}^{2}\times \text{MAF}\times (1-\text{MAF})$.

### Statistical analysis

A comprehensive two-sample MR analysis was performed to evaluate a putative causal relationship between exposures (BMI, WHR, WHR_adj_BMI, childhood BMI) and outcomes (BC overall, ER+ and ER− subtypes), with an analytical schematic diagram presented in Supplementary Figure 1.

#### Univariable Mendelian randomization analysis

To investigate the total effect of each obesity-related trait on BC, UVMR was conducted as our primary analysis. We first employed an inverse-variance weighted (IVW) approach to estimate the causal effect by regressing the outcome effect coefficient on the exposure effect coefficient with no intercept term ^21^. Considering the potential bias derived from horizontal pleiotropy of instruments, we complemented IVW with MR-Egger regression ^22^ and weighted-median approach ^23^. MR-Egger regression is largely similar to IVW except its regression model contains intercept to reflect directional pleiotropy. Weighted-median approach is more robust to invalid IVs compared to IVW and MR-Egger regression. Moreover, we also implemented MR-PRESSO (Mendelian Randomization Pleiotropy Residual Sum and Outlier) to evaluate the presence of horizontal pleiotropy and to re-evaluate the causal effect after removing the detected outlying SNPs ^24^. A putative total causal effect was considered if estimates showed statistical significance (*P*-value < 0.05) in any of these four methods and maintained directional consistency in the remaining methods.

Several sensitivity analyses were conducted to assess the robustness of results, including (i) analysis using IVs excluding palindromic SNPs with strand ambiguity; (ii) analysis using IVs excluding pleiotropic SNPs that were associated with the potential confounding traits (AAM, ANM, smoking and drinking) according to GWAS Catalog; (iii) Leave-one-out analysis where each SNP was removed sequentially to identify outliers that might bias the MR estimates ^25^.

In addition, a bidirectional MR analysis was also performed to evaluate if a genetic predisposition to BC would influence obesity. We collected all previously reported IVs reaching genome-wide significance (*P*-value < 5×10^−8^) in the BC GWAS published in 2020 by BCAC ^16^.

#### Multivariable Mendelian randomization analysis

To further evaluate whether the causal effects of obesity on BC are affected by major confounders, and whether the casual effects of childhood and adult obesity on BC are independent of each other, we conducted two additional analyses in the framework of MVMR^11,26^:

(i) Four risk factors (AAM, ANM, smoking and drinking), believed as important confounders of the association between obesity and BC, were incorporated together with the exposures, one at a time as well as simultaneously to estimate the independent effect of each exposure on BC after accounting for the confounding effects. We removed SNPs in linkage disequilibrium (r^2^ < 0.001) to obtain independent variants of composite IVs. Stratified analysis on ER subtypes was performed following the same procedure.
(ii) Considering the inter-correlation among adult and childhood obesity, childhood BMI was incorporated with each adult obesity trait (BMI, WHR, WHR_adj_BMI) to examine their independent effect on BC. Three sets of composite IVs after a linkage disequilibrium clumping with r^2^ > 0.001 were used ^27^. These included composite IVs involving 270 SNPs utilized for BMI and childhood BMI, 208 SNPs for WHR and childhood BMI, and 266 SNPs for WHR_adj_BMI and childhood BMI. Stratified analysis on ER subtypes was performed following the same procedure.

In our MR analysis, *P*-values were transformed to q-values to account for the false discovery rate (FDR) in multiple tests. Statistical significance was defined as FDR-adjusted *P*-value less than 0.05, and marginal significance was defined as crude *P*-value less than 0.05 and FDR-adjusted *P*-value more than 0.05. We conducted UVMR using package “TwoSampleMR” (version 0.5.6) and MVMR using package “MendelianRandomization” (version 0.5.1) in software R (version 4.1.0).

#### Genetic correlation analysis

To understand the shared genetic basis between exposures and outcomes, a genome-wide genetic correlation analysis was further conducted. Full set GWAS summary data were used to estimate genome-wide genetic correlations (r_g_), which quantifies the intrinsic average sharing of genetic effect between pairs of traits that is independent of environmental factors ^28^. An algorithm implemented in software linkage-disequilibrium score regression (LDSC) was adopted to perform regression on the product of z-scores cross any two traits leveraging SNPs across the whole genome ^29^. Statistical Significance of genetic correlations was defined as *P*-value less than 0.05.

## Results

The basic characteristics of each GWAS dataset and IVs are shown in Table 1. Current IVs explained about 4% of the phenotypic variance of each exposure (4.0% for adult BMI with 281 index SNPs; 4% for WHR with 203 index SNPs; 3.6% for WHR_adj_BMI with 266 index SNPs; 3.6% for childhood BMI with 25 index SNPs). F-statistics for these IVs ranged from 53 to 78, suggesting strong instruments.

**Figure 1:**
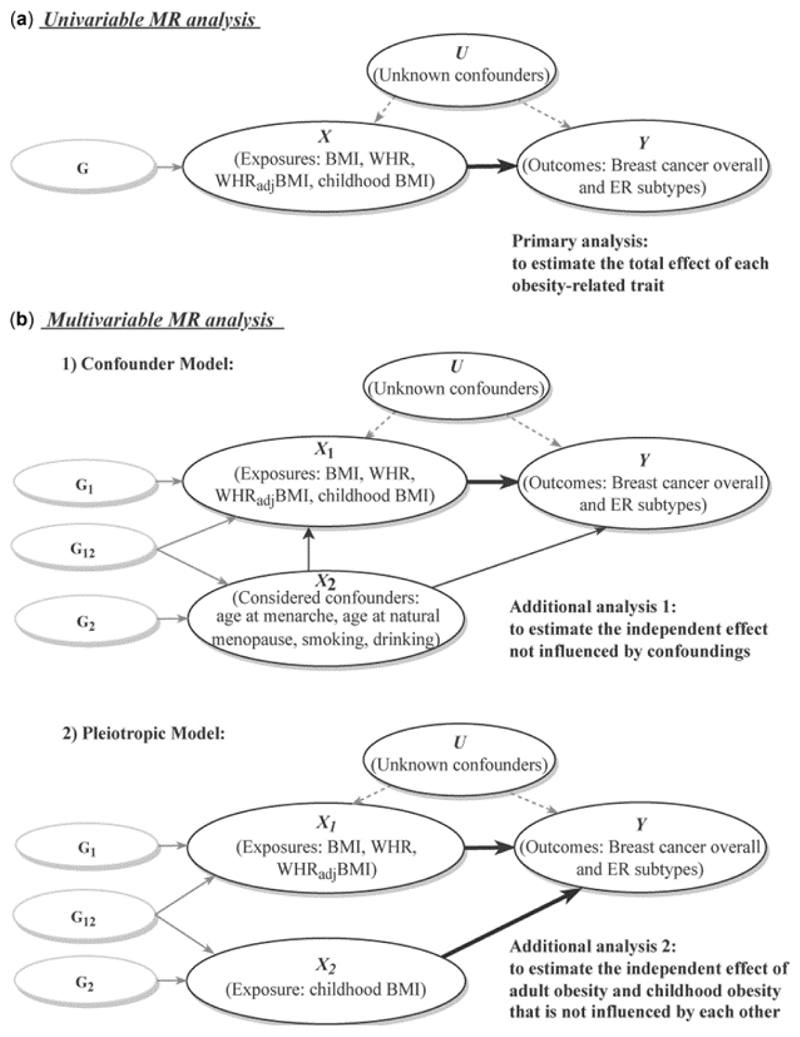
Analytical schematic diagram of the Mendelian randomization (MR) analysis implemented in this study (a) Univariable MR analysis; (b) multivariable MR analysis, including two models: (i) confounder model; (ii) pleiotropic model. G represents genetic variants (single-nucleotide polymorphisms, SNPs) that reliably predict the exposure variable (X) and are used as instrumental variables to represent exposure. G1 and G2 represent SNPs that specifically affect X1 and X2, respectively, whereas G12 represents SNPs that affect both X1 and X2 simultaneously. Thick lines illustrate the causal effect confirmed by the current analysis. BMI, body mass index; WHR, waist-to-hip ratio; WHRadjBMI, waist-to-hip ratio adjusted for body mass index; ER, oestrogen receptor

As shown in Figure 1, using UVMR, genetically predicted female-specific BMI presented a statistically significant association with a decreased risk of BC overall (OR = 0.89, 95%CI = 0.83-0.96). Such an effect remained consistent in both ER+ (OR = 0.90, 95%CI = 0.83-0.97) and ER− subtypes (OR = 0.85, 95%CI = 0.76-0.95), all survived FDR correction. Similar findings were observed for genetically predicted female-specific WHR on a decreased risk of BC overall (OR = 0.87, 95%CI = 0.80-0.96), as well as for ER+ subtype (OR = 0.88, 95%CI = 0.80-0.98), but not for ER− subtype. When the effect of BMI was removed from WHR (WHR_adj_BMI), the observed inverse association of WHR reduced to some extent with marginal significance (*P*-value < 0.05) in BC overall (OR = 0.94, 95%CI = 0.88-1.00) and in ER+ subtype (OR = 0.92, 95%CI = 0.86-1.00). As for childhood BMI, strong evidence on a statistically significant protective effect was observed consistent across all BC phenotypes (overall: OR = 0.78, 95%CI = 0.70-0.87; ER+: OR = 0.80, 95%CI = 0.72-0.89; ER-: OR = 0.71, 95%CI = 0.60-0.85). All these aforementioned results derived from IVW were further supported by the weighed-median approach and the MR-Egger regression, with estimates consistent in direction and without apparent sign of horizontal pleiotropy (Supplementary Table 2). Results from MR-PRESSO using outlier-corrected method were also highly consistent with those from IVW.

**Figure 2:**
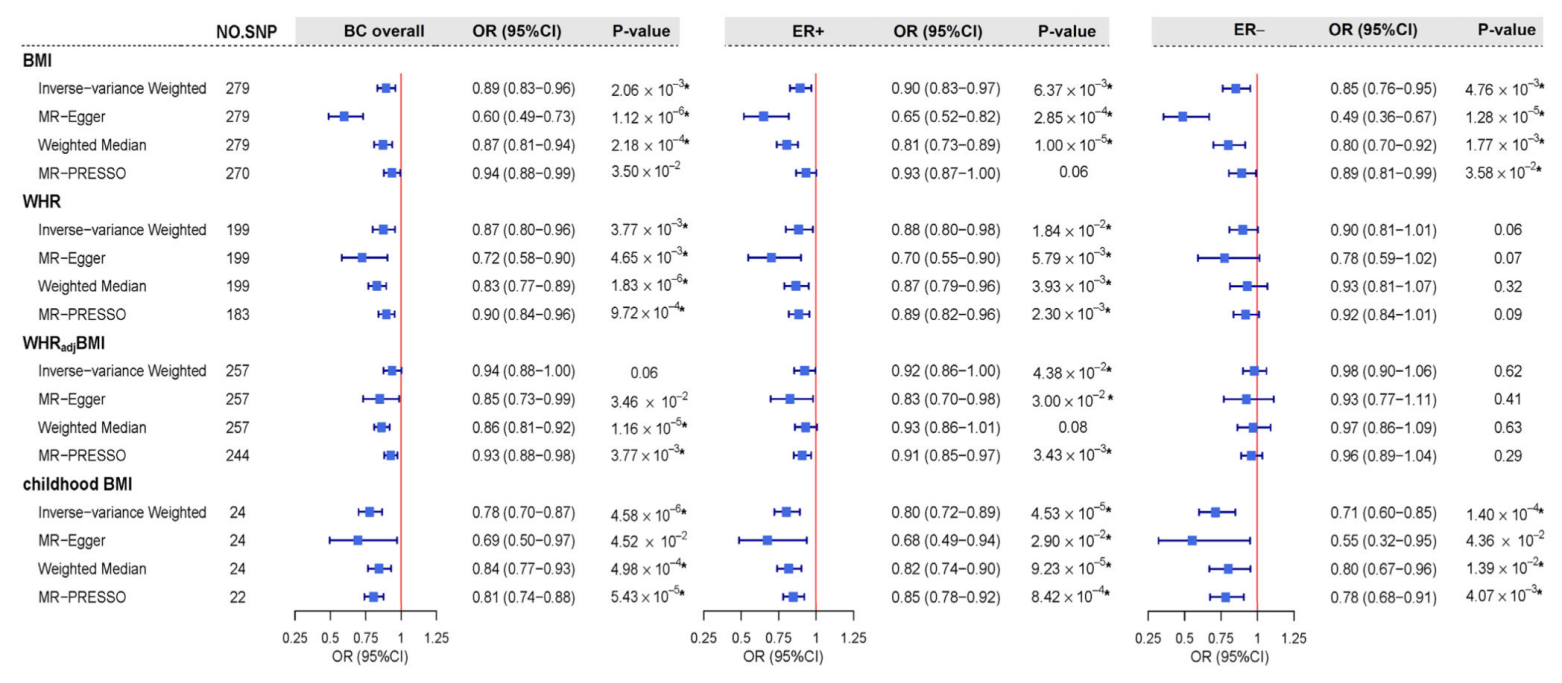
Estimated total effects of obesity-related traits on the risk of BC using univariable Mendelian randomization. Boxes denote the point estimates of causal effects, and error bars denote 95% confidence intervals. Asterisks (*) denote statistical significance survived false discovery rate (FDR) correction (*P*_FDR_ <0.05). Inverse-variance weighted approach was used as primary analysis; MR-Egger, weighted-median and MR-PRESSO were used as sensitivity analyses. Abbreviations: BMI, body mass index; WHR, waist-to-hip ratio; WHR_adj_BMI, waist-to-hip ratio adjusted for body mass index; BC, breast cancer; ER, estrogen receptor; AAM, age at menarche; ANM, age at natural menopause; NO. SNP, number of instrumental variables; OR, odds ratio; 95%CI, 95% confidence interval.

Sensitivity analyses excluding pleiotropic SNPs or palindromic SNPs, as well as the leave-one-out analysis, showed similar findings, demonstrating the robustness of our results (Supplementary Figure 2, Supplementary Figure 3). Additionally, reverse-direction MR did not find a genetic predisposition to BC overall to be associated with any of the obesity traits (Supplementary Table 3). Considering important mediatory phenotypes or risk factors that may affect the relationship between obesity and BC, we performed an MVMR analysis by incorporating each exposure with confounders (AAM, ANM, smoking and drinking), separately and together. The effect for each obesity trait on BC remained consistent in both direction and magnitude after adjusting for confounders, all survived multiple testing corrections (Figure 2).

Despite our prior results providing evidence on that both childhood and adult obesity contribute to a decreased risk of BC, their independent effects remain unclear. We conducted a series of MVMR to examine whether the casual effects of childhood and adult obesity on BC are independent of each other (Table 2). Notably, the effect of adult BMI on BC overall attenuated to null in MVMR after adjusting for childhood BMI (OR = 1.00, 95%CI = 0.90-1.10), suggesting the effect of adult general obesity on BC is influenced by childhood obesity. On the contrary, the decreased risk of adult abdominal obesity (WHR and WHR_adj_BMI) with BC overall attenuated slightly but remained statistically significant when conditional on childhood BMI (WHR: OR = 0.90, 95%CI = 0.82-0.98; WHR_adj_BMI: OR = 0.92, 95%CI = 0.86-0.99). Similar results were also identified in ER+ (WHR: OR = 0.90, 95%CI = 0.81-1.00; WHR_adj_BMI: OR = 0.91, 95%CI = 0.84-0.98), but not in ER− subtype. These results indicated the effect of adult abdominal obesity on BC to be partially independent of childhood obesity, suggesting multiple distinct pathways influencing BC susceptibility. On the contrary, a strong independent and statistical significant effect of childhood BMI was consistently observed in BC overall when conditional on each adult obesity trait (adjusted for adult BMI: OR = 0.84, 95%CI = 0.77-0.93; adjusted for adult WHR: OR = 0.84, 95%CI = 0.76-0.91; adjusted for adult WHR_adj_BMI: OR = 0.80, 95%CI = 0.74-0.87), and the effect remained significant across both ER+ subtype (adjusted for adult BMI: OR = 0.86, 95%CI = 0.77-0.95; adjusted for adult WHR: OR = 0.86, 95%CI = 0.78-0.95; adjusted for adult WHR_adj_BMI: OR = 0.82, 95%CI = 0.75-0.90) and ER− subtype (adjusted for adult BMI: OR = 0.83, 95%CI = 0.72-0.96; adjusted for adult WHR: OR = 0.76, 95%CI = 0.68-0.85; adjusted for adult WHR_adj_BMI: OR = 0.74, 95%CI = 0.67-0.82).

**Figure 3:**
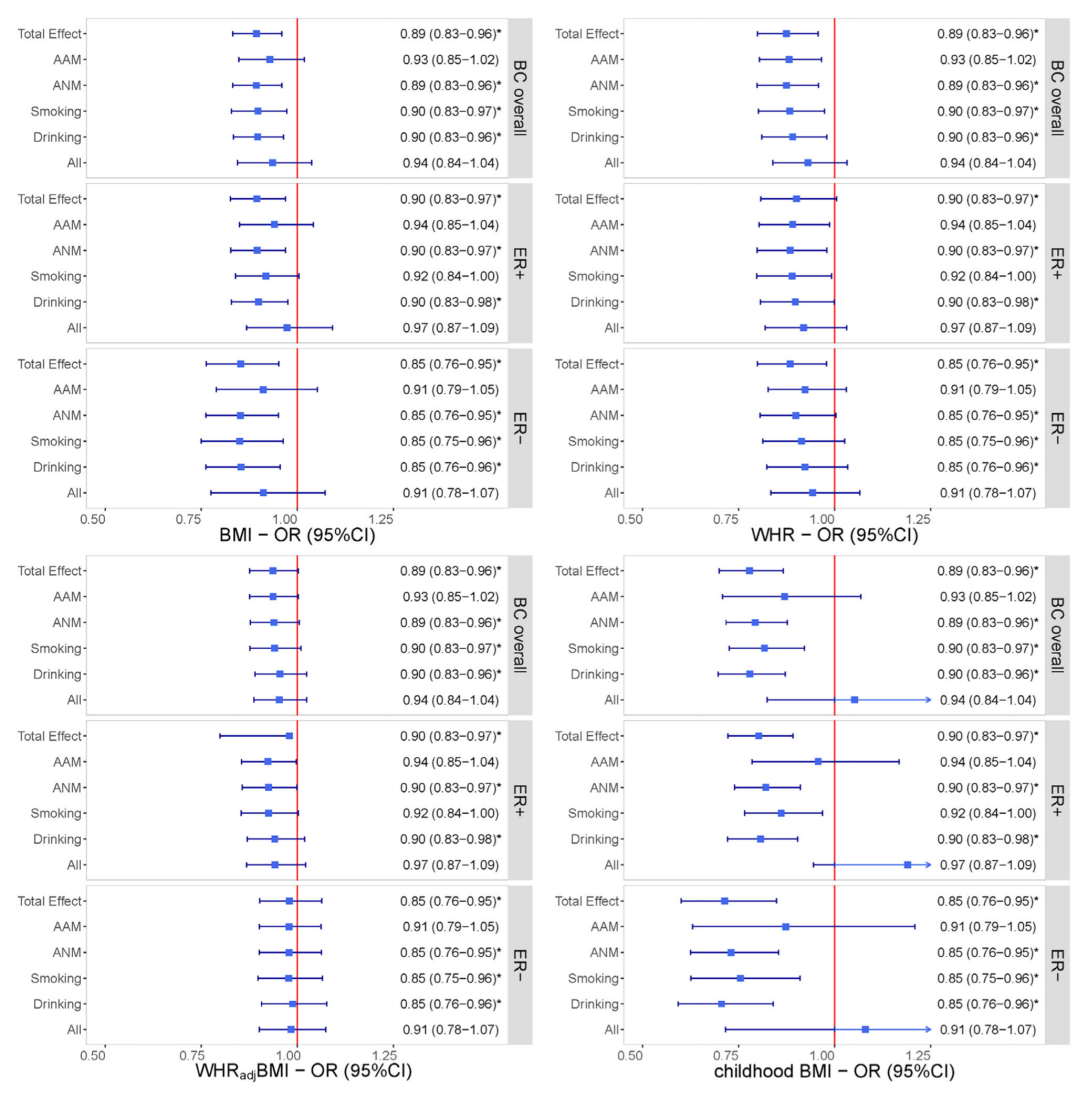
Independent effects of genetically predicted obesity-related traits on the risk of BC after adjusting for each confounder separately and together using multivariable Mendelian randomization. The y-axis details the genetically predicted confounder(s) for which adjustment was made, and the x-axis details the ORs and 95%CIs per 1-standard deviation (SD) increase in exposure. Asterisks (*) denote statistical significance survived false discovery rate (FDR) correction (*P*_FDR_ <0.05). Total effect refers to the estimate derived from UVMR. Abbreviations: BMI, body mass index; WHR, waist-to-hip ratio; WHR_adj_BMI, waist-to-hip ratio adjusted for body mass index; BC, breast cancer; ER, estrogen receptor; AAM, age at menarche; ANM, age at natural menopause; OR, odds ratio; 95%CI, 95% confidence interval.

Finally, we evaluated the shared genetic basis between pairs of exposure and outcome using SNPs across the whole genome. As shown in Figure 3, we found a significant negative genetic correlation of childhood BMI with BC overall (r_g_ = −0.06, *P*-value = 4.98×10^−2^) as well as with ER+ subtype (rg = −0.08, P-value = 1.82×10^−2^) (Supplementary Table 4).

## Discussion

Our MR study revisited the causal role of multiple obesity-related traits in the development of BC overall as well as its ER-defined subtypes, utilizing data from the hitherto largest GWAS(s) conducted for each trait. By incorporating a set of four-fold enlarged female-specific IVs, both the precision and accuracy of our MR estimates were substantially improved. We successfully replicated the significant protective effects of genetically predicted adult BMI, adult WHR and childhood BMI on BC. We then identified a marginally significant protective effect of WHR_adj_BMI on BC. Integrating these obesity-related traits together, we further found the effect of adult BMI on BC was largely attributed to childhood BMI, while the effect of adult WHR (or WHR_adj_BMI) was partly dependent on childhood BMI. On the contrary, childhood BMI consistently showed an independent protective effect on BC irrespective of adult measures. Additionally, subtype-specific analysis suggested that the significant effects of adult and childhood BMI held true for both ER+ and ER− subtypes, while the effects of WHR and WHR_adj_BMI were only restricted to ER+ subtypes.

Despite several studies that have applied an MR approach to discover associations between genetically predicted general obesity and BC ^6,7,9,10^, our work presents a comprehensive reconsideration of these associations. First, compared with previous MR, we used an enlarged set of female-specific instruments involving 281 adult BMI-associated variants explaining 4.0% of the phenotypic variance, greatly enhancing the statistical power. Second, we took into consideration potential influence from important confounders, which previous MR did not have the opportunity for. The consistent protective effect of adult BMI on BC overall with and without conditioning on confounders provided convincing evidence on a putative causal relationship. Third, using MVMR, we further controlled for the effect of childhood BMI and found a mitigation on the effect of adult BMI, indicating the identified putative causal relationship for adult BMI and BC to be largely attributed to a high childhood BMI. These findings were supported by a previous MVMR ^9^ conducted based on data from UKBB and BCAC (adult body size: OR_Uvmr_ = 0.82, *P* = 8.04×10^−4^ vs. OR_mvmr_ = 1.08, *P* = 0.32) – while they used questionnaire-based perceived obesity in age 10, we used actual measured obesity among children, minimizing the likelihood of misclassification. Collectively, these findings suggest a complex interplay underlying multiple obesity-related traits over life-course, highlighting the importance of taking into consideration these traits simultaneously.

Two previous UVMR(s) attempted to examine the role of genetically predicted abdominal obesity in BC. One used 14 sex-combined instruments of WHR and concluded a null association ^7^, while the other used 54 sex-combined instruments of WHR_adj_BMI and reported a decreased effect with BC overall ^6^. Our UVMR, using an expanded set of IVs involving 203 WHR-associated female-specific SNPs confirmed a significant protective effect with BC overall. Our MVMR further observed that this protective effect of WHR remained significant even after adjusting for confounders and adult BMI (WHR_adj_BMI), while reduced to some extent after adjusting for childhood BMI. Notably, such a relationship – an increased abdominal obesity associating with a decreased risk of BC – conflicts observational studies which identified a positive association for postmenopausal BC ^30,31^ and an inconsistent association for premenopausal BC ^32–34^. One potential interpretation to such discrepancy could be that genetically predicted WHR and WHR_adj_BMI primarily reflect excessive visceral adipose tissue deposition by affecting genetic predisposition in early life, rather than in late adulthood. To the best of our knowledge, the effect of adult abdominal obesity on BC in observational studies was often modified by obesogenic environment ^35^, such as sugar-sweetened beverages, fried foods and physical inactivity, the majority of which were unlikely to be captured by our study using genetic instruments as proxies. Further experimental studies are warranted to clarify the detailed molecular mechanism underlying this finding.

Our study highlights a non-trivial role of childhood BMI in the development of BC. The significant protective effect from our UVMR was largely in line with previous work ^7,9^, while results of our MVMR provided strong evidence for an independent causal association of childhood obesity with BC overall irrespective of adult measures. Furthermore, genetic correlation analysis confirmed a significant negative shared genetic basis, indicating a higher genetically predicted childhood BMI to be correlated with a decreased susceptibility of breast cancer carcinogenesis. Our results corroborate the findings of prospective cohort studies showing an inverse relationship between childhood BMI and BC ^36,37^. Potential mechanisms include a decreased frequency of ovulatory cycles ^38^ and earlier breast differentiation due to higher levels of estrogens derived from adipose tissues in obese children, terminally decreasing the susceptibility to malignant transformation ^39^.

Subtype-specific analyses provide implications for understanding the biological mechanisms linking genetically predicted obesity with BC risk. In our study, while the effect of BMI in both childhood and adult on BC did not differ across ER-defined subtypes (regardless of conditional analysis), the protective effects of genetically predicted adult WHR and WHR_adj_BMI were found to be restricted to ER+ subtype (consistent across all conditional analysis adjusting for childhood obesity and confounders). Obesity is known to profoundly affect estrogens metabolism, and fat-derived estrogens are considered a principal biological mechanism through which abdominal obesity impacts mainly the risk of ER+ but not ER− subtype ^40^.

This is a comprehensive MR conducted to interrogate the independent role of multiple correlated obesity traits in BC, using the hitherto largest female-specific data with an almost four-times increased number of instruments and a doubled phenotypic variance explained compared with previous studies, substantially improving statistical power whilst reducing reverse causation ^41^. However, we need to acknowledge several limitations. First, although we adopted female-specific instruments of each adult obesity-related trait to match with female-specific cancer, we were unable to estimate the sex-specific effect of childhood obesity due to data restrictions. Given that the sex instrumental heterogeneity has been recently confirmed to have a non-ignorable impact on the estimates of two-sample MR ^12^, future investigations would benefit from developing girl-specific IVs of childhood obesity. Second, pleiotropy derived from undetected confounders might bias the causal estimates. However, we tried to reduce such bias to the best of our ability. The directional consistent results derived from multiple ‘pleiotropy-robust’ methods ^42^ supported the validity of our MR results. Lastly, as two-sample MR approach is typically based on linear assumption, we were unable to examine the nonlinear obesity-BC relationship based on GWAS summary statistics, such as the N-shaped association which was identified in traditional epidemiological studies ^45,46^. Future one-sample MR studies using semiparametric methods ^44^ are perhaps warranted.

To conclude, our comprehensive MR study with an enlarged sample size successfully replicated the inverse relationship of obesity with the risk of BC. We further identified that the total effect of adult general obesity on BC was largely attributed to childhood obesity, while that of adult abdominal obesity was at least partly attributed to childhood obesity. Finally, we demonstrated a predominantly independent effect of childhood BMI in affecting BC onset, irrespective of adult measures. Our findings highlight an important role of early life obesity in affecting the development of BC later on as well as the importance of taking into account the complex interplay underlying correlated exposures.

## Supplementary Material

Figures, tables, and supplementary information

## Figures and Tables

**Table 1:** Description of GWAS datasets and instrumental variables used in our study.

Phenotype	IV	Sample size	Ethnicity	Consortium	R^2^	F-statistics	Author, Year
**Exposures**							
BMI	281	434,794 females	European	Genetic Investigation of ANthropometric Traits (GIANT) and UK BioBank	0.040	63.799	Pulit, 2019
WHR	203	381,152 females	European	Genetic Investigation of ANthropometric Traits (GIANT) and UK BioBank	0.040	78.191	Pulit, 2019
WHR_adj_BMI	266	379,501 females	European	Genetic Investigation of ANthropometric Traits (GIANT) and UK BioBank	0.036	53.242	Pulit, 2019
childhood BMI	25	39,620	European	Early Growth Genetics (EGG)	0.036	58.975	Vogelezang, 2020
**Outcomes**							
BC overall	170	133,384 cases / 113,789 controls	European	Breast Cancer Association Consortium (BCAC)	0.067	104.867	Zhang, 2020
ER+	NA	69,501 cases / 105,974 controls	European	Breast Cancer Association Consortium (BCAC)	NA	NA	Michailidou, 2017
ER-	NA	21,468 cases / 105,974 controls	European	Breast Cancer Association Consortium (BCAC)	NA	NA	Michailidou, 2017
**Confounders**							
AAM	375	329,345	European	Reproductive Genetics (ReproGen)	0.074	67.656	Day, 2017
ANM	290	201,323	European	Reproductive Genetics (ReproGen)	0.130	103.187	Ruth, 2021
Smoking	378	1,232,091	European	GWAS & Sequencing Consortium of Alcohol and Nicotine (GSCAN)	0.023	77.222	Liu, 2019
Drinking	99	941,280	European	GWAS & Sequencing Consortium of Alcohol and Nicotine (GSCAN)	0.002	17.811	Liu, 2019

Abbreviations: GWAS, genome-wide association study; IV, instrumental variable; BMI, body mass index; WHR, waist-to-hip ratio; WHR_adj_BMI, waist-to-hip ratio adjusted for body mass index; BC, breast cancer; ER, estrogen receptor; AAM, age at menarche; ANM, age at natural menopause.

**Table 2:** Independent effect of adult obesity and childhood obesity on the risk of BC using multivariable Mendelian randomization analysis

	BC overall			ER+			ER-	
	OR (95% CI)	*P-value*		OR (95% CI)	*P-value*		OR (95% CI)	*P-value*
**Model 1**								
BMI	1.00 (0.90-1.10)	0.96		0.99 (0.88-1.11)	0.88		0.96 (0.82-1.12)	0.58
childhood BMI	0.84 (0.77-0.93)	3.93×10^-4^*		0.86 (0.77-0.95)	4.05×10^-3^*		0.83 (0.72-0.96)	1.43×10^-2^*
**Model 2**								
WHR	0.90 (0.82-0.98)	1.49×10^-2^*		0.90 (0.81-1.00)	4.29×10^-2^		0.93 (0.84-1.04)	0.20
childhood BMI	0.84 (0.76-0.91)	6.57×10^-5^*		0.86 (0.78-0.95)	2.87×10^-3^*		0.76 (0.68-0.85)	6.10×10^-7^*
**Model 3**								
WHR_adj_BMI	0.92 (0.86-0.99)	1.98×10^-2^*		0.91 (0.84-0.98)	1.92×10^-2^*		0.96 (0.88-1.05)	0.35
childhood BMI	0.80 (0.74-0.87)	1.24×10^-7^*		0.82 (0.75-0.90)	3.40×10^-5^*		0.74 (0.67-0.82)	6.09×10^-9^*

Model 1: independent effect of adult BMI and childhood BMI on BC; Model 2: independent effect of adult WHR and childhood BMI on BC; Model 3: independent effect of adult WHR_adj_BMI and childhood BMI on BC. Asterisks (*) denote statistical significance survived false discovery rate (FDR) correction (*P*_FDR_ <0.05). Abbreviations: BMI, body mass index; WHR, waist-to-hip ratio; WHR_adj_BMI, waist-to-hip ratio adjusted for body mass index; BC, breast cancer; ER, estrogen receptor; OR, odds ratio; 95%CI, 95% confidence interval.
