# Supplementary material for: Reassessing the causal role of obesity in breast cancer susceptibility – a comprehensive multivariable Mendelian randomization investigating the distribution and timing of exposure
Source: Int J Epidemiol. Author manuscript; Available in PMC 2023 Feb 9. (PMC7614158; doi:10.1093/ije/dyac143)
Supplement: Figures, tables, and supplementary information [file EMS153930-supplement-Figures__tables__and_supplementary_information.docx]

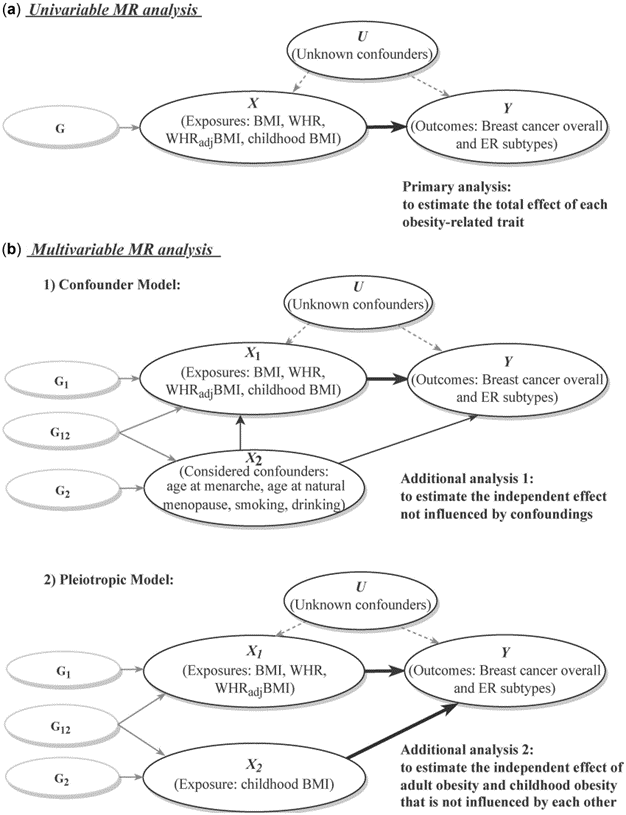
 **Figure 1** Analytical schematic diagram of the Mendelian randomization (MR) analysis implemented in this study

(a) Univariable MR analysis; (b) multivariable MR analysis, including two models: (i) confounder model; (ii) pleiotropic model.

G represents genetic variants (single-nucleotide polymorphisms, SNPs) that reliably predict the exposure variable (X) and are used as instrumental variables to represent exposure. G1 and G2 represent SNPs that specifically affect X1 and X2, respectively, whereas G12 represents SNPs that affect both X1 and X2 simultaneously. Thick lines illustrate the causal effect confirmed by the current analysis.

BMI, body mass index; WHR, waist-to-hip ratio; WHRadjBMI, waist-to-hip ratio adjusted for body mass index; ER, oestrogen receptor

| **Table 1.** Description of GWAS datasets and instrumental variables used in our study. | | | | | | | |
| --- | --- | --- | --- | --- | --- | --- | --- |
| Phenotype | IV | Sample size | Ethnicity | Consortium | R^2^ | F-statistics | Author, Year |
| **Exposures** | | | | | | | |
| BMI | 281 | 434,794 females | European | Genetic Investigation of ANthropometric Traits (GIANT) and UK BioBank | 0.040 | 63.799 | Pulit, 2019 |
| WHR | 203 | 381,152 females | European | Genetic Investigation of ANthropometric Traits (GIANT) and UK BioBank | 0.040 | 78.191 | Pulit, 2019 |
| WHR_adj_BMI | 266 | 379,501 females | European | Genetic Investigation of ANthropometric Traits (GIANT) and UK BioBank | 0.036 | 53.242 | Pulit, 2019 |
| childhood BMI | 25 | 39,620 | European | Early Growth Genetics (EGG) | 0.036 | 58.975 | Vogelezang, 2020 |
| **Outcomes** | | | | | | | |
| BC overall | 170 | 133,384 cases / 113,789 controls | European | Breast Cancer Association Consortium (BCAC) | 0.067 | 104.867 | Zhang, 2020 |
| ER+ | NA | 69,501 cases / 105,974 controls | European | Breast Cancer Association Consortium (BCAC) | NA | NA | Michailidou, 2017 |
| ER– | NA | 21,468 cases / 105,974 controls | European | Breast Cancer Association Consortium (BCAC) | NA | NA | Michailidou, 2017 |
| **Confounders** | | | | | | | |
| AAM | 375 | 329,345 | European | Reproductive Genetics (ReproGen) | 0.074 | 67.656 | Day, 2017 |
| ANM | 290 | 201,323 | European | Reproductive Genetics (ReproGen) | 0.130 | 103.187 | Ruth, 2021 |
| Smoking | 378 | 1,232,091 | European | GWAS & Sequencing Consortium of Alcohol and Nicotine (GSCAN) | 0.023 | 77.222 | Liu, 2019 |
| Drinking | 99 | 941,280 | European | GWAS & Sequencing Consortium of Alcohol and Nicotine (GSCAN) | 0.002 | 17.811 | Liu, 2019 |
| Abbreviations: GWAS, genome-wide association study; IV, instrumental variable; BMI, body mass index; WHR, waist-to-hip ratio; WHR_adj_BMI, waist-to-hip ratio adjusted for body mass index; BC, breast cancer; ER, estrogen receptor; AAM, age at menarche; ANM, age at natural menopause. | | | | | | | |


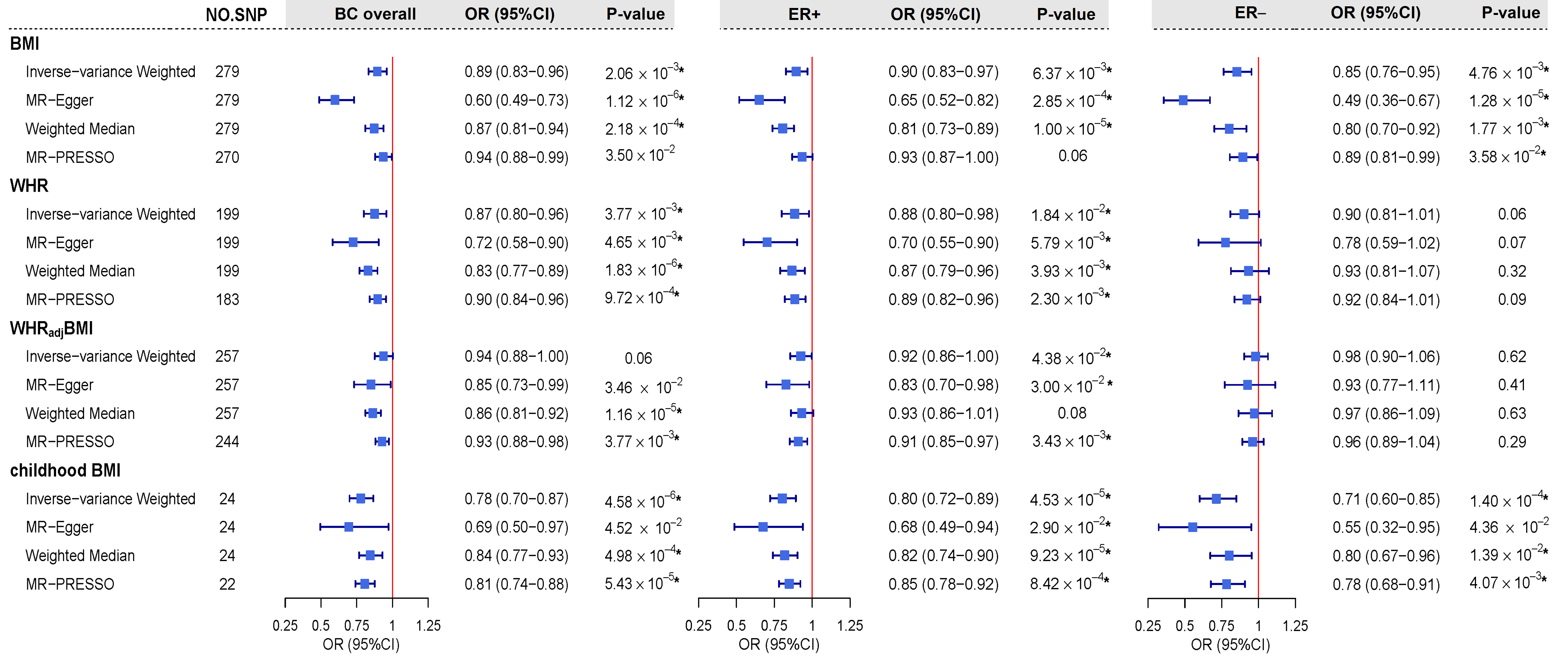


**Figure 2.** Estimated total effects of obesity-related traits on the risk of BC using univariable Mendelian randomization. Boxes denote the point estimates of causal effects, and error bars denote 95% confidence intervals. Asterisks (*) denote statistical significance survived false discovery rate (FDR) correction (*P*_FDR_ <0.05). Inverse-variance weighted approach was used as primary analysis; MR-Egger, weighted-median and MR-PRESSO were used as sensitivity analyses.

Abbreviations: BMI, body mass index; WHR, waist-to-hip ratio; WHR_adj_BMI, waist-to-hip ratio adjusted for body mass index; BC, breast cancer; ER, estrogen receptor; AAM, age at menarche; ANM, age at natural menopause; NO. SNP, number of instrumental variables; OR, odds ratio; 95%CI, 95% confidence interval.


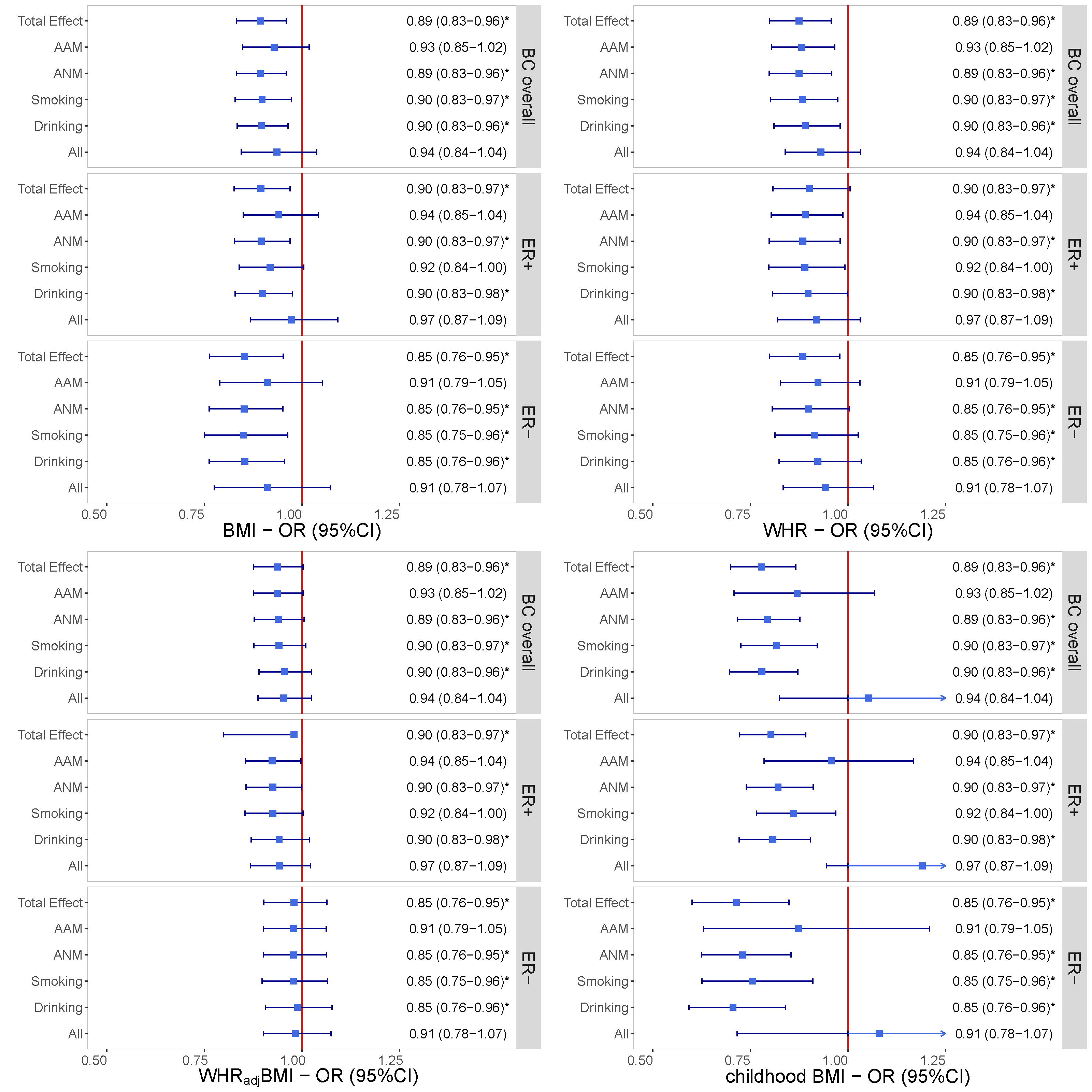


**Figure 3.** Independent effects of genetically predicted obesity-related traits on the risk of BC after adjusting for each confounder separately and together using multivariable Mendelian randomization. The y-axis details the genetically predicted confounder(s) for which adjustment was made, and the x-axis details the ORs and 95%CIs per 1-standard deviation (SD) increase in exposure. Asterisks (*) denote statistical significance survived false discovery rate (FDR) correction (*P*_FDR_ <0.05). Total effect refers to the estimate derived from UVMR.

Abbreviations: BMI, body mass index; WHR, waist-to-hip ratio; WHR_adj_BMI, waist-to-hip ratio adjusted for body mass index; BC, breast cancer; ER, estrogen receptor; AAM, age at menarche; ANM, age at natural menopause; OR, odds ratio; 95%CI, 95% confidence interval.

|  | | | | | | | | |
| --- | --- | --- | --- | --- | --- | --- | --- | --- |
| **Table 2.** Independent effect of adult obesity and childhood obesity on the risk of BC using multivariable Mendelian randomization analysis | | | | | | | | |
|  | BC overall | |  | ER+ | |  | ER– | |
|  | OR (95% CI) | *P-value* |  | OR (95% CI) | *P-value* |  | OR (95% CI) | *P-value* |
| **Model 1** | | | | | | | | |
| BMI | 1.00 (0.90-1.10) | 0.96 |  | 0.99 (0.88-1.11) | 0.88 |  | 0.96 (0.82-1.12) | 0.58 |
| childhood BMI | 0.84 (0.77-0.93) | 3.93×10^-4^* |  | 0.86 (0.77-0.95) | 4.05×10^-3^* |  | 0.83 (0.72-0.96) | 1.43×10^-2^* |
| **Model 2** |  |  |  |  |  |  |  |  |
| WHR | 0.90 (0.82-0.98) | 1.49×10^-2^* |  | 0.90 (0.81-1.00) | 4.29×10^-2^ |  | 0.93 (0.84-1.04) | 0.20 |
| childhood BMI | 0.84 (0.76-0.91) | 6.57×10^-5^* |  | 0.86 (0.78-0.95) | 2.87×10^-3^* |  | 0.76 (0.68-0.85) | 6.10×10^-7^* |
| **Model 3** | | | | | | | | |
| WHR_adj_BMI | 0.92 (0.86-0.99) | 1.98×10^-2^* |  | 0.91 (0.84-0.98) | 1.92×10^-2^* |  | 0.96 (0.88-1.05) | 0.35 |
| childhood BMI | 0.80 (0.74-0.87) | 1.24×10^-7^* |  | 0.82 (0.75-0.90) | 3.40×10^-5^* |  | 0.74 (0.67-0.82) | 6.09×10^-9^* |
| Model 1: independent effect of adult BMI and childhood BMI on BC; Model 2: independent effect of adult WHR and childhood BMI on BC; Model 3: independent effect of adult WHR_adj_BMI and childhood BMI on BC. Asterisks (*) denote statistical significance survived false discovery rate (FDR) correction (*P*_FDR_ <0.05).  Abbreviations: BMI, body mass index; WHR, waist-to-hip ratio; WHR_adj_BMI, waist-to-hip ratio adjusted for body mass index; BC, breast cancer; ER, estrogen receptor; OR, odds ratio; 95%CI, 95% confidence interval. | | | | | | | | |


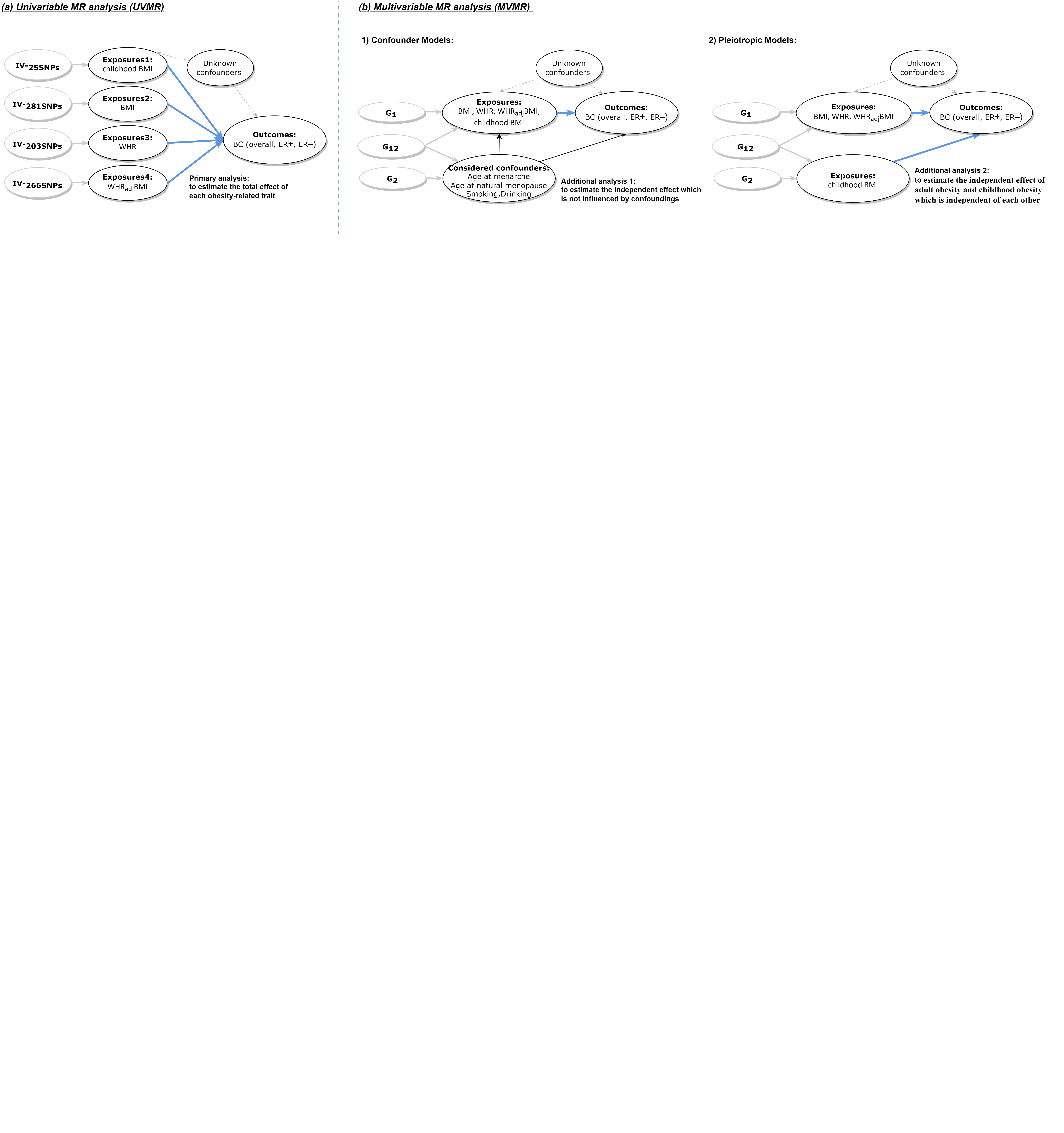


**Supplementary Figure 1.** Analytical schematic diagram of the comprehensive Mendelian randomization (MR) analysis implemented in this study: (a) Univariable MR analysis; (b) Multivariable MR analysis, including two assumed models: 1) Confounder models; 2) Pleiotropic models. The highlighted blue arrows on these graphs illustrate the causal effect of obesity-related traits on BC risk being estimated in MR analyses. G1, G2 and G12 are subsets of the full set of SNPs G that affect Trait 1, Trait 2 and both Traits, respectively.

Abbreviations: IV, instrumental variable; BMI, body mass index; WHR, waist-to-hip ratio; WHR_adj_BMI, waist-to-hip ratio adjusted for body mass index; BC, breast cancer; ER, estrogen receptor.

| **Supplementary Table 1. Characteristics of index SNPs associated with obesity-related traits and their effect sizes with outcomes^#^.** | | | | | | | | | | | | | | | | | |
| --- | --- | --- | --- | --- | --- | --- | --- | --- | --- | --- | --- | --- | --- | --- | --- | --- | --- |
| **SNP** | **Chr** | **Position** | **E**AF | **A1** | **A2** | **Exposure** | | | **Outcome:** BC overall | | | **Outcome:** ER**+** | | | **Outcome:** ER**–** | | |
|  |  |  |  |  |  | **beta** | **se** | ***P*-value** | **beta** | **se** | ***P*-value** | **beta** | **se** | ***P*-value** | **beta** | **se** | ***P*-value** |
| **BMI (female)** |  |  |  |  |  |  |  |  |  |  |  |  |  |  |  |  |  |
| rs10002111 | 4 | 67815504 | 0.22 | A | G | 0.017 | 0.003 | 9.26×10^-10^ | -0.016 | 0.007 | 2.51×10^-02^ | -0.020 | 0.009 | 2.76×10^-02^ | -0.010 | 0.014 | 0.470 |
| rs1003081 | 11 | 118913993 | 0.43 | T | C | 0.014 | 0.002 | 6.27×10^-11^ | -0.016 | 0.006 | 1.02×10^-02^ | -0.009 | 0.008 | 0.236 | -0.008 | 0.012 | 0.508 |
| rs1016287 | 2 | 59305625 | 0.30 | T | C | 0.022 | 0.002 | 4.39×10^-19^ | -0.003 | 0.007 | 0.608 | -0.003 | 0.008 | 0.679 | 0.005 | 0.013 | 0.669 |
| rs10179086 | 2 | 232744209 | 0.79 | T | C | -0.016 | 0.003 | 3.06×10^-09^ | 0.014 | 0.007 | 4.57×10^-02^ | 0.012 | 0.009 | 0.175 | -0.013 | 0.013 | 0.338 |
| rs10203386 | 2 | 25136866 | 0.45 | A | T | 0.034 | 0.002 | 2.52×10^-50^ | -0.036 | 0.006 | 1.02×10^-08^ | -0.036 | 0.008 | 5.20×10^-06^ | -0.060 | 0.012 | 6.59×10^-07^ |
| rs10237317 | 7 | 70045941 | 0.59 | A | G | -0.014 | 0.002 | 6.82×10^-10^ | 0.001 | 0.006 | 0.843 | -0.002 | 0.008 | 0.756 | 0.008 | 0.012 | 0.477 |
| rs1025395 | 8 | 8836603 | 0.44 | C | G | 0.017 | 0.002 | 8.42×10^-13^ | -0.011 | 0.007 | 0.123 | -0.016 | 0.009 | 0.078 | -0.018 | 0.013 | 0.164 |
| rs10440098 | 3 | 20578998 | 0.60 | T | C | 0.015 | 0.002 | 8.77×10^-10^ | -0.007 | 0.006 | 0.284 | -0.004 | 0.008 | 0.608 | 0.013 | 0.012 | 0.267 |
| rs1048932 | 11 | 115044850 | 0.44 | A | C | -0.018 | 0.002 | 2.52×10^-16^ | 0.008 | 0.006 | 0.184 | 0.005 | 0.008 | 0.493 | -0.003 | 0.012 | 0.816 |
| rs10499694 | 7 | 50614173 | 0.49 | A | G | 0.014 | 0.002 | 3.99×10^-10^ | 0.003 | 0.006 | 0.658 | 0.003 | 0.008 | 0.673 | 0.001 | 0.012 | 0.968 |
| rs10513801 | 3 | 185822353 | 0.88 | T | G | 0.035 | 0.003 | 1.26×10^-27^ | -0.009 | 0.009 | 0.330 | -0.002 | 0.011 | 0.831 | 0.003 | 0.017 | 0.842 |
| rs1075901 | 17 | 15943910 | 0.45 | T | C | -0.014 | 0.002 | 4.36×10^-11^ | -0.001 | 0.006 | 0.905 | -0.005 | 0.008 | 0.535 | 0.009 | 0.011 | 0.454 |
| rs10761785 | 10 | 65318766 | 0.51 | T | G | -0.017 | 0.002 | 4.17×10^-15^ | -0.025 | 0.006 | 2.93×10^-05^ | -0.030 | 0.007 | 6.08×10^-05^ | -0.005 | 0.011 | 0.670 |
| rs10783779 | 12 | 56491880 | 0.60 | T | G | 0.014 | 0.002 | 2.06×10^-09^ | 0.015 | 0.006 | 1.92×10^-02^ | 0.020 | 0.008 | 1.25×10^-02^ | 0.009 | 0.012 | 0.449 |
| rs10797987 | 1 | 184651822 | 0.51 | T | C | -0.016 | 0.003 | 2.05×10^-09^ | 0.000 | 0.006 | 0.998 | 0.002 | 0.007 | 0.760 | 0.002 | 0.011 | 0.880 |
| rs10823893 | 10 | 53677313 | 0.41 | A | G | 0.018 | 0.003 | 5.22×10^-11^ | -0.018 | 0.006 | 3.17×10^-03^ | -0.019 | 0.008 | 1.18×10^-02^ | -0.008 | 0.012 | 0.493 |
| rs10824347 | 10 | 77645229 | 0.77 | A | T | 0.018 | 0.003 | 5.47×10^-11^ | 0.006 | 0.007 | 0.407 | 0.013 | 0.009 | 0.162 | -0.022 | 0.013 | 0.096 |
| rs10871589 | 18 | 63285783 | 0.68 | A | G | -0.018 | 0.003 | 1.89×10^-10^ | 0.004 | 0.006 | 0.562 | 0.009 | 0.008 | 0.266 | 0.019 | 0.012 | 0.125 |
| rs10886017 | 10 | 118672531 | 0.24 | A | C | 0.017 | 0.003 | 3.89×10^-11^ | 0.001 | 0.007 | 0.846 | -0.002 | 0.009 | 0.814 | 0.012 | 0.014 | 0.395 |
| rs10892873 | 11 | 122535333 | 0.36 | C | G | 0.014 | 0.002 | 2.01×10^-09^ | 0.010 | 0.007 | 0.140 | 0.016 | 0.008 | 0.056 | -0.013 | 0.012 | 0.311 |
| rs10920678 | 1 | 190239907 | 0.42 | A | G | 0.016 | 0.002 | 7.46×10^-14^ | -0.003 | 0.006 | 0.596 | -0.006 | 0.008 | 0.439 | -0.008 | 0.012 | 0.500 |
| rs10930502 | 2 | 172890588 | 0.69 | A | G | 0.014 | 0.002 | 1.43×10^-09^ | -0.008 | 0.007 | 0.233 | -0.004 | 0.008 | 0.651 | -0.033 | 0.012 | 7.50×10^-03^ |
| rs10938397 | 4 | 45182527 | 0.57 | A | G | -0.032 | 0.002 | 1.66×10^-45^ | 0.015 | 0.006 | 1.32×10^-02^ | 0.019 | 0.007 | 9.90×10^-03^ | 0.024 | 0.011 | 3.70×10^-02^ |
| rs10942267 | 5 | 80841914 | 0.69 | A | G | 0.016 | 0.003 | 5.85×10^-11^ | 0.019 | 0.007 | 2.89×10^-03^ | 0.018 | 0.008 | 2.55×10^-02^ | 0.014 | 0.012 | 0.273 |
| rs10950289 | 7 | 71429308 | 0.84 | A | G | 0.021 | 0.004 | 4.60×10^-09^ | 0.004 | 0.009 | 0.601 | 0.004 | 0.011 | 0.733 | 0.000 | 0.016 | 0.997 |
| rs11038428 | 11 | 45404318 | 0.28 | T | G | -0.015 | 0.002 | 8.80×10^-10^ | 0.007 | 0.007 | 0.286 | 0.014 | 0.008 | 0.096 | -0.005 | 0.013 | 0.723 |
| rs11073383 | 15 | 95274349 | 0.48 | A | G | -0.016 | 0.002 | 3.26×10^-12^ | 0.003 | 0.006 | 0.632 | 0.010 | 0.007 | 0.194 | -0.007 | 0.011 | 0.558 |
| rs11074446 | 16 | 20255123 | 0.85 | T | C | 0.021 | 0.003 | 1.25×10^-10^ | -0.005 | 0.009 | 0.606 | 0.002 | 0.011 | 0.861 | -0.033 | 0.017 | 4.84×10^-02^ |
| rs11181001 | 12 | 41948196 | 0.48 | A | G | 0.013 | 0.002 | 1.56×10^-09^ | 0.009 | 0.006 | 0.133 | 0.009 | 0.008 | 0.236 | 0.011 | 0.012 | 0.342 |
| rs11218510 | 11 | 121922587 | 0.39 | A | G | -0.017 | 0.003 | 2.64×10^-10^ | 0.008 | 0.006 | 0.194 | 0.012 | 0.008 | 0.134 | -0.017 | 0.012 | 0.155 |
| rs1121980 | 16 | 53809247 | 0.44 | A | G | 0.067 | 0.002 | 1.00×10^-200^ | -0.055 | 0.006 | 1.00×10^-19^ | -0.054 | 0.007 | 5.18×10^-13^ | -0.076 | 0.011 | 3.38×10^-11^ |
| rs112566467 | 1 | 39562627 | 0.21 | T | C | 0.020 | 0.003 | 2.72×10^-10^ | 0.008 | 0.007 | 0.305 | 0.004 | 0.009 | 0.648 | 0.020 | 0.014 | 0.162 |
| rs113706999 | 3 | 44159156 | 0.02 | A | T | 0.055 | 0.009 | 1.56×10^-09^ | -0.019 | 0.033 | 0.562 | 0.021 | 0.041 | 0.614 | -0.200 | 0.070 | 4.22×10^-03^ |
| rs11611246 | 12 | 939480 | 0.20 | T | G | 0.021 | 0.003 | 1.27×10^-14^ | 0.010 | 0.007 | 0.159 | 0.017 | 0.009 | 0.073 | -0.005 | 0.014 | 0.749 |
| rs11642612 | 16 | 30030195 | 0.59 | A | C | -0.023 | 0.002 | 1.08×10^-22^ | -0.004 | 0.006 | 0.535 | 0.003 | 0.008 | 0.706 | -0.017 | 0.012 | 0.140 |
| rs11649864 | 17 | 56093061 | 0.09 | A | G | 0.025 | 0.004 | 1.78×10^-09^ | -0.001 | 0.011 | 0.946 | 0.016 | 0.014 | 0.249 | -0.019 | 0.022 | 0.375 |
| rs11660335 | 18 | 22154235 | 0.82 | T | C | 0.019 | 0.003 | 1.20×10^-10^ | 0.014 | 0.008 | 0.090 | 0.014 | 0.010 | 0.173 | 0.039 | 0.015 | 1.22×10^-02^ |
| rs11672660 | 19 | 46180184 | 0.19 | T | C | -0.032 | 0.003 | 5.93×10^-31^ | 0.045 | 0.008 | 5.52×10^-09^ | 0.056 | 0.010 | 5.13×10^-09^ | 0.027 | 0.015 | 0.059 |
| rs11677607 | 2 | 100751150 | 0.25 | T | C | -0.024 | 0.003 | 2.87×10^-19^ | 0.012 | 0.007 | 0.085 | 0.013 | 0.009 | 0.137 | 0.017 | 0.013 | 0.208 |
| rs11692326 | 2 | 208263279 | 0.23 | T | C | 0.016 | 0.003 | 2.04×10^-09^ | 0.002 | 0.007 | 0.750 | 0.008 | 0.009 | 0.372 | -0.007 | 0.014 | 0.603 |
| rs11727273 | 4 | 55509748 | 0.55 | T | C | -0.017 | 0.003 | 1.28×10^-10^ | 0.002 | 0.006 | 0.789 | 0.010 | 0.008 | 0.221 | -0.015 | 0.012 | 0.210 |
| rs11802147 | 1 | 108001307 | 0.70 | A | G | 0.015 | 0.003 | 1.38×10^-09^ | 0.004 | 0.007 | 0.590 | 0.002 | 0.008 | 0.786 | -0.002 | 0.012 | 0.903 |
| rs11824092 | 11 | 13346294 | 0.35 | T | C | -0.016 | 0.002 | 1.72×10^-11^ | -0.004 | 0.006 | 0.569 | -0.005 | 0.008 | 0.555 | 0.008 | 0.012 | 0.500 |
| rs11856579 | 15 | 78012688 | 0.26 | A | G | -0.017 | 0.003 | 1.14×10^-10^ | -0.005 | 0.007 | 0.455 | -0.002 | 0.009 | 0.832 | -0.010 | 0.013 | 0.463 |
| rs11866219 | 16 | 69549749 | 0.42 | A | C | 0.021 | 0.002 | 2.34×10^-19^ | 0.016 | 0.006 | 1.40×10^-02^ | 0.011 | 0.008 | 0.154 | 0.033 | 0.012 | 5.40×10^-03^ |
| rs11924032 | 3 | 170735099 | 0.25 | A | G | 0.015 | 0.003 | 4.45×10^-09^ | 0.012 | 0.007 | 0.104 | -0.004 | 0.009 | 0.632 | 0.035 | 0.014 | 8.96×10^-03^ |
| rs12033257 | 1 | 112318484 | 0.64 | A | G | 0.017 | 0.002 | 5.57×10^-12^ | -0.006 | 0.007 | 0.348 | -0.010 | 0.008 | 0.224 | 0.014 | 0.012 | 0.262 |
| rs12042959 | 1 | 243533273 | 0.86 | A | G | 0.019 | 0.003 | 2.33×10^-09^ | 0.020 | 0.009 | 1.94×10^-02^ | 0.013 | 0.011 | 0.223 | 0.031 | 0.016 | 0.055 |
| rs12098284 | 10 | 76047464 | 0.12 | T | C | 0.021 | 0.004 | 3.72×10^-09^ | -0.011 | 0.009 | 0.228 | -0.020 | 0.012 | 0.092 | -0.016 | 0.018 | 0.365 |
| rs12121950 | 1 | 49710264 | 0.34 | T | G | 0.019 | 0.003 | 2.03×10^-14^ | -0.015 | 0.007 | 2.24×10^-02^ | -0.023 | 0.008 | 5.06×10^-03^ | -0.033 | 0.013 | 8.83×10^-03^ |
| rs12140153 | 1 | 62579891 | 0.09 | T | G | -0.033 | 0.005 | 2.87×10^-12^ | 0.014 | 0.011 | 0.205 | 0.029 | 0.014 | 3.91×10^-02^ | -0.024 | 0.022 | 0.257 |
| rs12328675 | 2 | 165540800 | 0.87 | T | C | -0.020 | 0.003 | 3.98×10^-09^ | -0.012 | 0.009 | 0.170 | -0.015 | 0.011 | 0.181 | 0.023 | 0.017 | 0.177 |
| rs12364470 | 11 | 134601012 | 0.86 | T | G | -0.020 | 0.003 | 2.54×10^-11^ | 0.011 | 0.009 | 0.236 | 0.006 | 0.012 | 0.584 | 0.007 | 0.017 | 0.683 |
| rs12429545 | 13 | 54102206 | 0.12 | A | G | 0.029 | 0.003 | 1.09×10^-18^ | 0.005 | 0.009 | 0.605 | 0.010 | 0.012 | 0.394 | 0.036 | 0.018 | 4.29×10^-02^ |
| rs12431244 | 13 | 58630651 | 0.25 | T | G | -0.022 | 0.003 | 1.22×10^-15^ | -0.005 | 0.007 | 0.444 | -0.015 | 0.009 | 0.080 | -0.002 | 0.013 | 0.859 |
| rs12446632 | 16 | 19935389 | 0.14 | A | G | -0.038 | 0.003 | 3.01×10^-32^ | 0.013 | 0.009 | 0.123 | 0.002 | 0.011 | 0.884 | 0.037 | 0.016 | 2.30×10^-02^ |
| rs12449442 | 17 | 65947640 | 0.22 | A | G | 0.016 | 0.003 | 2.22×10^-09^ | 0.001 | 0.007 | 0.843 | -0.004 | 0.009 | 0.702 | 0.023 | 0.014 | 0.097 |
| rs12462975 | 19 | 30272202 | 0.32 | A | G | 0.020 | 0.003 | 2.63×10^-16^ | 0.020 | 0.006 | 2.30×10^-03^ | 0.017 | 0.008 | 3.03×10^-02^ | 0.059 | 0.012 | 7.13×10^-07^ |
| rs12468070 | 2 | 6159981 | 0.26 | T | C | -0.015 | 0.003 | 1.96×10^-09^ | 0.011 | 0.007 | 0.126 | 0.016 | 0.009 | 0.063 | -0.005 | 0.013 | 0.731 |
| rs12484438 | 22 | 40558064 | 0.65 | T | C | 0.015 | 0.002 | 3.58×10^-10^ | -0.038 | 0.006 | 2.41×10^-09^ | -0.029 | 0.008 | 1.67×10^-04^ | -0.047 | 0.012 | 5.91×10^-05^ |
| rs1249150 | 10 | 79980507 | 0.71 | A | G | 0.016 | 0.003 | 4.40×10^-10^ | 0.014 | 0.007 | 4.77×10^-02^ | 0.015 | 0.009 | 0.093 | 0.010 | 0.014 | 0.455 |
| rs12564992 | 1 | 174478100 | 0.89 | A | G | -0.021 | 0.004 | 2.37×10^-09^ | -0.002 | 0.009 | 0.796 | -0.004 | 0.012 | 0.749 | -0.007 | 0.018 | 0.695 |
| rs12593036 | 15 | 81058652 | 0.69 | A | G | 0.015 | 0.003 | 6.65×10^-10^ | -0.001 | 0.007 | 0.905 | -0.007 | 0.008 | 0.427 | 0.002 | 0.013 | 0.876 |
| rs12645001 | 4 | 96018337 | 0.36 | A | G | -0.014 | 0.002 | 4.11×10^-09^ | -0.009 | 0.006 | 0.174 | -0.012 | 0.008 | 0.123 | -0.010 | 0.012 | 0.421 |
| rs12681792 | 8 | 62054463 | 0.20 | A | C | 0.018 | 0.003 | 2.61×10^-10^ | 0.010 | 0.008 | 0.196 | 0.015 | 0.010 | 0.143 | -0.024 | 0.015 | 0.124 |
| rs12692596 | 2 | 161265910 | 0.36 | T | C | 0.015 | 0.002 | 9.74×10^-11^ | 0.012 | 0.006 | 0.058 | 0.008 | 0.008 | 0.292 | 0.015 | 0.012 | 0.202 |
| rs12705916 | 7 | 113492834 | 0.62 | T | C | -0.017 | 0.002 | 1.62×10^-12^ | -0.015 | 0.006 | 2.18×10^-02^ | -0.010 | 0.008 | 0.197 | -0.018 | 0.012 | 0.134 |
| rs12714199 | 2 | 86812549 | 0.61 | T | C | -0.015 | 0.002 | 5.12×10^-10^ | 0.007 | 0.006 | 0.259 | 0.005 | 0.008 | 0.514 | 0.014 | 0.012 | 0.253 |
| rs12729914 | 1 | 77980235 | 0.83 | T | C | -0.024 | 0.003 | 8.57×10^-18^ | -0.004 | 0.008 | 0.602 | -0.007 | 0.010 | 0.492 | -0.012 | 0.015 | 0.428 |
| rs1285997 | 14 | 91513029 | 0.29 | C | G | -0.015 | 0.003 | 3.18×10^-09^ | 0.001 | 0.007 | 0.923 | 0.009 | 0.008 | 0.287 | -0.028 | 0.013 | 2.80×10^-02^ |
| rs1289736 | 3 | 107950892 | 0.54 | T | C | 0.014 | 0.002 | 1.43×10^-09^ | -0.005 | 0.006 | 0.396 | -0.004 | 0.008 | 0.576 | -0.019 | 0.012 | 0.107 |
| rs12914489 | 15 | 74187937 | 0.08 | A | G | 0.023 | 0.004 | 1.64×10^-10^ | 0.022 | 0.010 | 2.72×10^-02^ | 0.022 | 0.012 | 0.075 | -0.003 | 0.019 | 0.861 |
| rs12984770 | 19 | 18467398 | 0.73 | A | C | 0.020 | 0.003 | 3.92×10^-11^ | -0.003 | 0.007 | 0.654 | -0.006 | 0.009 | 0.471 | -0.002 | 0.013 | 0.905 |
| rs13002158 | 2 | 144021712 | 0.81 | A | G | -0.021 | 0.003 | 1.15×10^-12^ | 0.016 | 0.008 | 4.87×10^-02^ | 0.014 | 0.010 | 0.159 | 0.029 | 0.015 | 0.061 |
| rs13098327 | 3 | 85820181 | 0.19 | A | G | 0.023 | 0.003 | 5.13×10^-17^ | -0.010 | 0.008 | 0.185 | -0.006 | 0.009 | 0.549 | -0.012 | 0.014 | 0.387 |
| rs13107325 | 4 | 103188709 | 0.08 | T | C | 0.045 | 0.004 | 9.33×10^-25^ | 0.019 | 0.012 | 0.119 | 0.020 | 0.015 | 0.178 | -0.006 | 0.023 | 0.776 |
| rs13110266 | 4 | 162129844 | 0.40 | A | G | -0.014 | 0.002 | 1.55×10^-10^ | -0.002 | 0.006 | 0.774 | -0.001 | 0.008 | 0.858 | -0.010 | 0.012 | 0.406 |
| rs13174863 | 5 | 139080745 | 0.85 | A | G | -0.027 | 0.003 | 4.53×10^-18^ | -0.015 | 0.009 | 0.114 | -0.031 | 0.012 | 8.42×10^-03^ | 0.009 | 0.018 | 0.627 |
| rs13191362 | 6 | 163033350 | 0.86 | A | G | 0.021 | 0.003 | 6.40×10^-10^ | 0.010 | 0.009 | 0.306 | 0.000 | 0.012 | 0.997 | 0.037 | 0.018 | 4.05×10^-02^ |
| rs1320251 | 17 | 21264396 | 0.46 | T | C | -0.017 | 0.002 | 2.12×10^-13^ | -0.002 | 0.006 | 0.805 | 0.003 | 0.008 | 0.702 | 0.004 | 0.012 | 0.727 |
| rs1329733 | 9 | 92208227 | 0.53 | A | G | -0.016 | 0.002 | 1.93×10^-12^ | 0.006 | 0.006 | 0.308 | 0.002 | 0.007 | 0.834 | 0.041 | 0.011 | 2.84×10^-04^ |
| rs13330107 | 16 | 76878862 | 0.35 | A | G | 0.014 | 0.002 | 4.53×10^-09^ | 0.008 | 0.006 | 0.238 | 0.004 | 0.008 | 0.661 | 0.014 | 0.012 | 0.253 |
| rs13417156 | 2 | 62848319 | 0.58 | T | C | -0.017 | 0.002 | 4.08×10^-14^ | -0.006 | 0.006 | 0.357 | -0.014 | 0.008 | 0.065 | 0.009 | 0.011 | 0.429 |
| rs1409818 | 20 | 21381121 | 0.11 | T | C | 0.025 | 0.004 | 1.58×10^-11^ | -0.018 | 0.010 | 0.082 | -0.023 | 0.013 | 0.065 | -0.014 | 0.019 | 0.465 |
| rs1417665 | 6 | 104816944 | 0.80 | T | C | 0.020 | 0.003 | 5.68×10^-12^ | -0.021 | 0.008 | 8.49×10^-03^ | -0.025 | 0.010 | 1.24×10^-02^ | -0.017 | 0.015 | 0.254 |
| rs1452075 | 3 | 62481063 | 0.73 | T | C | 0.016 | 0.003 | 1.47×10^-10^ | 0.012 | 0.007 | 0.083 | 0.017 | 0.009 | 0.057 | 0.016 | 0.013 | 0.225 |
| rs1460676 | 2 | 164567689 | 0.82 | T | C | -0.018 | 0.003 | 1.05×10^-09^ | 0.002 | 0.008 | 0.763 | 0.001 | 0.010 | 0.887 | 0.004 | 0.015 | 0.798 |
| rs1470545 | 2 | 205365851 | 0.04 | T | C | 0.039 | 0.006 | 1.25×10^-11^ | -0.009 | 0.016 | 0.555 | -0.012 | 0.020 | 0.549 | -0.028 | 0.030 | 0.356 |
| rs1477887 | 4 | 18514827 | 0.44 | A | G | -0.016 | 0.002 | 1.69×10^-11^ | 0.004 | 0.006 | 0.498 | -0.001 | 0.007 | 0.907 | 0.001 | 0.011 | 0.949 |
| rs1518159 | 18 | 40760146 | 0.25 | T | C | -0.019 | 0.003 | 6.66×10^-13^ | -0.005 | 0.007 | 0.457 | -0.013 | 0.009 | 0.135 | 0.009 | 0.013 | 0.479 |
| rs1524445 | 7 | 113035833 | 0.42 | T | C | -0.020 | 0.003 | 9.80×10^-14^ | -0.002 | 0.006 | 0.764 | 0.000 | 0.008 | 0.994 | 0.007 | 0.012 | 0.577 |
| rs1561589 | 10 | 126695673 | 0.36 | A | G | 0.016 | 0.002 | 4.24×10^-12^ | -0.005 | 0.006 | 0.425 | 0.002 | 0.008 | 0.823 | -0.021 | 0.012 | 0.069 |
| rs1569979 | 14 | 29681294 | 0.77 | A | G | 0.023 | 0.003 | 1.57×10^-17^ | -0.002 | 0.007 | 0.800 | -0.002 | 0.009 | 0.794 | -0.009 | 0.014 | 0.520 |
| rs1579557 | 6 | 40371918 | 0.29 | T | C | 0.021 | 0.003 | 3.09×10^-17^ | -0.003 | 0.007 | 0.640 | -0.001 | 0.009 | 0.913 | 0.003 | 0.014 | 0.819 |
| rs1580099 | 3 | 94003603 | 0.58 | A | C | -0.018 | 0.002 | 6.70×10^-16^ | 0.001 | 0.006 | 0.834 | 0.002 | 0.007 | 0.838 | 0.017 | 0.011 | 0.145 |
| rs16851483 | 3 | 141275436 | 0.07 | T | G | 0.036 | 0.005 | 2.28×10^-15^ | 0.006 | 0.012 | 0.631 | 0.021 | 0.015 | 0.164 | 0.018 | 0.024 | 0.444 |
| rs16912921 | 9 | 28413461 | 0.31 | A | C | 0.025 | 0.002 | 1.56×10^-26^ | -0.001 | 0.006 | 0.863 | 0.000 | 0.008 | 0.990 | -0.008 | 0.012 | 0.518 |
| rs16926778 | 12 | 24002125 | 0.08 | C | G | 0.026 | 0.004 | 1.41×10^-09^ | 0.006 | 0.011 | 0.581 | 0.005 | 0.014 | 0.734 | 0.003 | 0.022 | 0.881 |
| rs16975921 | 18 | 39914870 | 0.69 | A | T | -0.016 | 0.003 | 3.87×10^-11^ | 0.009 | 0.006 | 0.175 | 0.013 | 0.008 | 0.104 | 0.026 | 0.012 | 3.07×10^-02^ |
| rs17405819 | 8 | 76806584 | 0.68 | T | C | 0.022 | 0.002 | 7.81×10^-20^ | -0.013 | 0.007 | 4.05×10^-02^ | -0.014 | 0.008 | 0.079 | -0.009 | 0.012 | 0.473 |
| rs17522122 | 14 | 33302882 | 0.47 | T | G | 0.017 | 0.002 | 6.46×10^-14^ | -0.010 | 0.006 | 0.112 | -0.005 | 0.008 | 0.500 | 0.007 | 0.012 | 0.529 |
| rs17806224 | 20 | 51065854 | 0.18 | A | G | -0.029 | 0.003 | 3.52×10^-22^ | -0.019 | 0.008 | 1.76×10^-02^ | -0.025 | 0.010 | 1.51×10^-02^ | -0.037 | 0.016 | 1.95×10^-02^ |
| rs1808579 | 18 | 21104888 | 0.48 | T | C | -0.020 | 0.002 | 9.44×10^-20^ | -0.009 | 0.006 | 0.142 | -0.015 | 0.008 | 4.87×10^-02^ | -0.013 | 0.011 | 0.257 |
| rs185581 | 2 | 59880605 | 0.21 | A | C | 0.017 | 0.003 | 4.19×10^-09^ | 0.012 | 0.008 | 0.134 | 0.008 | 0.010 | 0.426 | 0.007 | 0.015 | 0.647 |
| rs1861412 | 2 | 58893065 | 0.42 | A | G | -0.022 | 0.002 | 9.13×10^-21^ | 0.004 | 0.006 | 0.520 | -0.002 | 0.007 | 0.822 | 0.000 | 0.011 | 0.989 |
| rs1884389 | 20 | 1410582 | 0.44 | T | C | -0.014 | 0.002 | 1.20×10^-09^ | 0.010 | 0.006 | 0.094 | 0.009 | 0.007 | 0.244 | -0.002 | 0.011 | 0.853 |
| rs1884897 | 20 | 6612832 | 0.37 | A | G | -0.017 | 0.002 | 9.06×10^-14^ | 0.005 | 0.006 | 0.427 | 0.005 | 0.008 | 0.561 | 0.018 | 0.012 | 0.137 |
| rs1895407 | 5 | 63021638 | 0.52 | T | C | -0.016 | 0.002 | 6.61×10^-12^ | -0.008 | 0.006 | 0.184 | -0.012 | 0.007 | 0.098 | -0.009 | 0.011 | 0.412 |
| rs1895941 | 12 | 108332994 | 0.26 | A | G | -0.018 | 0.003 | 8.19×10^-12^ | 0.002 | 0.008 | 0.826 | 0.006 | 0.010 | 0.534 | -0.009 | 0.015 | 0.564 |
| rs1916801 | 3 | 61187046 | 0.62 | A | T | 0.017 | 0.002 | 1.75×10^-14^ | 0.003 | 0.006 | 0.579 | 0.001 | 0.008 | 0.924 | -0.005 | 0.012 | 0.685 |
| rs1927790 | 13 | 96922191 | 0.61 | T | C | -0.014 | 0.002 | 1.27×10^-09^ | 0.002 | 0.006 | 0.782 | -0.002 | 0.008 | 0.759 | -0.005 | 0.012 | 0.678 |
| rs1928295 | 9 | 120378483 | 0.57 | T | C | 0.016 | 0.002 | 2.85×10^-13^ | 0.002 | 0.006 | 0.754 | -0.002 | 0.008 | 0.842 | 0.004 | 0.011 | 0.710 |
| rs1932133 | 9 | 29750167 | 0.59 | A | C | 0.014 | 0.002 | 2.67×10^-10^ | 0.004 | 0.006 | 0.470 | -0.005 | 0.007 | 0.484 | 0.006 | 0.011 | 0.620 |
| rs1933440 | 13 | 28676971 | 0.84 | A | C | -0.022 | 0.004 | 5.38×10^-10^ | 0.006 | 0.008 | 0.456 | 0.009 | 0.010 | 0.381 | -0.021 | 0.015 | 0.180 |
| rs1967772 | 13 | 28036062 | 0.27 | A | G | -0.017 | 0.003 | 1.12×10^-10^ | 0.009 | 0.007 | 0.171 | 0.002 | 0.008 | 0.861 | 0.021 | 0.013 | 0.100 |
| rs198665 | 6 | 143154886 | 0.39 | A | C | 0.013 | 0.002 | 4.42×10^-09^ | -0.003 | 0.006 | 0.605 | -0.005 | 0.008 | 0.567 | 0.006 | 0.012 | 0.637 |
| rs2012502 | 16 | 81728081 | 0.38 | A | C | 0.015 | 0.002 | 2.97×10^-11^ | -0.008 | 0.006 | 0.215 | -0.006 | 0.008 | 0.469 | -0.016 | 0.012 | 0.208 |
| rs2053682 | 5 | 170599327 | 0.68 | A | C | 0.018 | 0.003 | 1.89×10^-13^ | -0.001 | 0.007 | 0.896 | 0.002 | 0.008 | 0.829 | -0.001 | 0.012 | 0.970 |
| rs2075466 | 16 | 4872970 | 0.27 | C | G | 0.016 | 0.003 | 4.29×10^-10^ | -0.011 | 0.007 | 0.140 | -0.003 | 0.009 | 0.775 | 0.019 | 0.014 | 0.183 |
| rs2105054 | 9 | 11425445 | 0.44 | T | C | -0.014 | 0.002 | 1.17×10^-09^ | 0.004 | 0.006 | 0.497 | 0.014 | 0.008 | 0.074 | -0.013 | 0.012 | 0.286 |
| rs2112347 | 5 | 75015242 | 0.63 | T | G | 0.031 | 0.002 | 2.13×10^-42^ | -0.020 | 0.006 | 1.36×10^-03^ | -0.015 | 0.008 | 4.40×10^-02^ | -0.034 | 0.012 | 3.28×10^-03^ |
| rs2119137 | 2 | 175017714 | 0.66 | A | G | -0.014 | 0.002 | 3.26×10^-09^ | 0.001 | 0.006 | 0.937 | 0.001 | 0.008 | 0.870 | 0.006 | 0.012 | 0.614 |
| rs2153615 | 6 | 46375326 | 0.45 | A | G | -0.016 | 0.002 | 2.42×10^-12^ | -0.005 | 0.006 | 0.468 | -0.011 | 0.008 | 0.167 | -0.010 | 0.012 | 0.425 |
| rs2187449 | 11 | 133712682 | 0.77 | A | G | 0.016 | 0.003 | 3.66×10^-09^ | -0.014 | 0.008 | 0.072 | -0.012 | 0.009 | 0.191 | -0.018 | 0.014 | 0.204 |
| rs2212450 | 11 | 112826867 | 0.57 | T | C | 0.015 | 0.002 | 3.70×10^-10^ | 0.000 | 0.006 | 0.946 | 0.000 | 0.008 | 0.981 | 0.016 | 0.012 | 0.191 |
| rs2228213 | 6 | 12124855 | 0.34 | A | G | -0.015 | 0.002 | 9.21×10^-11^ | 0.001 | 0.006 | 0.919 | -0.003 | 0.008 | 0.678 | 0.009 | 0.012 | 0.453 |
| rs2274319 | 1 | 156450873 | 0.35 | T | C | -0.014 | 0.002 | 3.91×10^-09^ | 0.006 | 0.006 | 0.339 | 0.007 | 0.008 | 0.354 | 0.003 | 0.012 | 0.823 |
| rs2274782 | 9 | 127084074 | 0.36 | T | C | 0.015 | 0.002 | 3.38×10^-10^ | -0.018 | 0.006 | 5.65×10^-03^ | -0.014 | 0.008 | 0.086 | -0.032 | 0.012 | 6.92×10^-03^ |
| rs2299383 | 7 | 103418846 | 0.40 | T | C | 0.018 | 0.002 | 2.73×10^-16^ | -0.013 | 0.006 | 3.07×10^-02^ | -0.008 | 0.008 | 0.302 | -0.018 | 0.012 | 0.132 |
| rs2306593 | 17 | 34866546 | 0.49 | T | C | -0.016 | 0.002 | 7.58×10^-13^ | -0.007 | 0.007 | 0.316 | -0.011 | 0.008 | 0.187 | 0.002 | 0.013 | 0.863 |
| rs2357760 | 6 | 120213880 | 0.67 | A | G | 0.016 | 0.002 | 1.09×10^-11^ | -0.007 | 0.006 | 0.260 | -0.002 | 0.008 | 0.833 | -0.019 | 0.012 | 0.110 |
| rs2381977 | 2 | 147847890 | 0.61 | T | C | -0.016 | 0.003 | 3.07×10^-09^ | -0.014 | 0.007 | 3.07×10^-02^ | -0.017 | 0.008 | 3.60×10^-02^ | 0.004 | 0.013 | 0.748 |
| rs2452141 | 11 | 29230734 | 0.36 | A | T | -0.014 | 0.002 | 6.86×10^-10^ | 0.002 | 0.006 | 0.794 | 0.000 | 0.008 | 0.966 | 0.016 | 0.012 | 0.165 |
| rs247975 | 3 | 173107443 | 0.47 | T | C | -0.018 | 0.002 | 3.62×10^-15^ | 0.002 | 0.007 | 0.725 | -0.006 | 0.009 | 0.514 | 0.006 | 0.013 | 0.640 |
| rs249612 | 5 | 66200783 | 0.71 | T | C | 0.017 | 0.003 | 2.80×10^-09^ | -0.010 | 0.007 | 0.146 | -0.015 | 0.009 | 0.076 | -0.012 | 0.013 | 0.364 |
| rs2528531 | 7 | 93236510 | 0.65 | A | C | -0.015 | 0.002 | 1.35×10^-09^ | 0.007 | 0.006 | 0.287 | 0.005 | 0.008 | 0.490 | 0.017 | 0.012 | 0.158 |
| rs2568958 | 1 | 72765116 | 0.62 | A | G | 0.023 | 0.002 | 1.32×10^-24^ | -0.014 | 0.006 | 2.03×10^-02^ | -0.019 | 0.008 | 1.25×10^-02^ | -0.016 | 0.012 | 0.169 |
| rs2605603 | 11 | 93221105 | 0.48 | A | G | -0.013 | 0.002 | 1.40×10^-09^ | 0.000 | 0.006 | 0.991 | -0.007 | 0.008 | 0.379 | 0.014 | 0.011 | 0.222 |
| rs2619976 | 17 | 71754545 | 0.40 | T | C | 0.015 | 0.002 | 6.17×10^-10^ | 0.006 | 0.007 | 0.400 | 0.002 | 0.008 | 0.819 | 0.009 | 0.012 | 0.460 |
| rs264962 | 2 | 104308545 | 0.54 | C | G | 0.014 | 0.002 | 1.21×10^-09^ | 0.015 | 0.006 | 1.30×10^-02^ | 0.009 | 0.007 | 0.254 | 0.018 | 0.011 | 0.117 |
| rs2721965 | 8 | 116662038 | 0.67 | A | C | 0.019 | 0.002 | 2.10×10^-14^ | 0.020 | 0.006 | 1.37×10^-03^ | 0.031 | 0.008 | 7.00×10^-05^ | -0.001 | 0.012 | 0.953 |
| rs273504 | 19 | 18215247 | 0.58 | A | G | -0.015 | 0.002 | 1.27×10^-10^ | -0.017 | 0.006 | 6.80×10^-03^ | -0.016 | 0.008 | 3.44×10^-02^ | -0.019 | 0.012 | 0.095 |
| rs2744968 | 6 | 34584951 | 0.79 | A | C | -0.030 | 0.003 | 3.52×10^-27^ | 0.009 | 0.007 | 0.226 | 0.012 | 0.009 | 0.170 | 0.016 | 0.014 | 0.244 |
| rs2754084 | 14 | 25888677 | 0.52 | A | G | -0.017 | 0.002 | 2.29×10^-13^ | 0.013 | 0.006 | 3.60×10^-02^ | 0.013 | 0.008 | 0.088 | 0.014 | 0.012 | 0.225 |
| rs2803298 | 1 | 1846073 | 0.54 | T | G | 0.014 | 0.002 | 7.04×10^-10^ | -0.003 | 0.007 | 0.692 | -0.003 | 0.009 | 0.755 | -0.022 | 0.013 | 0.087 |
| rs2820295 | 1 | 201800868 | 0.33 | A | G | 0.021 | 0.002 | 8.03×10^-19^ | -0.006 | 0.006 | 0.340 | -0.010 | 0.008 | 0.212 | -0.002 | 0.012 | 0.870 |
| rs2837992 | 21 | 42620520 | 0.37 | T | C | -0.014 | 0.002 | 4.38×10^-09^ | 0.000 | 0.006 | 0.948 | 0.002 | 0.008 | 0.790 | 0.002 | 0.012 | 0.851 |
| rs284227 | 1 | 82379446 | 0.72 | T | C | -0.015 | 0.003 | 8.60×10^-10^ | -0.002 | 0.007 | 0.729 | -0.004 | 0.009 | 0.669 | 0.001 | 0.013 | 0.969 |
| rs28608644 | 4 | 20239663 | 0.85 | A | G | -0.024 | 0.004 | 7.63×10^-11^ | 0.003 | 0.009 | 0.757 | 0.001 | 0.011 | 0.957 | 0.027 | 0.016 | 0.098 |
| rs2861685 | 2 | 67837553 | 0.59 | T | C | 0.018 | 0.003 | 1.05×10^-11^ | 0.018 | 0.006 | 3.57×10^-03^ | 0.014 | 0.008 | 0.056 | 0.029 | 0.012 | 1.18×10^-02^ |
| rs2862961 | 11 | 43656535 | 0.70 | A | G | -0.024 | 0.003 | 5.30×10^-22^ | -0.007 | 0.007 | 0.316 | -0.009 | 0.008 | 0.291 | 0.005 | 0.012 | 0.662 |
| rs2916577 | 5 | 107316863 | 0.85 | A | G | 0.027 | 0.003 | 3.87×10^-18^ | -0.005 | 0.009 | 0.553 | -0.008 | 0.011 | 0.453 | -0.026 | 0.016 | 0.100 |
| rs2968487 | 1 | 96887370 | 0.28 | T | C | 0.017 | 0.003 | 2.44×10^-12^ | -0.011 | 0.007 | 0.119 | -0.021 | 0.009 | 1.69×10^-02^ | -0.002 | 0.013 | 0.907 |
| rs2968864 | 7 | 150622162 | 0.76 | T | C | 0.016 | 0.003 | 9.35×10^-10^ | -0.013 | 0.007 | 0.059 | -0.023 | 0.009 | 7.08×10^-03^ | -0.015 | 0.013 | 0.249 |
| rs29939 | 19 | 34310800 | 0.33 | A | G | -0.016 | 0.002 | 3.68×10^-11^ | 0.013 | 0.006 | 4.16×10^-02^ | 0.008 | 0.008 | 0.285 | -0.009 | 0.012 | 0.439 |
| rs3003612 | 9 | 130988616 | 0.56 | T | C | -0.015 | 0.002 | 1.31×10^-10^ | -0.003 | 0.006 | 0.644 | 0.000 | 0.008 | 1.000 | -0.011 | 0.012 | 0.335 |
| rs3026101 | 17 | 5280440 | 0.72 | T | C | -0.016 | 0.002 | 1.11×10^-11^ | -0.010 | 0.006 | 0.130 | 0.003 | 0.008 | 0.693 | -0.034 | 0.012 | 5.14×10^-03^ |
| rs308499 | 9 | 37348059 | 0.60 | C | G | 0.018 | 0.002 | 2.15×10^-14^ | -0.012 | 0.006 | 4.90×10^-02^ | -0.022 | 0.008 | 4.86×10^-03^ | -0.013 | 0.012 | 0.251 |
| rs3130048 | 6 | 31613739 | 0.73 | T | C | -0.020 | 0.002 | 7.83×10^-17^ | -0.002 | 0.007 | 0.821 | 0.008 | 0.009 | 0.372 | -0.002 | 0.013 | 0.859 |
| rs329120 | 5 | 133861756 | 0.41 | T | C | -0.015 | 0.002 | 7.61×10^-10^ | -0.004 | 0.006 | 0.473 | -0.003 | 0.008 | 0.660 | 0.013 | 0.011 | 0.261 |
| rs340025 | 15 | 60908307 | 0.41 | T | C | -0.014 | 0.002 | 3.82×10^-10^ | -0.006 | 0.006 | 0.364 | -0.004 | 0.008 | 0.641 | -0.009 | 0.012 | 0.467 |
| rs34292685 | 11 | 64049021 | 0.16 | T | C | -0.024 | 0.004 | 1.08×10^-11^ | 0.000 | 0.008 | 0.959 | -0.005 | 0.010 | 0.648 | -0.009 | 0.016 | 0.584 |
| rs34606703 | 2 | 47014522 | 0.36 | A | G | -0.019 | 0.003 | 1.18×10^-11^ | 0.001 | 0.006 | 0.841 | -0.008 | 0.008 | 0.291 | 0.003 | 0.012 | 0.821 |
| rs34811474 | 4 | 25408838 | 0.22 | A | G | -0.032 | 0.003 | 5.19×10^-24^ | -0.006 | 0.008 | 0.470 | 0.008 | 0.011 | 0.450 | -0.022 | 0.017 | 0.187 |
| rs34930419 | 10 | 33987329 | 0.97 | T | C | -0.046 | 0.008 | 1.37×10^-09^ | -0.010 | 0.018 | 0.560 | -0.002 | 0.022 | 0.926 | -0.043 | 0.034 | 0.210 |
| rs350818 | 19 | 4086807 | 0.23 | A | G | -0.017 | 0.003 | 1.09×10^-09^ | -0.012 | 0.007 | 0.121 | -0.014 | 0.009 | 0.130 | 0.013 | 0.014 | 0.357 |
| rs355754 | 3 | 154024165 | 0.60 | T | C | -0.014 | 0.002 | 4.26×10^-10^ | 0.000 | 0.006 | 0.963 | 0.006 | 0.008 | 0.478 | 0.009 | 0.012 | 0.448 |
| rs35679149 | 6 | 43604167 | 0.97 | A | G | 0.050 | 0.008 | 1.18×10^-09^ | 0.012 | 0.022 | 0.585 | 0.043 | 0.027 | 0.117 | 0.116 | 0.043 | 7.52×10^-03^ |
| rs3746038 | 19 | 1852494 | 0.22 | T | C | -0.018 | 0.003 | 1.11×10^-10^ | 0.001 | 0.007 | 0.891 | -0.005 | 0.009 | 0.568 | 0.016 | 0.014 | 0.246 |
| rs3774573 | 3 | 53799960 | 0.74 | T | C | 0.017 | 0.003 | 2.21×10^-10^ | 0.007 | 0.007 | 0.295 | 0.009 | 0.009 | 0.320 | -0.011 | 0.013 | 0.424 |
| rs3800230 | 6 | 108998128 | 0.88 | T | G | 0.023 | 0.004 | 7.57×10^-11^ | 0.004 | 0.009 | 0.657 | -0.007 | 0.012 | 0.547 | -0.003 | 0.018 | 0.849 |
| rs3806114 | 6 | 20482335 | 0.69 | A | G | -0.014 | 0.002 | 4.49×10^-09^ | -0.019 | 0.007 | 4.28×10^-03^ | -0.019 | 0.008 | 2.00×10^-02^ | -0.034 | 0.012 | 5.99×10^-03^ |
| rs3865018 | 15 | 67892766 | 0.27 | T | C | -0.021 | 0.003 | 1.13×10^-17^ | 0.002 | 0.007 | 0.766 | 0.002 | 0.008 | 0.855 | -0.004 | 0.013 | 0.751 |
| rs4076427 | 17 | 79087537 | 0.40 | C | G | -0.015 | 0.002 | 8.93×10^-10^ | -0.003 | 0.007 | 0.611 | -0.011 | 0.008 | 0.177 | 0.005 | 0.013 | 0.707 |
| rs427943 | 21 | 46570896 | 0.43 | A | C | -0.018 | 0.002 | 1.37×10^-14^ | 0.002 | 0.006 | 0.802 | 0.006 | 0.008 | 0.446 | 0.012 | 0.012 | 0.290 |
| rs4307239 | 7 | 24354300 | 0.54 | A | G | -0.014 | 0.002 | 1.31×10^-09^ | 0.012 | 0.006 | 4.63×10^-02^ | 0.013 | 0.008 | 0.093 | 0.017 | 0.012 | 0.141 |
| rs4379706 | 1 | 98322379 | 0.79 | T | C | -0.019 | 0.003 | 5.50×10^-12^ | -0.003 | 0.007 | 0.683 | -0.007 | 0.009 | 0.446 | -0.005 | 0.013 | 0.739 |
| rs4459316 | 11 | 130756176 | 0.55 | T | C | -0.015 | 0.002 | 2.33×10^-11^ | 0.002 | 0.006 | 0.720 | 0.006 | 0.008 | 0.436 | 0.014 | 0.012 | 0.248 |
| rs4474229 | 1 | 195037001 | 0.37 | A | G | -0.017 | 0.003 | 1.64×10^-10^ | 0.003 | 0.006 | 0.593 | 0.010 | 0.008 | 0.199 | -0.002 | 0.012 | 0.875 |
| rs4506565 | 10 | 114756041 | 0.70 | A | T | 0.015 | 0.002 | 5.35×10^-10^ | -0.035 | 0.007 | 1.21×10^-07^ | -0.028 | 0.008 | 4.59×10^-04^ | -0.044 | 0.012 | 4.06×10^-04^ |
| rs459552 | 5 | 112176756 | 0.78 | A | T | -0.016 | 0.003 | 4.55×10^-09^ | 0.004 | 0.007 | 0.541 | 0.013 | 0.009 | 0.150 | -0.004 | 0.013 | 0.749 |
| rs4740619 | 9 | 15634326 | 0.54 | T | C | 0.020 | 0.002 | 3.01×10^-19^ | 0.006 | 0.006 | 0.297 | 0.005 | 0.007 | 0.515 | 0.020 | 0.011 | 0.087 |
| rs4790292 | 17 | 1824305 | 0.15 | A | C | -0.026 | 0.003 | 1.65×10^-15^ | 0.013 | 0.009 | 0.153 | 0.018 | 0.011 | 0.104 | 0.021 | 0.017 | 0.201 |
| rs4834272 | 4 | 113313986 | 0.69 | T | C | -0.014 | 0.002 | 8.24×10^-10^ | -0.002 | 0.007 | 0.798 | 0.000 | 0.008 | 0.983 | -0.021 | 0.013 | 0.092 |
| rs4880341 | 10 | 133992689 | 0.57 | T | C | -0.014 | 0.002 | 4.53×10^-10^ | -0.001 | 0.006 | 0.888 | 0.000 | 0.008 | 0.971 | -0.004 | 0.011 | 0.761 |
| rs4895231 | 5 | 119388659 | 0.47 | C | G | 0.014 | 0.002 | 9.26×10^-10^ | 0.007 | 0.006 | 0.240 | 0.000 | 0.008 | 0.998 | 0.009 | 0.012 | 0.425 |
| rs4906263 | 14 | 103249127 | 0.66 | C | G | -0.017 | 0.002 | 3.05×10^-12^ | 0.021 | 0.006 | 8.78×10^-04^ | 0.025 | 0.008 | 1.41×10^-03^ | 0.008 | 0.012 | 0.499 |
| rs4929923 | 11 | 8639200 | 0.34 | T | C | -0.022 | 0.002 | 2.51×10^-22^ | -0.002 | 0.006 | 0.800 | 0.001 | 0.008 | 0.948 | -0.002 | 0.012 | 0.837 |
| rs5011579 | 16 | 69187318 | 0.28 | C | G | -0.018 | 0.003 | 1.44×10^-09^ | -0.006 | 0.007 | 0.345 | 0.005 | 0.008 | 0.556 | -0.019 | 0.013 | 0.139 |
| rs543874 | 1 | 177889480 | 0.78 | A | G | -0.058 | 0.003 | 8.70×10^-100^ | 0.034 | 0.008 | 1.00×10^-05^ | 0.025 | 0.009 | 7.28×10^-03^ | 0.014 | 0.014 | 0.327 |
| rs563296 | 10 | 99772404 | 0.56 | A | G | 0.018 | 0.002 | 8.85×10^-14^ | 0.005 | 0.006 | 0.452 | 0.000 | 0.008 | 0.981 | 0.015 | 0.012 | 0.205 |
| rs57800857 | 4 | 140863365 | 0.63 | A | C | 0.019 | 0.003 | 1.07×10^-11^ | 0.011 | 0.006 | 0.085 | 0.011 | 0.008 | 0.150 | 0.032 | 0.012 | 7.30×10^-03^ |
| rs592483 | 11 | 69445173 | 0.59 | T | C | -0.015 | 0.002 | 4.73×10^-11^ | -0.008 | 0.006 | 0.179 | -0.006 | 0.007 | 0.387 | 0.003 | 0.011 | 0.784 |
| rs613872 | 18 | 53210302 | 0.83 | T | G | 0.019 | 0.003 | 2.46×10^-10^ | 0.005 | 0.008 | 0.573 | -0.007 | 0.010 | 0.464 | 0.026 | 0.015 | 0.092 |
| rs61813324 | 1 | 156049877 | 0.13 | T | C | 0.030 | 0.004 | 3.60×10^-14^ | -0.019 | 0.010 | 0.054 | -0.027 | 0.012 | 2.66×10^-02^ | -0.003 | 0.019 | 0.862 |
| rs6265 | 11 | 27679916 | 0.18 | T | C | -0.037 | 0.003 | 7.95×10^-40^ | -0.009 | 0.008 | 0.216 | -0.001 | 0.009 | 0.955 | -0.018 | 0.014 | 0.200 |
| rs630602 | 1 | 54728864 | 0.61 | C | G | 0.015 | 0.002 | 4.55×10^-10^ | 0.002 | 0.006 | 0.753 | 0.005 | 0.008 | 0.552 | 0.000 | 0.012 | 0.970 |
| rs6436755 | 2 | 228999691 | 0.43 | A | G | 0.013 | 0.002 | 2.57×10^-09^ | -0.005 | 0.006 | 0.450 | -0.003 | 0.008 | 0.674 | 0.008 | 0.012 | 0.523 |
| rs645026 | 12 | 69757931 | 0.74 | A | G | -0.016 | 0.003 | 4.79×10^-10^ | 0.017 | 0.007 | 1.91×10^-02^ | 0.013 | 0.009 | 0.137 | 0.028 | 0.014 | 4.20×10^-02^ |
| rs6489156 | 12 | 123100397 | 0.73 | T | C | 0.022 | 0.003 | 9.97×10^-18^ | 0.013 | 0.007 | 0.056 | 0.019 | 0.008 | 2.62×10^-02^ | 0.004 | 0.013 | 0.735 |
| rs6490117 | 12 | 117599349 | 0.53 | C | G | -0.015 | 0.002 | 1.84×10^-10^ | -0.008 | 0.006 | 0.187 | -0.005 | 0.008 | 0.516 | -0.012 | 0.011 | 0.276 |
| rs6493498 | 15 | 51754451 | 0.45 | T | C | 0.016 | 0.002 | 1.53×10^-13^ | -0.010 | 0.006 | 0.098 | -0.020 | 0.008 | 7.60×10^-03^ | 0.015 | 0.012 | 0.195 |
| rs6539064 | 12 | 103706754 | 0.75 | C | G | 0.019 | 0.003 | 2.77×10^-13^ | -0.019 | 0.007 | 4.74×10^-03^ | -0.014 | 0.008 | 0.092 | -0.015 | 0.013 | 0.243 |
| rs6548237 | 2 | 621461 | 0.16 | A | C | -0.058 | 0.003 | 4.43×10^-89^ | 0.020 | 0.010 | 0.050 | 0.019 | 0.013 | 0.122 | 0.059 | 0.019 | 1.79×10^-03^ |
| rs6567160 | 18 | 57829135 | 0.75 | T | C | -0.057 | 0.003 | 8.74×10^-108^ | 0.021 | 0.007 | 3.64×10^-03^ | 0.013 | 0.009 | 0.131 | 0.025 | 0.013 | 0.061 |
| rs6606686 | 12 | 110903380 | 0.70 | C | G | -0.018 | 0.002 | 2.50×10^-14^ | -0.002 | 0.006 | 0.720 | -0.007 | 0.008 | 0.376 | 0.003 | 0.012 | 0.801 |
| rs6720868 | 2 | 230663576 | 0.31 | T | C | 0.017 | 0.002 | 4.65×10^-12^ | -0.012 | 0.006 | 0.064 | -0.009 | 0.008 | 0.232 | -0.003 | 0.012 | 0.785 |
| rs6739199 | 2 | 105452326 | 0.27 | T | C | 0.016 | 0.003 | 3.20×10^-10^ | 0.000 | 0.007 | 0.971 | 0.004 | 0.009 | 0.674 | -0.004 | 0.014 | 0.760 |
| rs6857 | 19 | 45392254 | 0.17 | T | C | -0.026 | 0.003 | 4.56×10^-17^ | -0.016 | 0.008 | 0.059 | -0.025 | 0.011 | 1.67×10^-02^ | -0.009 | 0.016 | 0.557 |
| rs6885199 | 5 | 92574633 | 0.04 | A | G | 0.039 | 0.006 | 8.52×10^-10^ | 0.011 | 0.017 | 0.530 | 0.031 | 0.021 | 0.136 | -0.017 | 0.033 | 0.619 |
| rs6890310 | 5 | 27193573 | 0.29 | A | G | -0.015 | 0.003 | 1.38×10^-09^ | 0.008 | 0.007 | 0.241 | 0.008 | 0.009 | 0.366 | -0.007 | 0.013 | 0.581 |
| rs6944634 | 7 | 75061769 | 0.80 | C | G | -0.022 | 0.003 | 7.38×10^-16^ | -0.003 | 0.008 | 0.689 | 0.014 | 0.010 | 0.149 | -0.019 | 0.015 | 0.204 |
| rs7024334 | 9 | 109072075 | 0.23 | T | G | 0.016 | 0.003 | 1.63×10^-09^ | 0.016 | 0.007 | 2.79×10^-02^ | 0.025 | 0.009 | 5.79×10^-03^ | 0.013 | 0.014 | 0.331 |
| rs704061 | 12 | 89771903 | 0.56 | T | C | -0.016 | 0.002 | 1.01×10^-11^ | 0.003 | 0.007 | 0.674 | 0.004 | 0.008 | 0.622 | -0.007 | 0.013 | 0.574 |
| rs7083450 | 10 | 103984060 | 0.83 | T | C | 0.019 | 0.003 | 9.26×10^-10^ | -0.021 | 0.008 | 9.01×10^-03^ | -0.039 | 0.010 | 8.27×10^-05^ | -0.018 | 0.015 | 0.252 |
| rs7084454 | 10 | 21821274 | 0.31 | A | G | 0.023 | 0.003 | 7.43×10^-20^ | 0.044 | 0.006 | 5.14×10^-12^ | 0.056 | 0.008 | 1.50×10^-12^ | -0.041 | 0.012 | 9.32×10^-04^ |
| rs709400 | 14 | 104149475 | 0.62 | A | G | 0.018 | 0.002 | 4.41×10^-16^ | -0.020 | 0.006 | 1.08×10^-03^ | -0.021 | 0.008 | 6.00×10^-03^ | -0.015 | 0.012 | 0.200 |
| rs7124681 | 11 | 47529947 | 0.42 | A | C | 0.025 | 0.002 | 9.27×10^-29^ | -0.005 | 0.006 | 0.409 | -0.010 | 0.008 | 0.176 | -0.010 | 0.012 | 0.383 |
| rs7133378 | 12 | 124409502 | 0.33 | A | G | 0.016 | 0.002 | 1.85×10^-11^ | 0.010 | 0.007 | 0.137 | 0.007 | 0.008 | 0.377 | -0.003 | 0.013 | 0.834 |
| rs7138803 | 12 | 50247468 | 0.39 | A | G | 0.029 | 0.002 | 9.70×10^-38^ | -0.004 | 0.006 | 0.560 | -0.013 | 0.008 | 0.085 | 0.005 | 0.012 | 0.681 |
| rs7141420 | 14 | 79899454 | 0.55 | T | C | 0.021 | 0.002 | 7.84×10^-22^ | 0.012 | 0.006 | 0.057 | 0.012 | 0.008 | 0.127 | 0.009 | 0.012 | 0.437 |
| rs7147503 | 14 | 101539384 | 0.35 | T | C | -0.014 | 0.002 | 1.91×10^-09^ | 0.008 | 0.006 | 0.193 | -0.001 | 0.008 | 0.904 | 0.018 | 0.012 | 0.129 |
| rs715 | 2 | 211543055 | 0.70 | T | C | -0.019 | 0.003 | 2.22×10^-13^ | -0.011 | 0.007 | 0.092 | -0.002 | 0.008 | 0.826 | -0.040 | 0.013 | 1.65×10^-03^ |
| rs7185966 | 16 | 4022703 | 0.24 | A | G | -0.021 | 0.003 | 1.63×10^-11^ | -0.032 | 0.007 | 1.17×10^-05^ | -0.028 | 0.009 | 2.40×10^-03^ | -0.044 | 0.014 | 1.63×10^-03^ |
| rs7186893 | 16 | 24806420 | 0.26 | T | G | -0.017 | 0.003 | 2.07×10^-10^ | -0.006 | 0.007 | 0.365 | -0.006 | 0.008 | 0.509 | -0.020 | 0.013 | 0.127 |
| rs7200589 | 16 | 349331 | 0.26 | A | G | -0.019 | 0.003 | 3.53×10^-13^ | 0.005 | 0.007 | 0.502 | -0.004 | 0.008 | 0.615 | -0.014 | 0.013 | 0.274 |
| rs7211966 | 17 | 54374611 | 0.03 | T | C | -0.047 | 0.008 | 4.89×10^-09^ | 0.001 | 0.026 | 0.978 | 0.017 | 0.033 | 0.616 | -0.010 | 0.051 | 0.852 |
| rs7238896 | 18 | 1840658 | 0.86 | A | G | -0.023 | 0.004 | 7.13×10^-10^ | 0.004 | 0.009 | 0.667 | 0.002 | 0.011 | 0.838 | -0.024 | 0.016 | 0.148 |
| rs7239114 | 18 | 45921214 | 0.54 | A | G | 0.016 | 0.002 | 3.90×10^-12^ | -0.004 | 0.006 | 0.551 | 0.008 | 0.008 | 0.303 | -0.005 | 0.012 | 0.688 |
| rs7247614 | 19 | 33953354 | 0.69 | T | G | -0.016 | 0.003 | 2.40×10^-10^ | 0.009 | 0.007 | 0.184 | 0.012 | 0.008 | 0.136 | -0.003 | 0.013 | 0.793 |
| rs7259070 | 19 | 47562509 | 0.41 | T | C | -0.019 | 0.003 | 1.06×10^-12^ | 0.021 | 0.006 | 1.07×10^-03^ | 0.021 | 0.008 | 9.16×10^-03^ | 0.033 | 0.012 | 7.70×10^-03^ |
| rs72618637 | 2 | 48953979 | 0.19 | A | T | -0.021 | 0.003 | 6.86×10^-10^ | 0.004 | 0.008 | 0.576 | -0.005 | 0.010 | 0.622 | 0.009 | 0.015 | 0.538 |
| rs7318817 | 13 | 28617708 | 0.63 | T | C | -0.014 | 0.002 | 2.15×10^-09^ | -0.009 | 0.006 | 0.132 | -0.006 | 0.008 | 0.402 | 0.003 | 0.012 | 0.798 |
| rs73213484 | 4 | 28489339 | 0.86 | A | T | 0.025 | 0.004 | 2.28×10^-11^ | 0.003 | 0.008 | 0.730 | 0.008 | 0.010 | 0.438 | -0.003 | 0.016 | 0.833 |
| rs7335249 | 13 | 109962579 | 0.56 | T | C | 0.014 | 0.002 | 4.91×10^-09^ | 0.002 | 0.006 | 0.770 | -0.007 | 0.008 | 0.403 | 0.009 | 0.012 | 0.457 |
| rs73646205 | 9 | 16724055 | 0.17 | A | T | 0.024 | 0.004 | 1.93×10^-11^ | 0.014 | 0.009 | 0.098 | 0.017 | 0.011 | 0.113 | 0.000 | 0.016 | 0.982 |
| rs7428670 | 3 | 131579810 | 0.27 | C | G | 0.020 | 0.003 | 3.09×10^-15^ | -0.004 | 0.007 | 0.543 | -0.009 | 0.009 | 0.295 | -0.001 | 0.013 | 0.922 |
| rs7498665 | 16 | 28883241 | 0.62 | A | G | -0.026 | 0.002 | 1.94×10^-32^ | -0.005 | 0.006 | 0.428 | -0.009 | 0.008 | 0.216 | 0.006 | 0.012 | 0.613 |
| rs7550711 | 1 | 110082886 | 0.03 | T | C | 0.064 | 0.007 | 3.37×10^-21^ | -0.067 | 0.017 | 1.07×10^-04^ | -0.041 | 0.021 | 0.057 | -0.102 | 0.034 | 2.30×10^-03^ |
| rs7579277 | 2 | 28996295 | 0.67 | A | G | -0.016 | 0.002 | 5.84×10^-11^ | -0.010 | 0.006 | 0.120 | -0.022 | 0.008 | 5.27×10^-03^ | 0.028 | 0.012 | 2.36×10^-02^ |
| rs7582359 | 2 | 55286681 | 0.33 | A | G | -0.017 | 0.003 | 9.96×10^-12^ | 0.005 | 0.007 | 0.485 | 0.010 | 0.008 | 0.244 | 0.005 | 0.013 | 0.687 |
| rs7588437 | 2 | 181575281 | 0.37 | A | G | -0.018 | 0.002 | 8.38×10^-15^ | 0.015 | 0.006 | 1.53×10^-02^ | 0.008 | 0.008 | 0.313 | 0.036 | 0.012 | 2.47×10^-03^ |
| rs7599312 | 2 | 213413231 | 0.27 | A | G | -0.016 | 0.003 | 1.07×10^-10^ | 0.020 | 0.007 | 4.15×10^-03^ | 0.027 | 0.008 | 1.36×10^-03^ | 0.024 | 0.013 | 0.062 |
| rs7621025 | 3 | 136272246 | 0.26 | T | C | -0.020 | 0.003 | 1.62×10^-15^ | 0.013 | 0.007 | 0.053 | 0.008 | 0.009 | 0.361 | 0.035 | 0.013 | 7.88×10^-03^ |
| rs7701842 | 5 | 64074730 | 0.49 | A | T | 0.014 | 0.002 | 5.95×10^-10^ | -0.010 | 0.006 | 0.109 | -0.011 | 0.008 | 0.144 | -0.013 | 0.012 | 0.252 |
| rs7701886 | 5 | 153547409 | 0.54 | A | G | -0.016 | 0.002 | 2.38×10^-12^ | 0.011 | 0.006 | 0.078 | 0.004 | 0.008 | 0.575 | 0.019 | 0.012 | 0.096 |
| rs7713317 | 5 | 95716722 | 0.72 | A | G | -0.015 | 0.002 | 2.70×10^-10^ | -0.009 | 0.007 | 0.193 | -0.007 | 0.009 | 0.406 | -0.005 | 0.013 | 0.698 |
| rs77432547 | 13 | 86494817 | 0.72 | A | G | -0.018 | 0.003 | 2.28×10^-09^ | 0.013 | 0.007 | 0.053 | 0.011 | 0.008 | 0.209 | 0.003 | 0.013 | 0.801 |
| rs782971 | 5 | 43124688 | 0.75 | A | G | -0.017 | 0.003 | 4.89×10^-12^ | -0.014 | 0.007 | 0.055 | -0.018 | 0.009 | 4.57×10^-02^ | -0.006 | 0.014 | 0.668 |
| rs7845090 | 8 | 73449940 | 0.70 | A | G | -0.023 | 0.003 | 1.50×10^-15^ | -0.008 | 0.007 | 0.231 | -0.015 | 0.009 | 0.075 | -0.012 | 0.013 | 0.337 |
| rs7899106 | 10 | 87410904 | 0.95 | A | G | -0.038 | 0.005 | 7.65×10^-14^ | 0.023 | 0.014 | 0.110 | 0.029 | 0.018 | 0.104 | -0.025 | 0.027 | 0.350 |
| rs79113395 | 1 | 1590521 | 0.27 | A | G | -0.021 | 0.003 | 1.45×10^-12^ | -0.009 | 0.010 | 0.358 | -0.012 | 0.012 | 0.327 | -0.009 | 0.019 | 0.648 |
| rs7961979 | 12 | 121671261 | 0.13 | A | C | 0.021 | 0.004 | 2.40×10^-09^ | 0.008 | 0.010 | 0.401 | 0.010 | 0.012 | 0.388 | 0.003 | 0.018 | 0.874 |
| rs7975187 | 12 | 60964108 | 0.77 | A | G | -0.017 | 0.003 | 1.22×10^-09^ | 0.007 | 0.007 | 0.344 | 0.013 | 0.009 | 0.169 | -0.002 | 0.014 | 0.862 |
| rs7976757 | 12 | 19207948 | 0.18 | T | C | -0.019 | 0.003 | 2.94×10^-10^ | -0.006 | 0.008 | 0.489 | 0.005 | 0.010 | 0.628 | -0.010 | 0.015 | 0.501 |
| rs8016859 | 14 | 30484722 | 0.04 | C | G | 0.036 | 0.006 | 3.83×10^-10^ | -0.016 | 0.016 | 0.328 | -0.017 | 0.020 | 0.410 | -0.020 | 0.031 | 0.516 |
| rs8038522 | 15 | 46585105 | 0.41 | T | G | -0.014 | 0.002 | 1.27×10^-09^ | -0.009 | 0.006 | 0.148 | -0.002 | 0.008 | 0.768 | 0.003 | 0.012 | 0.794 |
| rs8075273 | 17 | 61728881 | 0.29 | A | C | -0.016 | 0.002 | 5.56×10^-11^ | 0.011 | 0.007 | 0.106 | 0.010 | 0.008 | 0.255 | 0.010 | 0.013 | 0.429 |
| rs811054 | 16 | 72251132 | 0.53 | T | C | 0.016 | 0.002 | 3.18×10^-13^ | 0.019 | 0.006 | 1.78×10^-03^ | 0.022 | 0.008 | 4.00×10^-03^ | 0.006 | 0.012 | 0.593 |
| rs867560 | 9 | 129465233 | 0.57 | C | G | -0.014 | 0.002 | 5.56×10^-10^ | 0.016 | 0.006 | 1.01×10^-02^ | 0.014 | 0.008 | 0.072 | 0.013 | 0.012 | 0.257 |
| rs890235 | 8 | 64716469 | 0.55 | A | G | 0.014 | 0.002 | 4.08×10^-10^ | -0.001 | 0.006 | 0.892 | 0.002 | 0.008 | 0.828 | -0.001 | 0.012 | 0.970 |
| rs901630 | 6 | 98539519 | 0.42 | T | C | -0.015 | 0.002 | 4.56×10^-11^ | -0.011 | 0.006 | 0.063 | -0.013 | 0.008 | 0.088 | -0.011 | 0.012 | 0.354 |
| rs923724 | 12 | 99639930 | 0.62 | C | G | -0.016 | 0.002 | 5.74×10^-11^ | 0.007 | 0.006 | 0.240 | 0.015 | 0.008 | 0.061 | -0.006 | 0.012 | 0.631 |
| rs9302652 | 16 | 53865975 | 0.74 | T | C | -0.021 | 0.003 | 8.92×10^-18^ | -0.027 | 0.007 | 5.78×10^-05^ | -0.013 | 0.008 | 0.106 | -0.062 | 0.013 | 6.66×10^-07^ |
| rs9367369 | 6 | 13189941 | 0.71 | A | G | 0.017 | 0.003 | 2.32×10^-09^ | 0.001 | 0.007 | 0.898 | -0.001 | 0.008 | 0.929 | 0.020 | 0.012 | 0.112 |
| rs942066 | 14 | 94031914 | 0.37 | A | G | -0.020 | 0.003 | 1.04×10^-13^ | 0.002 | 0.006 | 0.729 | 0.006 | 0.008 | 0.462 | -0.005 | 0.012 | 0.700 |
| rs943005 | 6 | 50865820 | 0.15 | T | C | 0.040 | 0.003 | 1.99×10^-43^ | 0.011 | 0.008 | 0.179 | 0.022 | 0.010 | 2.19×10^-02^ | -0.032 | 0.015 | 3.36×10^-02^ |
| rs947088 | 20 | 17171373 | 0.74 | T | G | 0.016 | 0.003 | 1.91×10^-10^ | -0.009 | 0.007 | 0.184 | -0.012 | 0.008 | 0.147 | -0.007 | 0.013 | 0.553 |
| rs947791 | 11 | 65302893 | 0.21 | A | G | 0.020 | 0.003 | 5.88×10^-13^ | 0.022 | 0.008 | 5.01×10^-03^ | 0.015 | 0.010 | 0.124 | 0.017 | 0.015 | 0.249 |
| rs953567 | 1 | 74977425 | 0.59 | A | G | -0.021 | 0.002 | 1.17×10^-20^ | 0.003 | 0.006 | 0.643 | 0.009 | 0.008 | 0.240 | 0.014 | 0.012 | 0.247 |
| rs9540493 | 13 | 66205704 | 0.46 | A | G | 0.014 | 0.002 | 1.72×10^-10^ | 0.005 | 0.006 | 0.449 | 0.011 | 0.008 | 0.186 | 0.000 | 0.012 | 0.980 |
| rs9571687 | 13 | 67472713 | 0.34 | A | C | -0.015 | 0.002 | 3.24×10^-09^ | -0.006 | 0.007 | 0.338 | -0.006 | 0.008 | 0.479 | -0.004 | 0.012 | 0.773 |
| rs9595908 | 13 | 33184288 | 0.64 | T | C | 0.014 | 0.002 | 2.34×10^-10^ | -0.007 | 0.006 | 0.228 | -0.018 | 0.008 | 1.86×10^-02^ | 0.010 | 0.012 | 0.375 |
| rs9636391 | 2 | 50201110 | 0.16 | A | G | 0.021 | 0.003 | 7.08×10^-12^ | 0.013 | 0.008 | 0.120 | 0.027 | 0.010 | 7.55×10^-03^ | 0.007 | 0.015 | 0.666 |
| rs9839267 | 3 | 42332624 | 0.89 | T | G | 0.024 | 0.004 | 1.09×10^-11^ | -0.016 | 0.010 | 0.123 | -0.025 | 0.013 | 0.052 | -0.025 | 0.020 | 0.206 |
| rs9862795 | 3 | 49915506 | 0.48 | A | T | -0.027 | 0.003 | 5.30×10^-25^ | -0.016 | 0.006 | 9.15×10^-03^ | -0.018 | 0.007 | 1.27×10^-02^ | -0.033 | 0.011 | 3.90×10^-03^ |
| rs9906944 | 17 | 47091420 | 0.36 | T | C | -0.019 | 0.002 | 7.25×10^-15^ | -0.004 | 0.007 | 0.571 | -0.006 | 0.008 | 0.457 | 0.001 | 0.013 | 0.938 |
| rs9910745 | 17 | 78610543 | 0.45 | T | C | -0.019 | 0.002 | 7.44×10^-16^ | 0.007 | 0.006 | 0.279 | 0.007 | 0.008 | 0.373 | 0.003 | 0.012 | 0.835 |
| rs9929792 | 16 | 20371075 | 0.50 | A | T | 0.019 | 0.002 | 3.80×10^-16^ | -0.009 | 0.006 | 0.120 | -0.005 | 0.007 | 0.464 | -0.008 | 0.011 | 0.491 |
| **WHR (female)** |  |  |  |  |  |  |  |  |  |  |  |  |  |  |  |  |  |
| rs10019888 | 4 | 26062990 | 0.81 | A | G | -0.032 | 0.003 | 1.10×10^-24^ | 0.000 | 0.008 | 0.962 | -0.016 | 0.010 | 0.121 | 0.008 | 0.016 | 0.610 |
| rs10049088 | 3 | 156797648 | 0.38 | T | C | -0.028 | 0.002 | 5.04×10^-33^ | -0.005 | 0.006 | 0.444 | -0.003 | 0.008 | 0.685 | -0.012 | 0.012 | 0.289 |
| rs1006195 | 11 | 118958869 | 0.39 | T | G | 0.015 | 0.002 | 4.55×10^-10^ | -0.014 | 0.006 | 2.91×10^-02^ | -0.004 | 0.008 | 0.609 | -0.011 | 0.012 | 0.369 |
| rs10101067 | 8 | 72407374 | 0.07 | C | G | 0.047 | 0.005 | 2.28×10^-25^ | -0.019 | 0.012 | 0.128 | -0.030 | 0.015 | 4.98×10^-02^ | -0.024 | 0.023 | 0.313 |
| rs10116353 | 9 | 111950088 | 0.30 | T | G | -0.016 | 0.003 | 2.21×10^-10^ | 0.001 | 0.007 | 0.903 | 0.003 | 0.008 | 0.745 | -0.013 | 0.012 | 0.277 |
| rs1035940 | 19 | 7199978 | 0.28 | C | G | 0.019 | 0.003 | 7.30×10^-13^ | -0.003 | 0.007 | 0.603 | 0.004 | 0.008 | 0.660 | -0.002 | 0.013 | 0.856 |
| rs1045241 | 5 | 118729286 | 0.28 | T | C | -0.026 | 0.003 | 1.93×10^-23^ | 0.011 | 0.007 | 0.114 | 0.012 | 0.008 | 0.155 | -0.027 | 0.013 | 3.44×10^-02^ |
| rs1045411 | 13 | 31033232 | 0.28 | T | C | -0.016 | 0.003 | 2.91×10^-09^ | -0.014 | 0.008 | 0.066 | -0.009 | 0.010 | 0.358 | -0.019 | 0.015 | 0.186 |
| rs10460716 | 21 | 39456226 | 0.46 | A | T | -0.017 | 0.002 | 4.29×10^-12^ | 0.002 | 0.006 | 0.798 | -0.003 | 0.008 | 0.648 | 0.022 | 0.011 | 0.058 |
| rs10462028 | 4 | 56298300 | 0.32 | A | G | 0.019 | 0.003 | 1.19×10^-14^ | -0.010 | 0.006 | 0.108 | -0.011 | 0.008 | 0.173 | -0.021 | 0.012 | 0.082 |
| rs10761785 | 10 | 65318766 | 0.51 | T | G | -0.017 | 0.002 | 2.00×10^-14^ | -0.025 | 0.006 | 2.93×10^-05^ | -0.030 | 0.007 | 6.08×10^-05^ | -0.005 | 0.011 | 0.670 |
| rs10788569 | 10 | 89604732 | 0.72 | T | C | -0.018 | 0.003 | 2.50×10^-12^ | -0.003 | 0.007 | 0.683 | -0.021 | 0.009 | 1.53×10^-02^ | 0.008 | 0.013 | 0.558 |
| rs10795055 | 10 | 3581221 | 0.39 | A | G | 0.016 | 0.002 | 3.94×10^-11^ | -0.008 | 0.006 | 0.175 | -0.008 | 0.008 | 0.321 | -0.013 | 0.012 | 0.282 |
| rs10808546 | 8 | 126495818 | 0.42 | T | C | -0.018 | 0.002 | 9.46×10^-16^ | 0.013 | 0.006 | 3.47×10^-02^ | 0.014 | 0.008 | 0.067 | 0.015 | 0.012 | 0.201 |
| rs10842707 | 12 | 26471364 | 0.20 | T | C | 0.039 | 0.003 | 1.61×10^-46^ | 0.005 | 0.007 | 0.506 | 0.010 | 0.009 | 0.274 | 0.019 | 0.014 | 0.156 |
| rs10875120 | 1 | 98399903 | 0.78 | T | G | -0.020 | 0.003 | 1.59×10^-12^ | 0.000 | 0.007 | 0.949 | 0.000 | 0.009 | 0.982 | 0.001 | 0.014 | 0.953 |
| rs10891290 | 11 | 111605215 | 0.65 | T | C | 0.024 | 0.002 | 3.01×10^-23^ | 0.010 | 0.006 | 0.098 | 0.012 | 0.008 | 0.108 | -0.005 | 0.012 | 0.685 |
| rs10919388 | 1 | 170372503 | 0.27 | A | C | -0.037 | 0.003 | 9.05×10^-47^ | -0.008 | 0.007 | 0.248 | -0.009 | 0.009 | 0.315 | -0.031 | 0.013 | 1.85×10^-02^ |
| rs10980797 | 9 | 113912553 | 0.52 | A | G | -0.022 | 0.003 | 6.95×10^-16^ | 0.009 | 0.006 | 0.126 | 0.012 | 0.008 | 0.119 | 0.015 | 0.011 | 0.189 |
| rs11075985 | 16 | 53805207 | 0.44 | A | C | 0.033 | 0.002 | 4.15×10^-46^ | -0.055 | 0.006 | 1.37×10^-19^ | -0.054 | 0.007 | 4.67×10^-13^ | -0.074 | 0.011 | 9.01×10^-11^ |
| rs11187537 | 10 | 95346805 | 0.26 | C | G | 0.021 | 0.003 | 3.52×10^-15^ | 0.008 | 0.007 | 0.255 | 0.008 | 0.009 | 0.392 | 0.008 | 0.014 | 0.545 |
| rs1122157 | 10 | 63861600 | 0.31 | T | C | -0.020 | 0.003 | 4.85×10^-14^ | -0.014 | 0.007 | 3.66×10^-02^ | -0.012 | 0.008 | 0.139 | -0.011 | 0.013 | 0.374 |
| rs1139653 | 16 | 4484396 | 0.29 | A | T | 0.017 | 0.003 | 1.70×10^-10^ | -0.002 | 0.007 | 0.836 | -0.008 | 0.009 | 0.425 | -0.007 | 0.014 | 0.639 |
| rs114760566 | 6 | 34192036 | 0.04 | A | C | 0.097 | 0.007 | 8.92×10^-50^ | -0.018 | 0.016 | 0.247 | -0.024 | 0.019 | 0.213 | -0.021 | 0.030 | 0.492 |
| rs1152008 | 3 | 12487612 | 0.63 | T | G | 0.025 | 0.002 | 3.45×10^-27^ | 0.009 | 0.006 | 0.131 | 0.009 | 0.008 | 0.267 | 0.004 | 0.012 | 0.739 |
| rs11603150 | 11 | 36373869 | 0.69 | T | C | 0.020 | 0.003 | 2.77×10^-12^ | 0.001 | 0.006 | 0.936 | 0.010 | 0.008 | 0.203 | -0.015 | 0.012 | 0.216 |
| rs11666808 | 19 | 18383506 | 0.37 | T | C | 0.030 | 0.003 | 1.44×10^-27^ | 0.009 | 0.006 | 0.179 | 0.003 | 0.008 | 0.687 | 0.003 | 0.012 | 0.817 |
| rs11694173 | 2 | 43590899 | 0.18 | A | G | 0.020 | 0.003 | 2.98×10^-12^ | 0.011 | 0.008 | 0.163 | 0.018 | 0.009 | 4.99×10^-02^ | 0.013 | 0.014 | 0.352 |
| rs1169809 | 12 | 121688026 | 0.31 | A | G | 0.016 | 0.003 | 2.05×10^-09^ | 0.008 | 0.007 | 0.234 | 0.011 | 0.009 | 0.215 | -0.002 | 0.013 | 0.873 |
| rs11714441 | 3 | 123158946 | 0.40 | T | C | 0.017 | 0.003 | 1.22×10^-09^ | 0.004 | 0.007 | 0.517 | 0.016 | 0.008 | 0.057 | 0.008 | 0.013 | 0.522 |
| rs11727676 | 4 | 145659064 | 0.91 | T | C | -0.024 | 0.004 | 4.94×10^-09^ | 0.012 | 0.011 | 0.297 | 0.013 | 0.014 | 0.328 | -0.017 | 0.021 | 0.410 |
| rs11756568 | 6 | 152042413 | 0.74 | A | T | -0.016 | 0.003 | 1.91×10^-09^ | -0.048 | 0.007 | 3.45×10^-13^ | -0.035 | 0.008 | 2.00×10^-05^ | -0.072 | 0.013 | 7.24×10^-09^ |
| rs11760290 | 7 | 107616068 | 0.90 | T | C | 0.029 | 0.004 | 9.78×10^-13^ | 0.008 | 0.011 | 0.485 | 0.004 | 0.014 | 0.766 | 0.015 | 0.022 | 0.475 |
| rs11764879 | 7 | 77333267 | 0.29 | A | G | -0.017 | 0.003 | 1.17×10^-11^ | -0.001 | 0.007 | 0.912 | -0.003 | 0.009 | 0.746 | 0.013 | 0.014 | 0.339 |
| rs11897119 | 2 | 66772000 | 0.59 | T | C | -0.019 | 0.002 | 1.41×10^-15^ | -0.010 | 0.006 | 0.109 | -0.011 | 0.008 | 0.150 | -0.004 | 0.012 | 0.760 |
| rs1190982 | 14 | 58815839 | 0.30 | T | C | 0.019 | 0.003 | 7.02×10^-14^ | 0.009 | 0.007 | 0.171 | 0.003 | 0.008 | 0.733 | 0.014 | 0.012 | 0.249 |
| rs11992444 | 8 | 25464690 | 0.51 | T | G | 0.019 | 0.003 | 1.29×10^-12^ | -0.003 | 0.008 | 0.658 | -0.003 | 0.010 | 0.781 | 0.026 | 0.015 | 0.081 |
| rs12061508 | 1 | 196584468 | 0.21 | A | G | 0.018 | 0.003 | 2.97×10^-10^ | 0.002 | 0.007 | 0.823 | 0.003 | 0.009 | 0.739 | -0.004 | 0.014 | 0.759 |
| rs12138803 | 1 | 172348823 | 0.20 | T | C | 0.029 | 0.003 | 1.03×10^-29^ | -0.007 | 0.007 | 0.327 | 0.000 | 0.009 | 0.982 | -0.021 | 0.013 | 0.109 |
| rs12325866 | 17 | 61755974 | 0.29 | A | G | -0.017 | 0.003 | 9.12×10^-11^ | 0.011 | 0.007 | 0.107 | 0.009 | 0.008 | 0.268 | 0.010 | 0.013 | 0.428 |
| rs12437696 | 15 | 40878474 | 0.51 | A | G | 0.016 | 0.003 | 1.69×10^-09^ | 0.015 | 0.006 | 1.19×10^-02^ | 0.010 | 0.007 | 0.192 | 0.027 | 0.011 | 1.87×10^-02^ |
| rs12449442 | 17 | 65947640 | 0.22 | A | G | 0.019 | 0.003 | 1.09×10^-11^ | 0.001 | 0.007 | 0.843 | -0.004 | 0.009 | 0.702 | 0.023 | 0.014 | 0.097 |
| rs12454712 | 18 | 60845884 | 0.62 | T | C | 0.022 | 0.002 | 7.15×10^-20^ | 0.007 | 0.006 | 0.233 | 0.010 | 0.008 | 0.213 | -0.016 | 0.012 | 0.191 |
| rs1250259 | 2 | 216300482 | 0.74 | A | T | -0.018 | 0.003 | 1.74×10^-11^ | 0.003 | 0.007 | 0.685 | 0.007 | 0.009 | 0.424 | -0.002 | 0.013 | 0.877 |
| rs12686771 | 9 | 95472584 | 0.16 | T | C | 0.019 | 0.003 | 2.26×10^-09^ | 0.006 | 0.009 | 0.489 | 0.004 | 0.011 | 0.720 | 0.006 | 0.017 | 0.705 |
| rs12817549 | 12 | 94121314 | 0.55 | T | C | -0.016 | 0.002 | 1.41×10^-12^ | -0.012 | 0.006 | 0.056 | -0.009 | 0.008 | 0.247 | -0.009 | 0.011 | 0.410 |
| rs1289011 | 1 | 163626413 | 0.51 | C | G | -0.014 | 0.002 | 1.13×10^-09^ | -0.010 | 0.006 | 0.122 | -0.014 | 0.008 | 0.090 | -0.011 | 0.012 | 0.368 |
| rs12943131 | 17 | 27863706 | 0.53 | A | G | -0.014 | 0.002 | 1.84×10^-09^ | 0.014 | 0.006 | 2.41×10^-02^ | 0.015 | 0.007 | 4.60×10^-02^ | 0.014 | 0.011 | 0.219 |
| rs1294432 | 6 | 6745027 | 0.54 | T | C | 0.025 | 0.002 | 3.97×10^-26^ | 0.016 | 0.006 | 7.37×10^-03^ | 0.009 | 0.007 | 0.238 | 0.011 | 0.011 | 0.320 |
| rs12982665 | 19 | 17158027 | 0.19 | T | C | -0.021 | 0.003 | 6.29×10^-10^ | 0.027 | 0.008 | 4.22×10^-04^ | 0.029 | 0.009 | 1.64×10^-03^ | 0.010 | 0.014 | 0.502 |
| rs13092573 | 3 | 46988561 | 0.33 | T | C | 0.020 | 0.003 | 6.96×10^-15^ | 0.006 | 0.006 | 0.311 | 0.020 | 0.008 | 1.28×10^-02^ | -0.009 | 0.012 | 0.460 |
| rs13101828 | 4 | 965720 | 0.55 | A | G | 0.022 | 0.003 | 1.18×10^-16^ | -0.018 | 0.006 | 3.76×10^-03^ | -0.025 | 0.008 | 1.35×10^-03^ | -0.003 | 0.012 | 0.792 |
| rs13124532 | 4 | 120416445 | 0.15 | T | C | 0.023 | 0.003 | 5.54×10^-11^ | 0.003 | 0.009 | 0.746 | -0.005 | 0.011 | 0.644 | 0.003 | 0.017 | 0.839 |
| rs13223303 | 7 | 42692711 | 0.71 | T | C | 0.017 | 0.003 | 4.25×10^-09^ | 0.010 | 0.007 | 0.157 | 0.019 | 0.009 | 2.92×10^-02^ | 0.009 | 0.013 | 0.517 |
| rs13256367 | 8 | 128334900 | 0.65 | A | C | 0.022 | 0.003 | 3.42×10^-18^ | -0.070 | 0.006 | 2.41×10^-29^ | -0.076 | 0.008 | 3.30×10^-23^ | -0.030 | 0.012 | 1.15×10^-02^ |
| rs1334576 | 6 | 7211818 | 0.43 | A | G | -0.015 | 0.002 | 1.00×10^-10^ | -0.017 | 0.006 | 4.40×10^-03^ | -0.017 | 0.008 | 2.40×10^-02^ | -0.041 | 0.012 | 3.49×10^-04^ |
| rs13432211 | 2 | 226955609 | 0.78 | A | G | 0.017 | 0.003 | 1.39×10^-09^ | -0.013 | 0.007 | 0.078 | -0.023 | 0.009 | 1.07×10^-02^ | -0.008 | 0.014 | 0.545 |
| rs1396514 | 17 | 68431026 | 0.51 | T | C | -0.028 | 0.002 | 1.95×10^-35^ | 0.003 | 0.006 | 0.601 | 0.002 | 0.007 | 0.794 | -0.002 | 0.011 | 0.887 |
| rs140201358 | 11 | 823586 | 0.99 | C | G | -0.071 | 0.012 | 1.01×10^-09^ | -0.001 | 0.032 | 0.987 | -0.054 | 0.039 | 0.168 | -0.004 | 0.063 | 0.946 |
| rs1457489 | 18 | 57861961 | 0.28 | A | G | 0.029 | 0.003 | 2.44×10^-29^ | -0.028 | 0.007 | 3.45×10^-05^ | -0.026 | 0.008 | 1.90×10^-03^ | -0.029 | 0.013 | 2.28×10^-02^ |
| rs151235402 | 20 | 569164 | 0.02 | T | C | 0.074 | 0.011 | 3.02×10^-11^ | -0.042 | 0.027 | 0.110 | -0.037 | 0.033 | 0.267 | -0.068 | 0.054 | 0.210 |
| rs1534696 | 7 | 26397239 | 0.57 | A | C | -0.034 | 0.002 | 4.00×10^-49^ | 0.008 | 0.006 | 0.174 | 0.003 | 0.008 | 0.682 | 0.014 | 0.012 | 0.231 |
| rs1535179 | 1 | 23639009 | 0.93 | A | G | -0.030 | 0.005 | 1.84×10^-10^ | -0.002 | 0.013 | 0.899 | -0.021 | 0.016 | 0.192 | 0.013 | 0.025 | 0.605 |
| rs1569135 | 2 | 188115398 | 0.54 | A | G | 0.021 | 0.002 | 5.60×10^-21^ | -0.007 | 0.006 | 0.277 | -0.013 | 0.007 | 0.077 | 0.021 | 0.011 | 0.060 |
| rs16975388 | 16 | 85261963 | 0.78 | C | G | 0.019 | 0.003 | 1.25×10^-10^ | 0.003 | 0.007 | 0.690 | -0.006 | 0.009 | 0.542 | -0.009 | 0.014 | 0.505 |
| rs17041868 | 2 | 111894720 | 0.92 | T | C | -0.031 | 0.005 | 2.84×10^-11^ | -0.059 | 0.012 | 2.15×10^-06^ | -0.075 | 0.015 | 6.52×10^-07^ | -0.045 | 0.023 | 0.053 |
| rs17101456 | 10 | 122875040 | 0.88 | A | G | -0.032 | 0.004 | 1.43×10^-17^ | 0.012 | 0.009 | 0.191 | 0.011 | 0.011 | 0.325 | -0.003 | 0.017 | 0.869 |
| rs17238775 | 15 | 56779185 | 0.88 | T | G | -0.027 | 0.004 | 1.50×10^-12^ | -0.004 | 0.010 | 0.676 | -0.001 | 0.013 | 0.945 | 0.000 | 0.020 | 0.990 |
| rs17264866 | 1 | 40022777 | 0.78 | C | G | -0.018 | 0.003 | 1.16×10^-10^ | -0.014 | 0.007 | 0.052 | -0.013 | 0.009 | 0.178 | -0.024 | 0.014 | 0.086 |
| rs17437657 | 7 | 27249617 | 0.09 | A | G | -0.028 | 0.004 | 6.62×10^-11^ | 0.000 | 0.011 | 1.000 | 0.007 | 0.014 | 0.617 | -0.001 | 0.022 | 0.978 |
| rs17457629 | 5 | 38795199 | 0.72 | A | T | 0.016 | 0.003 | 2.91×10^-09^ | -0.002 | 0.007 | 0.833 | -0.002 | 0.009 | 0.855 | 0.002 | 0.014 | 0.863 |
| rs1757471 | 10 | 34168090 | 0.51 | T | C | 0.015 | 0.002 | 3.23×10^-10^ | -0.014 | 0.006 | 2.47×10^-02^ | -0.005 | 0.008 | 0.495 | -0.022 | 0.012 | 0.056 |
| rs17584626 | 6 | 20560435 | 0.69 | A | T | 0.016 | 0.003 | 1.70×10^-09^ | 0.034 | 0.007 | 2.73×10^-07^ | 0.040 | 0.008 | 6.85×10^-07^ | 0.027 | 0.012 | 2.94×10^-02^ |
| rs17644283 | 4 | 26308792 | 0.39 | A | G | 0.015 | 0.002 | 5.46×10^-10^ | -0.006 | 0.006 | 0.337 | -0.002 | 0.008 | 0.831 | 0.005 | 0.012 | 0.660 |
| rs1799815 | 19 | 7125519 | 0.07 | A | G | -0.034 | 0.005 | 3.58×10^-10^ | 0.009 | 0.015 | 0.552 | 0.019 | 0.018 | 0.312 | -0.010 | 0.029 | 0.731 |
| rs1800437 | 19 | 46181392 | 0.19 | C | G | -0.018 | 0.003 | 5.09×10^-10^ | 0.045 | 0.008 | 6.26×10^-09^ | 0.056 | 0.010 | 5.29×10^-09^ | 0.027 | 0.015 | 0.060 |
| rs1800978 | 9 | 107665978 | 0.88 | C | G | 0.025 | 0.004 | 1.92×10^-12^ | 0.017 | 0.009 | 0.059 | 0.008 | 0.011 | 0.504 | 0.029 | 0.017 | 0.091 |
| rs1829276 | 5 | 4021401 | 0.46 | A | G | -0.018 | 0.003 | 1.76×10^-11^ | 0.005 | 0.006 | 0.365 | 0.006 | 0.008 | 0.434 | 0.001 | 0.011 | 0.905 |
| rs2033529 | 6 | 40348653 | 0.72 | A | G | -0.015 | 0.003 | 9.93×10^-10^ | 0.003 | 0.007 | 0.645 | 0.001 | 0.009 | 0.893 | -0.004 | 0.014 | 0.746 |
| rs20478 | 13 | 44686064 | 0.10 | A | G | 0.028 | 0.004 | 6.63×10^-12^ | -0.007 | 0.010 | 0.498 | -0.011 | 0.013 | 0.387 | -0.051 | 0.020 | 9.26×10^-03^ |
| rs2076586 | 1 | 112273026 | 0.23 | T | C | -0.017 | 0.003 | 3.86×10^-09^ | 0.007 | 0.007 | 0.342 | 0.004 | 0.009 | 0.640 | -0.002 | 0.014 | 0.864 |
| rs2112347 | 5 | 75015242 | 0.63 | T | G | 0.019 | 0.002 | 8.51×10^-16^ | -0.020 | 0.006 | 1.36×10^-03^ | -0.015 | 0.008 | 4.40×10^-02^ | -0.034 | 0.012 | 3.28×10^-03^ |
| rs2124307 | 14 | 98344662 | 0.37 | T | C | -0.015 | 0.002 | 2.51×10^-10^ | 0.018 | 0.006 | 4.77×10^-03^ | 0.025 | 0.008 | 1.83×10^-03^ | -0.002 | 0.012 | 0.862 |
| rs2159607 | 3 | 52501451 | 0.82 | T | G | 0.024 | 0.003 | 1.32×10^-15^ | -0.005 | 0.008 | 0.512 | -0.007 | 0.010 | 0.503 | 0.001 | 0.016 | 0.937 |
| rs2193748 | 12 | 45986471 | 0.23 | C | G | 0.020 | 0.003 | 1.93×10^-10^ | 0.009 | 0.008 | 0.253 | 0.010 | 0.010 | 0.287 | 0.000 | 0.015 | 0.997 |
| rs2207132 | 20 | 39142516 | 0.03 | A | G | 0.051 | 0.008 | 1.51×10^-11^ | 0.001 | 0.020 | 0.949 | -0.014 | 0.024 | 0.562 | 0.033 | 0.038 | 0.383 |
| rs2240702 | 19 | 4159873 | 0.89 | A | G | -0.023 | 0.004 | 9.63×10^-10^ | -0.019 | 0.011 | 0.096 | -0.021 | 0.014 | 0.135 | -0.020 | 0.021 | 0.339 |
| rs2294239 | 22 | 29449477 | 0.57 | A | G | 0.023 | 0.002 | 2.58×10^-22^ | 0.007 | 0.006 | 0.248 | 0.010 | 0.008 | 0.186 | 0.001 | 0.011 | 0.947 |
| rs2303975 | 11 | 14276999 | 0.12 | A | G | -0.021 | 0.004 | 3.07×10^-09^ | 0.022 | 0.009 | 1.94×10^-02^ | 0.029 | 0.011 | 1.07×10^-02^ | 0.008 | 0.018 | 0.640 |
| rs2306374 | 3 | 138119952 | 0.84 | T | C | -0.021 | 0.003 | 7.37×10^-12^ | -0.001 | 0.008 | 0.912 | 0.000 | 0.010 | 0.983 | 0.007 | 0.016 | 0.649 |
| rs2306589 | 17 | 34848874 | 0.47 | T | C | 0.018 | 0.002 | 2.19×10^-15^ | 0.005 | 0.007 | 0.470 | 0.008 | 0.008 | 0.313 | -0.010 | 0.012 | 0.420 |
| rs2335077 | 1 | 107573565 | 0.65 | A | G | -0.016 | 0.003 | 1.65×10^-10^ | -0.002 | 0.006 | 0.697 | -0.003 | 0.008 | 0.739 | -0.015 | 0.012 | 0.212 |
| rs2373078 | 2 | 218392555 | 0.10 | T | C | 0.028 | 0.004 | 2.38×10^-13^ | -0.019 | 0.010 | 4.92×10^-02^ | -0.015 | 0.012 | 0.217 | -0.046 | 0.019 | 1.27×10^-02^ |
| rs2448 | 5 | 53302354 | 0.74 | T | C | 0.016 | 0.003 | 4.69×10^-09^ | -0.010 | 0.007 | 0.124 | -0.017 | 0.008 | 4.91×10^-02^ | 0.001 | 0.013 | 0.927 |
| rs2455799 | 3 | 15701184 | 0.67 | T | G | -0.018 | 0.002 | 1.97×10^-13^ | -0.008 | 0.006 | 0.208 | -0.017 | 0.008 | 3.55×10^-02^ | -0.008 | 0.012 | 0.510 |
| rs2474714 | 10 | 33495983 | 0.57 | A | G | 0.015 | 0.002 | 9.38×10^-10^ | -0.011 | 0.006 | 0.059 | -0.011 | 0.008 | 0.135 | -0.026 | 0.011 | 2.21×10^-02^ |
| rs2503099 | 6 | 100610101 | 0.84 | A | G | -0.034 | 0.003 | 6.37×10^-27^ | 0.009 | 0.009 | 0.302 | 0.005 | 0.011 | 0.671 | -0.027 | 0.017 | 0.102 |
| rs2547049 | 19 | 37482817 | 0.53 | A | G | 0.016 | 0.002 | 5.78×10^-11^ | -0.011 | 0.006 | 0.061 | -0.019 | 0.007 | 9.81×10^-03^ | 0.003 | 0.011 | 0.801 |
| rs2645294 | 1 | 119574587 | 0.56 | T | C | 0.027 | 0.002 | 1.14×10^-31^ | 0.006 | 0.006 | 0.319 | 0.005 | 0.008 | 0.505 | 0.016 | 0.012 | 0.159 |
| rs2725371 | 8 | 30854033 | 0.30 | A | G | 0.018 | 0.003 | 4.06×10^-10^ | 0.002 | 0.007 | 0.773 | 0.012 | 0.008 | 0.161 | 0.007 | 0.013 | 0.586 |
| rs2747399 | 20 | 51707666 | 0.49 | A | G | -0.023 | 0.002 | 8.45×10^-23^ | 0.007 | 0.006 | 0.288 | 0.005 | 0.008 | 0.526 | 0.018 | 0.012 | 0.131 |
| rs2791550 | 1 | 219655369 | 0.25 | T | G | -0.046 | 0.002 | 9.92×10^-80^ | -0.013 | 0.007 | 4.19×10^-02^ | -0.020 | 0.008 | 1.32×10^-02^ | 0.014 | 0.013 | 0.266 |
| rs28445639 | 2 | 25182488 | 0.21 | T | C | 0.027 | 0.003 | 5.73×10^-17^ | -0.036 | 0.008 | 1.72×10^-06^ | -0.032 | 0.009 | 7.24×10^-04^ | -0.055 | 0.014 | 1.39×10^-04^ |
| rs2894204 | 6 | 31237061 | 0.64 | T | C | 0.024 | 0.003 | 1.42×10^-17^ | 0.006 | 0.007 | 0.364 | 0.003 | 0.008 | 0.682 | 0.015 | 0.012 | 0.227 |
| rs2907794 | 10 | 32391109 | 0.25 | A | G | -0.016 | 0.003 | 4.47×10^-09^ | -0.003 | 0.007 | 0.686 | 0.002 | 0.009 | 0.857 | -0.007 | 0.014 | 0.623 |
| rs2925979 | 16 | 81534790 | 0.30 | T | C | 0.038 | 0.003 | 1.22×10^-51^ | -0.007 | 0.007 | 0.270 | -0.002 | 0.008 | 0.827 | 0.001 | 0.012 | 0.963 |
| rs3092781 | 20 | 45789953 | 0.45 | T | C | -0.014 | 0.002 | 4.75×10^-09^ | 0.003 | 0.006 | 0.594 | 0.017 | 0.008 | 3.08×10^-02^ | -0.019 | 0.012 | 0.108 |
| rs3213849 | 8 | 38326046 | 0.40 | A | G | 0.020 | 0.003 | 1.72×10^-13^ | -0.007 | 0.006 | 0.256 | -0.013 | 0.008 | 0.089 | 0.002 | 0.012 | 0.878 |
| rs34000 | 5 | 141973501 | 0.60 | T | C | 0.016 | 0.003 | 4.02×10^-11^ | 0.003 | 0.007 | 0.638 | 0.004 | 0.009 | 0.666 | 0.014 | 0.013 | 0.294 |
| rs362275 | 4 | 3224602 | 0.30 | T | C | -0.016 | 0.003 | 5.37×10^-10^ | -0.011 | 0.007 | 0.099 | -0.008 | 0.008 | 0.318 | -0.003 | 0.012 | 0.814 |
| rs3767846 | 1 | 214175126 | 0.75 | A | G | 0.020 | 0.003 | 5.69×10^-11^ | -0.023 | 0.007 | 1.87×10^-03^ | -0.024 | 0.009 | 1.19×10^-02^ | -0.033 | 0.014 | 1.86×10^-02^ |
| rs3786897 | 19 | 33893008 | 0.58 | A | G | -0.023 | 0.002 | 2.14×10^-23^ | 0.009 | 0.006 | 0.149 | 0.011 | 0.008 | 0.153 | 0.003 | 0.012 | 0.816 |
| rs3792751 | 5 | 32773314 | 0.36 | T | C | 0.014 | 0.002 | 3.90×10^-09^ | 0.005 | 0.007 | 0.445 | 0.006 | 0.008 | 0.450 | 0.008 | 0.012 | 0.509 |
| rs3803042 | 12 | 54387947 | 0.43 | A | G | 0.026 | 0.002 | 2.09×10^-27^ | -0.014 | 0.006 | 2.50×10^-02^ | -0.016 | 0.008 | 0.052 | 0.001 | 0.012 | 0.909 |
| rs3809924 | 18 | 46889938 | 0.35 | A | G | 0.018 | 0.003 | 6.24×10^-13^ | -0.008 | 0.006 | 0.230 | -0.006 | 0.008 | 0.431 | 0.004 | 0.012 | 0.749 |
| rs3851294 | 1 | 205130413 | 0.10 | A | G | -0.032 | 0.004 | 8.46×10^-15^ | -0.004 | 0.011 | 0.673 | 0.003 | 0.013 | 0.809 | -0.005 | 0.020 | 0.816 |
| rs3909256 | 9 | 96889505 | 0.75 | T | C | 0.017 | 0.003 | 2.62×10^-09^ | 0.000 | 0.007 | 0.968 | -0.003 | 0.009 | 0.728 | 0.030 | 0.014 | 2.78×10^-02^ |
| rs3930017 | 7 | 76720582 | 0.39 | A | G | -0.015 | 0.002 | 5.92×10^-10^ | 0.007 | 0.007 | 0.312 | -0.001 | 0.008 | 0.878 | 0.011 | 0.013 | 0.403 |
| rs39312 | 7 | 116954785 | 0.62 | A | C | -0.017 | 0.002 | 9.65×10^-13^ | 0.001 | 0.006 | 0.923 | -0.009 | 0.008 | 0.223 | 0.003 | 0.012 | 0.819 |
| rs3936510 | 5 | 55860866 | 0.20 | T | G | 0.042 | 0.003 | 2.59×10^-48^ | -0.020 | 0.008 | 1.18×10^-02^ | -0.030 | 0.010 | 2.25×10^-03^ | -0.014 | 0.015 | 0.338 |
| rs4130827 | 12 | 66430452 | 0.71 | T | G | -0.020 | 0.003 | 1.93×10^-13^ | 0.003 | 0.007 | 0.712 | 0.000 | 0.009 | 0.976 | 0.013 | 0.014 | 0.363 |
| rs4146819 | 16 | 69923563 | 0.58 | A | C | 0.018 | 0.003 | 1.19×10^-12^ | 0.011 | 0.006 | 0.077 | 0.010 | 0.008 | 0.182 | 0.023 | 0.012 | 4.66×10^-02^ |
| rs4346064 | 12 | 107081599 | 0.35 | A | C | 0.018 | 0.003 | 5.69×10^-11^ | -0.003 | 0.006 | 0.613 | -0.004 | 0.008 | 0.602 | -0.008 | 0.012 | 0.505 |
| rs4420638 | 19 | 45422946 | 0.81 | A | G | 0.033 | 0.003 | 9.81×10^-25^ | 0.014 | 0.008 | 0.097 | 0.019 | 0.010 | 0.064 | 0.025 | 0.016 | 0.120 |
| rs4465809 | 2 | 48980507 | 0.49 | T | G | 0.020 | 0.002 | 4.33×10^-17^ | 0.003 | 0.006 | 0.638 | 0.012 | 0.008 | 0.116 | -0.001 | 0.012 | 0.961 |
| rs4536164 | 11 | 27467109 | 0.31 | A | C | 0.015 | 0.003 | 1.47×10^-09^ | 0.003 | 0.007 | 0.683 | 0.000 | 0.008 | 0.988 | -0.001 | 0.013 | 0.923 |
| rs4556142 | 9 | 107734714 | 0.38 | A | G | -0.022 | 0.002 | 9.09×10^-20^ | 0.005 | 0.006 | 0.401 | 0.005 | 0.008 | 0.533 | -0.001 | 0.012 | 0.939 |
| rs4671193 | 2 | 67846288 | 0.36 | T | C | -0.022 | 0.003 | 5.77×10^-19^ | -0.015 | 0.006 | 1.67×10^-02^ | -0.008 | 0.008 | 0.296 | -0.017 | 0.012 | 0.147 |
| rs4705986 | 5 | 132349654 | 0.94 | T | G | 0.033 | 0.005 | 1.01×10^-10^ | 0.034 | 0.014 | 1.48×10^-02^ | 0.049 | 0.017 | 4.47×10^-03^ | -0.018 | 0.027 | 0.507 |
| rs474513 | 6 | 160770312 | 0.51 | A | G | 0.019 | 0.002 | 5.22×10^-16^ | -0.005 | 0.006 | 0.398 | -0.011 | 0.007 | 0.148 | -0.004 | 0.011 | 0.749 |
| rs4809604 | 20 | 45550489 | 0.58 | T | G | -0.026 | 0.002 | 3.17×10^-26^ | 0.000 | 0.006 | 0.955 | -0.004 | 0.008 | 0.597 | 0.008 | 0.012 | 0.523 |
| rs4871958 | 8 | 25762216 | 0.51 | A | G | 0.016 | 0.002 | 6.87×10^-13^ | 0.001 | 0.006 | 0.888 | -0.001 | 0.008 | 0.894 | 0.001 | 0.012 | 0.933 |
| rs4894803 | 3 | 171800256 | 0.60 | A | G | 0.018 | 0.002 | 2.33×10^-13^ | 0.011 | 0.007 | 0.079 | 0.009 | 0.008 | 0.275 | 0.013 | 0.012 | 0.300 |
| rs4929927 | 11 | 8658485 | 0.33 | A | G | -0.018 | 0.002 | 1.79×10^-14^ | -0.002 | 0.006 | 0.748 | -0.001 | 0.008 | 0.901 | -0.005 | 0.012 | 0.688 |
| rs4964656 | 12 | 108594069 | 0.30 | C | G | -0.020 | 0.003 | 1.22×10^-14^ | 0.000 | 0.008 | 0.947 | -0.002 | 0.010 | 0.854 | 0.009 | 0.014 | 0.539 |
| rs543874 | 1 | 177889480 | 0.78 | A | G | -0.019 | 0.003 | 9.65×10^-12^ | 0.034 | 0.008 | 1.00×10^-05^ | 0.025 | 0.009 | 7.28×10^-03^ | 0.014 | 0.014 | 0.327 |
| rs55747707 | 7 | 73037366 | 0.20 | A | G | -0.028 | 0.003 | 9.67×10^-17^ | 0.026 | 0.008 | 1.52×10^-03^ | 0.031 | 0.011 | 3.49×10^-03^ | 0.017 | 0.016 | 0.284 |
| rs55920843 | 2 | 158412701 | 0.99 | T | G | 0.097 | 0.013 | 1.69×10^-14^ | -0.046 | 0.032 | 0.159 | -0.049 | 0.041 | 0.227 | -0.154 | 0.061 | 1.23×10^-02^ |
| rs56271783 | 11 | 64004723 | 0.04 | C | G | 0.076 | 0.007 | 2.99×10^-31^ | -0.021 | 0.015 | 0.160 | 0.005 | 0.018 | 0.783 | -0.038 | 0.029 | 0.195 |
| rs579682 | 11 | 122014110 | 0.71 | T | C | -0.016 | 0.003 | 3.69×10^-09^ | -0.010 | 0.007 | 0.146 | -0.010 | 0.008 | 0.208 | -0.018 | 0.013 | 0.161 |
| rs6021889 | 20 | 50982870 | 0.71 | A | G | 0.021 | 0.003 | 2.59×10^-17^ | 0.013 | 0.007 | 0.064 | 0.017 | 0.009 | 4.65×10^-02^ | 0.017 | 0.013 | 0.215 |
| rs605066 | 6 | 139829666 | 0.58 | T | C | -0.030 | 0.002 | 5.73×10^-35^ | 0.000 | 0.006 | 0.970 | -0.005 | 0.008 | 0.496 | 0.015 | 0.012 | 0.192 |
| rs6090040 | 20 | 62692060 | 0.48 | A | C | 0.021 | 0.003 | 9.03×10^-15^ | -0.028 | 0.006 | 9.19×10^-06^ | -0.031 | 0.008 | 6.21×10^-05^ | -0.015 | 0.012 | 0.201 |
| rs62106258 | 2 | 417167 | 0.95 | T | C | 0.053 | 0.006 | 4.18×10^-17^ | -0.006 | 0.017 | 0.715 | -0.032 | 0.021 | 0.131 | -0.013 | 0.033 | 0.700 |
| rs62271373 | 3 | 150066540 | 0.06 | A | T | 0.057 | 0.006 | 1.03×10^-22^ | -0.011 | 0.015 | 0.463 | -0.013 | 0.020 | 0.509 | 0.000 | 0.029 | 0.992 |
| rs645040 | 3 | 135926622 | 0.77 | T | G | 0.018 | 0.003 | 3.18×10^-11^ | -0.016 | 0.007 | 3.03×10^-02^ | -0.011 | 0.009 | 0.239 | -0.026 | 0.014 | 0.055 |
| rs6542479 | 2 | 119541788 | 0.35 | C | G | 0.018 | 0.003 | 6.76×10^-14^ | 0.002 | 0.006 | 0.764 | 0.004 | 0.008 | 0.593 | 0.002 | 0.012 | 0.846 |
| rs6688233 | 1 | 9335745 | 0.24 | T | C | 0.025 | 0.003 | 1.12×10^-19^ | 0.005 | 0.008 | 0.536 | 0.015 | 0.010 | 0.118 | -0.001 | 0.015 | 0.960 |
| rs6691427 | 1 | 203510048 | 0.49 | C | G | -0.017 | 0.003 | 1.56×10^-10^ | 0.009 | 0.007 | 0.197 | 0.014 | 0.009 | 0.107 | 0.004 | 0.014 | 0.790 |
| rs6699397 | 1 | 91212216 | 0.62 | A | G | -0.015 | 0.002 | 3.00×10^-10^ | 0.000 | 0.007 | 0.943 | -0.005 | 0.008 | 0.585 | 0.004 | 0.013 | 0.781 |
| rs6719428 | 2 | 66238005 | 0.70 | T | C | -0.022 | 0.003 | 1.11×10^-17^ | 0.004 | 0.007 | 0.576 | 0.009 | 0.008 | 0.283 | 0.005 | 0.013 | 0.679 |
| rs6725549 | 2 | 630323 | 0.84 | A | C | 0.024 | 0.003 | 6.64×10^-15^ | -0.031 | 0.008 | 6.75×10^-05^ | -0.026 | 0.010 | 7.00×10^-03^ | -0.072 | 0.015 | 1.18×10^-06^ |
| rs6795831 | 3 | 129341403 | 0.82 | A | C | 0.044 | 0.003 | 1.33×10^-49^ | -0.017 | 0.008 | 2.83×10^-02^ | -0.026 | 0.010 | 7.13×10^-03^ | -0.016 | 0.015 | 0.282 |
| rs6905288 | 6 | 43758873 | 0.57 | A | G | 0.050 | 0.002 | 1.55×10^-98^ | -0.020 | 0.006 | 1.15×10^-03^ | -0.024 | 0.008 | 2.10×10^-03^ | -0.004 | 0.012 | 0.709 |
| rs7020604 | 9 | 112583554 | 0.66 | A | G | 0.017 | 0.003 | 8.92×10^-10^ | -0.010 | 0.007 | 0.128 | -0.009 | 0.008 | 0.279 | 0.000 | 0.012 | 0.975 |
| rs703984 | 10 | 80941417 | 0.41 | C | G | -0.015 | 0.002 | 1.19×10^-09^ | 0.012 | 0.006 | 4.76×10^-02^ | 0.011 | 0.008 | 0.170 | 0.029 | 0.012 | 1.15×10^-02^ |
| rs704067 | 12 | 89726027 | 0.45 | A | G | 0.015 | 0.002 | 1.77×10^-09^ | -0.004 | 0.007 | 0.522 | -0.005 | 0.009 | 0.570 | 0.008 | 0.013 | 0.559 |
| rs7102 | 16 | 11642242 | 0.65 | T | C | -0.015 | 0.002 | 2.59×10^-10^ | -0.004 | 0.006 | 0.533 | 0.002 | 0.008 | 0.769 | -0.008 | 0.012 | 0.529 |
| rs711869 | 2 | 13073967 | 0.56 | A | G | -0.016 | 0.002 | 1.11×10^-11^ | 0.003 | 0.006 | 0.631 | 0.000 | 0.008 | 0.983 | -0.007 | 0.012 | 0.535 |
| rs7175346 | 15 | 42100674 | 0.52 | A | G | 0.016 | 0.002 | 6.55×10^-11^ | 0.000 | 0.006 | 0.942 | 0.005 | 0.008 | 0.542 | -0.009 | 0.012 | 0.452 |
| rs7213608 | 17 | 21279289 | 0.68 | T | C | -0.019 | 0.003 | 1.68×10^-13^ | -0.004 | 0.006 | 0.553 | 0.000 | 0.008 | 0.977 | 0.006 | 0.012 | 0.625 |
| rs7221005 | 17 | 67590431 | 0.33 | T | C | 0.016 | 0.003 | 1.64×10^-09^ | 0.000 | 0.007 | 0.976 | 0.007 | 0.008 | 0.402 | 0.001 | 0.012 | 0.963 |
| rs7231852 | 18 | 40761976 | 0.25 | A | G | -0.017 | 0.003 | 1.68×10^-10^ | -0.005 | 0.007 | 0.449 | -0.013 | 0.009 | 0.135 | 0.009 | 0.013 | 0.495 |
| rs7249081 | 19 | 2157167 | 0.52 | T | C | 0.017 | 0.003 | 2.28×10^-10^ | -0.011 | 0.006 | 0.081 | -0.014 | 0.007 | 0.055 | -0.025 | 0.011 | 2.92×10^-02^ |
| rs727428 | 17 | 7537792 | 0.45 | T | C | 0.014 | 0.002 | 4.34×10^-09^ | 0.002 | 0.006 | 0.760 | 0.001 | 0.007 | 0.884 | 0.001 | 0.011 | 0.907 |
| rs72959041 | 6 | 127454893 | 0.05 | A | G | 0.179 | 0.006 | 1.70×10^-175^ | -0.059 | 0.015 | 9.44×10^-05^ | -0.072 | 0.019 | 1.12×10^-04^ | -0.008 | 0.029 | 0.785 |
| rs7395513 | 11 | 69262756 | 0.44 | A | G | -0.024 | 0.003 | 8.58×10^-19^ | -0.037 | 0.006 | 1.16×10^-09^ | -0.040 | 0.007 | 5.75×10^-08^ | 0.003 | 0.011 | 0.791 |
| rs7402977 | 15 | 31726824 | 0.26 | A | G | -0.017 | 0.003 | 1.53×10^-09^ | 0.012 | 0.007 | 0.095 | 0.019 | 0.009 | 2.41×10^-02^ | 0.024 | 0.013 | 0.064 |
| rs747249 | 11 | 130271647 | 0.36 | A | G | 0.015 | 0.003 | 9.22×10^-10^ | -0.004 | 0.007 | 0.602 | -0.011 | 0.009 | 0.193 | 0.016 | 0.013 | 0.218 |
| rs7492628 | 14 | 91547136 | 0.68 | C | G | -0.019 | 0.003 | 9.03×10^-14^ | 0.005 | 0.007 | 0.447 | 0.001 | 0.008 | 0.930 | 0.031 | 0.012 | 1.18×10^-02^ |
| rs7498665 | 16 | 28883241 | 0.62 | A | G | -0.014 | 0.002 | 1.47×10^-09^ | -0.005 | 0.006 | 0.428 | -0.009 | 0.008 | 0.216 | 0.006 | 0.012 | 0.613 |
| rs7585974 | 2 | 172377212 | 0.18 | C | G | 0.022 | 0.003 | 1.51×10^-10^ | 0.023 | 0.008 | 3.52×10^-03^ | 0.025 | 0.010 | 8.86×10^-03^ | 0.008 | 0.015 | 0.601 |
| rs7607980 | 2 | 165551201 | 0.87 | T | C | 0.060 | 0.004 | 1.65×10^-66^ | -0.011 | 0.009 | 0.221 | -0.014 | 0.011 | 0.209 | 0.022 | 0.017 | 0.187 |
| rs7612999 | 3 | 35678337 | 0.25 | A | G | 0.021 | 0.003 | 2.52×10^-14^ | 0.002 | 0.007 | 0.785 | 0.005 | 0.009 | 0.578 | 0.011 | 0.013 | 0.417 |
| rs76189032 | 17 | 40756882 | 0.08 | T | C | 0.037 | 0.005 | 2.33×10^-13^ | 0.038 | 0.012 | 1.32×10^-03^ | 0.049 | 0.015 | 7.62×10^-04^ | 0.038 | 0.023 | 0.092 |
| rs7637773 | 3 | 185515635 | 0.32 | A | G | 0.015 | 0.003 | 1.56×10^-09^ | -0.015 | 0.006 | 2.06×10^-02^ | -0.022 | 0.008 | 6.51×10^-03^ | -0.013 | 0.012 | 0.292 |
| rs7731717 | 5 | 173281685 | 0.70 | T | C | -0.025 | 0.003 | 2.71×10^-17^ | 0.022 | 0.006 | 5.53×10^-04^ | 0.020 | 0.008 | 1.28×10^-02^ | 0.024 | 0.012 | 4.76×10^-02^ |
| rs7736177 | 5 | 55003944 | 0.69 | A | G | -0.017 | 0.003 | 1.96×10^-11^ | -0.006 | 0.006 | 0.348 | -0.003 | 0.008 | 0.738 | 0.003 | 0.012 | 0.825 |
| rs7783857 | 7 | 130439058 | 0.73 | C | G | -0.024 | 0.003 | 1.92×10^-21^ | 0.022 | 0.007 | 1.80×10^-03^ | 0.026 | 0.009 | 2.76×10^-03^ | 0.008 | 0.013 | 0.552 |
| rs7932891 | 11 | 10921512 | 0.30 | A | G | 0.016 | 0.003 | 2.60×10^-09^ | -0.016 | 0.007 | 3.40×10^-02^ | -0.020 | 0.009 | 3.24×10^-02^ | -0.007 | 0.014 | 0.611 |
| rs797486 | 13 | 51221618 | 0.89 | A | C | 0.036 | 0.004 | 7.66×10^-25^ | -0.010 | 0.009 | 0.249 | -0.011 | 0.011 | 0.317 | 0.002 | 0.017 | 0.918 |
| rs8033381 | 15 | 75080685 | 0.73 | A | G | 0.016 | 0.003 | 9.67×10^-10^ | 0.029 | 0.007 | 1.19×10^-05^ | 0.037 | 0.008 | 9.67×10^-06^ | 0.024 | 0.013 | 0.059 |
| rs8054299 | 16 | 53498655 | 0.69 | C | G | 0.017 | 0.003 | 8.79×10^-11^ | -0.008 | 0.006 | 0.210 | -0.013 | 0.008 | 0.102 | 0.018 | 0.012 | 0.128 |
| rs8054556 | 16 | 29958216 | 0.46 | A | G | 0.020 | 0.003 | 9.91×10^-14^ | 0.002 | 0.006 | 0.800 | -0.004 | 0.008 | 0.554 | 0.014 | 0.011 | 0.232 |
| rs8070723 | 17 | 44081064 | 0.78 | A | G | -0.022 | 0.003 | 3.19×10^-15^ | 0.045 | 0.007 | 9.43×10^-10^ | 0.044 | 0.009 | 1.24×10^-06^ | 0.040 | 0.014 | 4.39×10^-03^ |
| rs8078513 | 17 | 17431208 | 0.05 | A | C | 0.039 | 0.005 | 6.35×10^-13^ | -0.011 | 0.013 | 0.389 | -0.034 | 0.016 | 4.03×10^-02^ | -0.039 | 0.025 | 0.120 |
| rs8079062 | 17 | 74255029 | 0.92 | A | G | -0.034 | 0.004 | 6.51×10^-15^ | 0.007 | 0.012 | 0.572 | 0.002 | 0.015 | 0.914 | -0.001 | 0.022 | 0.964 |
| rs8103017 | 19 | 55999142 | 0.70 | C | G | -0.019 | 0.003 | 6.49×10^-11^ | -0.019 | 0.007 | 8.94×10^-03^ | -0.021 | 0.009 | 1.87×10^-02^ | -0.022 | 0.014 | 0.102 |
| rs863750 | 12 | 124505444 | 0.58 | T | C | 0.041 | 0.002 | 3.97×10^-68^ | -0.016 | 0.006 | 7.27×10^-03^ | -0.016 | 0.008 | 3.07×10^-02^ | -0.026 | 0.012 | 2.80×10^-02^ |
| rs905938 | 1 | 154991389 | 0.72 | T | C | 0.021 | 0.003 | 3.90×10^-16^ | 0.020 | 0.007 | 4.31×10^-03^ | 0.021 | 0.009 | 1.55×10^-02^ | 0.008 | 0.013 | 0.526 |
| rs917191 | 7 | 80570871 | 0.57 | C | G | 0.017 | 0.002 | 1.40×10^-12^ | -0.005 | 0.007 | 0.463 | 0.004 | 0.008 | 0.639 | -0.015 | 0.013 | 0.223 |
| rs9296938 | 6 | 14573063 | 0.25 | A | G | -0.017 | 0.003 | 2.94×10^-10^ | 0.011 | 0.007 | 0.116 | 0.009 | 0.009 | 0.309 | 0.001 | 0.013 | 0.915 |
| rs9352872 | 6 | 81510118 | 0.53 | C | G | -0.021 | 0.003 | 8.55×10^-16^ | 0.020 | 0.006 | 8.17×10^-04^ | 0.015 | 0.007 | 4.10×10^-02^ | 0.014 | 0.011 | 0.233 |
| rs9436299 | 1 | 65892888 | 0.66 | A | C | 0.016 | 0.003 | 6.84×10^-10^ | -0.006 | 0.007 | 0.387 | 0.005 | 0.008 | 0.517 | -0.007 | 0.012 | 0.568 |
| rs9644033 | 8 | 23610639 | 0.75 | A | T | 0.033 | 0.003 | 2.89×10^-33^ | -0.009 | 0.007 | 0.175 | -0.007 | 0.009 | 0.409 | -0.013 | 0.013 | 0.341 |
| rs9674436 | 16 | 1342841 | 0.47 | A | G | 0.016 | 0.003 | 2.14×10^-09^ | 0.009 | 0.006 | 0.158 | 0.008 | 0.008 | 0.303 | -0.003 | 0.012 | 0.820 |
| rs9678859 | 2 | 100288478 | 0.18 | A | G | 0.021 | 0.003 | 2.90×10^-11^ | 0.000 | 0.008 | 0.965 | 0.006 | 0.010 | 0.546 | -0.009 | 0.015 | 0.546 |
| rs974801 | 4 | 106071064 | 0.64 | A | G | -0.017 | 0.002 | 4.18×10^-13^ | -0.022 | 0.006 | 2.60×10^-04^ | -0.033 | 0.008 | 1.01×10^-05^ | 0.011 | 0.012 | 0.335 |
| rs9991328 | 4 | 89713121 | 0.47 | T | C | 0.033 | 0.002 | 1.18×10^-48^ | 0.008 | 0.006 | 0.160 | 0.000 | 0.007 | 0.953 | 0.015 | 0.011 | 0.194 |
| **WHR_adj_**BMI **(female)** | |  |  |  |  |  |  |  |  |  |  |  |  |  |  |  |  |
| rs10019888 | 4 | 26062990 | 0.81 | A | G | -0.030 | 0.003 | 2.04×10^-22^ | 0.000 | 0.008 | 0.962 | -0.016 | 0.010 | 0.121 | 0.008 | 0.016 | 0.610 |
| rs10054063 | 5 | 173392398 | 0.70 | A | T | -0.032 | 0.003 | 7.13×10^-28^ | 0.025 | 0.007 | 1.41×10^-04^ | 0.027 | 0.008 | 7.80×10^-04^ | 0.032 | 0.012 | 1.01×10^-02^ |
| rs10101067 | 8 | 72407374 | 0.07 | C | G | 0.054 | 0.005 | 5.67×10^-33^ | -0.019 | 0.012 | 0.128 | -0.030 | 0.015 | 4.98×10^-02^ | -0.024 | 0.023 | 0.313 |
| rs1029472 | 5 | 130867733 | 0.94 | A | G | -0.035 | 0.006 | 3.96×10^-10^ | 0.031 | 0.012 | 8.48×10^-03^ | 0.045 | 0.015 | 2.33×10^-03^ | -0.012 | 0.022 | 0.601 |
| rs1035942 | 19 | 7199803 | 0.27 | A | G | 0.023 | 0.003 | 2.25×10^-17^ | -0.003 | 0.007 | 0.622 | 0.004 | 0.008 | 0.650 | -0.002 | 0.013 | 0.905 |
| rs10401969 | 19 | 19407718 | 0.92 | T | C | -0.026 | 0.004 | 4.47×10^-09^ | -0.019 | 0.011 | 0.082 | -0.023 | 0.014 | 0.098 | -0.020 | 0.021 | 0.340 |
| rs1040848 | 6 | 109247307 | 0.76 | A | T | 0.019 | 0.003 | 7.14×10^-12^ | -0.010 | 0.007 | 0.155 | -0.003 | 0.009 | 0.713 | 0.003 | 0.013 | 0.795 |
| rs1045241 | 5 | 118729286 | 0.28 | T | C | -0.033 | 0.003 | 4.11×10^-38^ | 0.011 | 0.007 | 0.114 | 0.012 | 0.008 | 0.155 | -0.027 | 0.013 | 3.44×10^-02^ |
| rs10462028 | 4 | 56298300 | 0.32 | A | G | 0.028 | 0.003 | 5.01×10^-28^ | -0.010 | 0.006 | 0.108 | -0.011 | 0.008 | 0.173 | -0.021 | 0.012 | 0.082 |
| rs10463353 | 5 | 141710741 | 0.37 | T | C | -0.015 | 0.003 | 2.82×10^-09^ | 0.017 | 0.006 | 5.92×10^-03^ | 0.025 | 0.008 | 1.13×10^-03^ | 0.009 | 0.012 | 0.465 |
| rs10463416 | 5 | 148572915 | 0.36 | A | G | 0.015 | 0.002 | 1.73×10^-09^ | -0.002 | 0.006 | 0.753 | 0.009 | 0.008 | 0.242 | -0.013 | 0.012 | 0.252 |
| rs10494421 | 1 | 164738727 | 0.12 | T | C | 0.024 | 0.004 | 1.92×10^-11^ | -0.016 | 0.011 | 0.145 | -0.033 | 0.014 | 1.31×10^-02^ | 0.016 | 0.020 | 0.430 |
| rs1057119 | 14 | 23746269 | 0.77 | C | G | 0.018 | 0.003 | 2.97×10^-10^ | -0.001 | 0.008 | 0.879 | -0.005 | 0.009 | 0.577 | -0.007 | 0.014 | 0.617 |
| rs1073632 | 16 | 68248607 | 0.04 | A | G | -0.037 | 0.006 | 1.47×10^-10^ | 0.010 | 0.015 | 0.501 | -0.013 | 0.018 | 0.479 | 0.059 | 0.028 | 3.55×10^-02^ |
| rs10761604 | 10 | 63819903 | 0.44 | T | C | 0.020 | 0.002 | 3.13×10^-17^ | 0.003 | 0.006 | 0.661 | -0.003 | 0.008 | 0.730 | 0.023 | 0.012 | 4.89×10^-02^ |
| rs1077795 | 19 | 17222584 | 0.74 | A | G | 0.020 | 0.003 | 6.11×10^-11^ | -0.006 | 0.007 | 0.349 | -0.015 | 0.008 | 0.071 | 0.019 | 0.013 | 0.134 |
| rs10808546 | 8 | 126495818 | 0.42 | T | C | -0.025 | 0.002 | 3.37×10^-27^ | 0.013 | 0.006 | 3.47×10^-02^ | 0.014 | 0.008 | 0.067 | 0.015 | 0.012 | 0.201 |
| rs10820747 | 9 | 107686823 | 0.24 | A | G | 0.038 | 0.003 | 2.72×10^-34^ | 0.005 | 0.008 | 0.494 | -0.001 | 0.009 | 0.939 | 0.004 | 0.014 | 0.790 |
| rs10842707 | 12 | 26471364 | 0.20 | T | C | 0.048 | 0.003 | 4.48×10^-68^ | 0.005 | 0.007 | 0.506 | 0.010 | 0.009 | 0.274 | 0.019 | 0.014 | 0.156 |
| rs10843817 | 12 | 30865510 | 0.76 | A | C | 0.018 | 0.003 | 1.36×10^-10^ | -0.025 | 0.007 | 2.80×10^-04^ | -0.026 | 0.008 | 1.72×10^-03^ | 0.002 | 0.013 | 0.899 |
| rs10880823 | 12 | 45986738 | 0.76 | T | C | -0.023 | 0.003 | 5.99×10^-16^ | -0.009 | 0.008 | 0.252 | -0.010 | 0.010 | 0.310 | 0.000 | 0.015 | 0.997 |
| rs10887759 | 10 | 89603424 | 0.16 | A | G | 0.022 | 0.003 | 1.05×10^-11^ | 0.008 | 0.009 | 0.385 | 0.019 | 0.011 | 0.072 | -0.008 | 0.017 | 0.648 |
| rs10891290 | 11 | 111605215 | 0.64 | T | C | 0.028 | 0.002 | 2.24×10^-29^ | 0.010 | 0.006 | 0.098 | 0.012 | 0.008 | 0.108 | -0.005 | 0.012 | 0.685 |
| rs10919388 | 1 | 170372503 | 0.27 | A | C | -0.044 | 0.003 | 2.13×10^-63^ | -0.008 | 0.007 | 0.248 | -0.009 | 0.009 | 0.315 | -0.031 | 0.013 | 1.85×10^-02^ |
| rs10946205 | 6 | 167451311 | 0.53 | A | G | -0.014 | 0.002 | 2.85×10^-09^ | 0.008 | 0.006 | 0.206 | 0.004 | 0.008 | 0.573 | 0.015 | 0.012 | 0.205 |
| rs10980797 | 9 | 113912553 | 0.52 | A | G | -0.024 | 0.003 | 2.74×10^-19^ | 0.009 | 0.006 | 0.126 | 0.012 | 0.008 | 0.119 | 0.015 | 0.011 | 0.189 |
| rs10992447 | 9 | 95509733 | 0.16 | T | C | 0.024 | 0.003 | 3.35×10^-14^ | 0.006 | 0.009 | 0.476 | 0.005 | 0.011 | 0.658 | 0.005 | 0.017 | 0.750 |
| rs11006228 | 10 | 60460025 | 0.20 | A | G | 0.020 | 0.003 | 3.40×10^-09^ | -0.002 | 0.008 | 0.795 | -0.010 | 0.011 | 0.374 | -0.015 | 0.016 | 0.364 |
| rs11045172 | 12 | 20470221 | 0.81 | A | C | 0.019 | 0.003 | 9.22×10^-10^ | -0.012 | 0.008 | 0.108 | -0.007 | 0.010 | 0.456 | 0.002 | 0.015 | 0.909 |
| rs11082430 | 18 | 42752377 | 0.74 | C | G | 0.017 | 0.003 | 1.16×10^-09^ | 0.030 | 0.007 | 1.79×10^-05^ | 0.025 | 0.009 | 4.44×10^-03^ | 0.019 | 0.013 | 0.158 |
| rs11118610 | 1 | 220990086 | 0.57 | A | C | 0.014 | 0.002 | 3.15×10^-09^ | -0.002 | 0.006 | 0.692 | 0.002 | 0.008 | 0.838 | 0.009 | 0.012 | 0.429 |
| rs11129657 | 3 | 35636709 | 0.79 | T | C | -0.019 | 0.003 | 2.16×10^-10^ | -0.005 | 0.007 | 0.497 | -0.009 | 0.009 | 0.336 | -0.011 | 0.014 | 0.432 |
| rs11187537 | 10 | 95346805 | 0.26 | C | G | 0.026 | 0.003 | 9.55×10^-21^ | 0.008 | 0.007 | 0.255 | 0.008 | 0.009 | 0.392 | 0.008 | 0.014 | 0.545 |
| rs11204762 | 1 | 150999737 | 0.77 | A | G | 0.018 | 0.003 | 4.00×10^-10^ | -0.006 | 0.008 | 0.455 | -0.016 | 0.009 | 0.088 | 0.008 | 0.014 | 0.575 |
| rs11235 | 3 | 52745087 | 0.54 | T | C | -0.024 | 0.002 | 1.96×10^-25^ | -0.010 | 0.006 | 0.101 | -0.013 | 0.008 | 0.084 | 0.000 | 0.012 | 0.991 |
| rs112907088 | 5 | 142083969 | 0.98 | A | G | -0.053 | 0.009 | 7.65×10^-10^ | 0.056 | 0.023 | 1.34×10^-02^ | 0.056 | 0.029 | 0.052 | -0.004 | 0.043 | 0.920 |
| rs1139653 | 16 | 4484396 | 0.29 | A | T | 0.020 | 0.003 | 1.88×10^-14^ | -0.002 | 0.007 | 0.836 | -0.008 | 0.009 | 0.425 | -0.007 | 0.014 | 0.639 |
| rs114760566 | 6 | 34192036 | 0.04 | A | C | 0.121 | 0.007 | 4.36×10^-77^ | -0.018 | 0.016 | 0.247 | -0.024 | 0.019 | 0.213 | -0.021 | 0.030 | 0.492 |
| rs11592754 | 10 | 32619572 | 0.86 | A | C | -0.020 | 0.003 | 1.57×10^-09^ | -0.018 | 0.009 | 4.57×10^-02^ | -0.022 | 0.011 | 4.22×10^-02^ | -0.025 | 0.017 | 0.138 |
| rs11603150 | 11 | 36373869 | 0.69 | T | C | 0.023 | 0.003 | 1.94×10^-15^ | 0.001 | 0.006 | 0.936 | 0.010 | 0.008 | 0.203 | -0.015 | 0.012 | 0.216 |
| rs11605956 | 11 | 26212866 | 0.69 | T | C | 0.019 | 0.003 | 1.28×10^-10^ | -0.006 | 0.007 | 0.380 | -0.009 | 0.008 | 0.263 | -0.008 | 0.012 | 0.522 |
| rs11668242 | 19 | 40836872 | 0.29 | A | G | -0.016 | 0.003 | 3.08×10^-09^ | -0.012 | 0.007 | 0.074 | -0.008 | 0.008 | 0.337 | -0.004 | 0.013 | 0.747 |
| rs116911572 | 17 | 943593 | 0.98 | T | C | 0.062 | 0.010 | 2.62×10^-09^ | -0.005 | 0.027 | 0.842 | -0.022 | 0.034 | 0.523 | 0.007 | 0.053 | 0.894 |
| rs11694173 | 2 | 43590899 | 0.18 | A | G | 0.022 | 0.003 | 6.75×10^-14^ | 0.011 | 0.008 | 0.163 | 0.018 | 0.009 | 4.99×10^-02^ | 0.013 | 0.014 | 0.352 |
| rs11716727 | 3 | 12489012 | 0.26 | T | C | 0.035 | 0.003 | 3.24×10^-30^ | 0.014 | 0.007 | 4.44×10^-02^ | 0.012 | 0.009 | 0.158 | 0.004 | 0.014 | 0.753 |
| rs11722554 | 4 | 5016883 | 0.04 | A | G | -0.040 | 0.007 | 1.42×10^-09^ | 0.001 | 0.019 | 0.958 | -0.007 | 0.024 | 0.780 | -0.010 | 0.036 | 0.789 |
| rs11727676 | 4 | 145659064 | 0.91 | T | C | -0.034 | 0.004 | 2.74×10^-16^ | 0.012 | 0.011 | 0.297 | 0.013 | 0.014 | 0.328 | -0.017 | 0.021 | 0.410 |
| rs11756568 | 6 | 152042413 | 0.74 | A | T | -0.016 | 0.003 | 3.67×10^-09^ | -0.048 | 0.007 | 3.45×10^-13^ | -0.035 | 0.008 | 2.00×10^-05^ | -0.072 | 0.013 | 7.24×10^-09^ |
| rs11770285 | 7 | 107616011 | 0.90 | C | G | 0.036 | 0.004 | 8.46×10^-19^ | 0.008 | 0.011 | 0.485 | 0.005 | 0.014 | 0.752 | 0.016 | 0.022 | 0.451 |
| rs11992444 | 8 | 25464690 | 0.51 | T | G | 0.022 | 0.003 | 7.29×10^-16^ | -0.003 | 0.008 | 0.658 | -0.003 | 0.010 | 0.781 | 0.026 | 0.015 | 0.081 |
| rs12061508 | 1 | 196584468 | 0.21 | A | G | 0.018 | 0.003 | 2.34×10^-10^ | 0.002 | 0.007 | 0.823 | 0.003 | 0.009 | 0.739 | -0.004 | 0.014 | 0.759 |
| rs12138803 | 1 | 172348823 | 0.20 | T | C | 0.035 | 0.003 | 6.97×10^-40^ | -0.007 | 0.007 | 0.327 | 0.000 | 0.009 | 0.982 | -0.021 | 0.013 | 0.109 |
| rs12185233 | 17 | 43923654 | 0.21 | C | G | 0.021 | 0.003 | 5.04×10^-14^ | -0.044 | 0.007 | 2.21×10^-09^ | -0.044 | 0.009 | 1.29×10^-06^ | -0.038 | 0.014 | 5.91×10^-03^ |
| rs12192658 | 6 | 112504240 | 0.10 | A | G | -0.024 | 0.004 | 2.47×10^-09^ | -0.007 | 0.010 | 0.494 | 0.003 | 0.013 | 0.799 | -0.050 | 0.020 | 1.02×10^-02^ |
| rs12328675 | 2 | 165540800 | 0.87 | T | C | 0.076 | 0.004 | 8.92×10^-104^ | -0.012 | 0.009 | 0.170 | -0.015 | 0.011 | 0.181 | 0.023 | 0.017 | 0.177 |
| rs12340775 | 9 | 13226945 | 0.06 | A | G | -0.032 | 0.005 | 4.37×10^-09^ | -0.001 | 0.014 | 0.964 | 0.023 | 0.018 | 0.188 | -0.006 | 0.027 | 0.823 |
| rs12369214 | 12 | 107198611 | 0.39 | A | G | 0.020 | 0.003 | 3.99×10^-16^ | -0.004 | 0.006 | 0.557 | -0.005 | 0.008 | 0.499 | -0.009 | 0.012 | 0.462 |
| rs12441543 | 15 | 31689543 | 0.29 | A | G | -0.020 | 0.003 | 6.38×10^-15^ | 0.006 | 0.007 | 0.345 | 0.016 | 0.008 | 0.059 | 0.010 | 0.013 | 0.448 |
| rs12454712 | 18 | 60845884 | 0.62 | T | C | 0.031 | 0.003 | 1.96×10^-36^ | 0.007 | 0.006 | 0.233 | 0.010 | 0.008 | 0.213 | -0.016 | 0.012 | 0.191 |
| rs12496583 | 3 | 123121286 | 0.75 | A | G | -0.016 | 0.003 | 1.20×10^-09^ | 0.003 | 0.007 | 0.670 | -0.007 | 0.009 | 0.411 | 0.005 | 0.014 | 0.704 |
| rs1250259 | 2 | 216300482 | 0.74 | A | T | -0.022 | 0.003 | 1.04×10^-15^ | 0.003 | 0.007 | 0.685 | 0.007 | 0.009 | 0.424 | -0.002 | 0.013 | 0.877 |
| rs12543555 | 8 | 69562014 | 0.81 | A | G | -0.017 | 0.003 | 2.26×10^-09^ | -0.010 | 0.008 | 0.193 | -0.002 | 0.009 | 0.850 | -0.006 | 0.014 | 0.679 |
| rs12591294 | 15 | 56525994 | 0.78 | T | C | -0.027 | 0.003 | 1.86×10^-20^ | -0.001 | 0.008 | 0.868 | -0.003 | 0.010 | 0.757 | 0.020 | 0.015 | 0.193 |
| rs12675159 | 8 | 71945274 | 0.32 | A | G | 0.017 | 0.003 | 3.06×10^-09^ | 0.017 | 0.007 | 1.04×10^-02^ | 0.016 | 0.008 | 4.33×10^-02^ | 0.011 | 0.012 | 0.366 |
| rs12817549 | 12 | 94121314 | 0.55 | T | C | -0.016 | 0.002 | 1.05×10^-12^ | -0.012 | 0.006 | 0.056 | -0.009 | 0.008 | 0.247 | -0.009 | 0.011 | 0.410 |
| rs1294420 | 6 | 6742752 | 0.54 | T | C | 0.029 | 0.002 | 1.64×10^-37^ | 0.016 | 0.006 | 8.07×10^-03^ | 0.009 | 0.007 | 0.239 | 0.011 | 0.011 | 0.331 |
| rs12983546 | 19 | 2195065 | 0.47 | T | G | -0.018 | 0.003 | 4.53×10^-11^ | 0.017 | 0.006 | 4.91×10^-03^ | 0.024 | 0.007 | 1.29×10^-03^ | 0.023 | 0.011 | 4.41×10^-02^ |
| rs13107325 | 4 | 103188709 | 0.08 | T | C | -0.035 | 0.005 | 5.74×10^-14^ | 0.019 | 0.012 | 0.119 | 0.020 | 0.015 | 0.178 | -0.006 | 0.023 | 0.776 |
| rs1316979 | 6 | 41708664 | 0.07 | T | C | 0.029 | 0.005 | 5.46×10^-10^ | 0.023 | 0.013 | 0.065 | 0.009 | 0.016 | 0.554 | 0.030 | 0.024 | 0.207 |
| rs13223303 | 7 | 42692711 | 0.71 | T | C | 0.019 | 0.003 | 1.26×10^-10^ | 0.010 | 0.007 | 0.157 | 0.019 | 0.009 | 2.92×10^-02^ | 0.009 | 0.013 | 0.517 |
| rs13229464 | 7 | 32890237 | 0.32 | T | C | -0.017 | 0.003 | 2.37×10^-09^ | -0.006 | 0.007 | 0.414 | -0.009 | 0.009 | 0.316 | -0.022 | 0.013 | 0.094 |
| rs13232403 | 7 | 20398192 | 0.27 | A | G | -0.019 | 0.003 | 6.87×10^-10^ | -0.003 | 0.007 | 0.619 | -0.005 | 0.008 | 0.538 | -0.001 | 0.013 | 0.955 |
| rs13234914 | 7 | 84577411 | 0.66 | A | G | -0.016 | 0.003 | 6.38×10^-11^ | -0.009 | 0.006 | 0.150 | -0.015 | 0.008 | 0.059 | -0.016 | 0.012 | 0.189 |
| rs13256367 | 8 | 128334900 | 0.65 | A | C | 0.026 | 0.003 | 9.72×10^-26^ | -0.070 | 0.006 | 2.41×10^-29^ | -0.076 | 0.008 | 3.30×10^-23^ | -0.030 | 0.012 | 1.15×10^-02^ |
| rs13306567 | 1 | 11860465 | 0.95 | C | G | 0.030 | 0.005 | 4.69×10^-09^ | 0.036 | 0.014 | 7.50×10^-03^ | 0.040 | 0.017 | 1.63×10^-02^ | 0.047 | 0.026 | 0.070 |
| rs1334576 | 6 | 7211818 | 0.43 | A | G | -0.015 | 0.002 | 2.30×10^-10^ | -0.017 | 0.006 | 4.40×10^-03^ | -0.017 | 0.008 | 2.40×10^-02^ | -0.041 | 0.012 | 3.49×10^-04^ |
| rs13404250 | 2 | 188116196 | 0.33 | T | C | -0.024 | 0.003 | 3.44×10^-22^ | 0.014 | 0.006 | 2.82×10^-02^ | 0.023 | 0.008 | 3.41×10^-03^ | -0.006 | 0.012 | 0.608 |
| rs1340819 | 13 | 29145323 | 0.66 | A | C | 0.015 | 0.002 | 7.04×10^-10^ | 0.005 | 0.007 | 0.443 | 0.018 | 0.009 | 4.54×10^-02^ | -0.022 | 0.013 | 0.103 |
| rs1396514 | 17 | 68431026 | 0.51 | T | C | -0.036 | 0.002 | 1.54×10^-56^ | 0.003 | 0.006 | 0.601 | 0.002 | 0.007 | 0.794 | -0.002 | 0.011 | 0.887 |
| rs140201358 | 11 | 823586 | 0.99 | C | G | -0.098 | 0.012 | 3.49×10^-17^ | -0.001 | 0.032 | 0.987 | -0.054 | 0.039 | 0.168 | -0.004 | 0.063 | 0.946 |
| rs140664623 | 14 | 52321987 | 0.02 | T | G | -0.065 | 0.009 | 7.54×10^-13^ | 0.008 | 0.021 | 0.709 | 0.016 | 0.027 | 0.549 | 0.011 | 0.041 | 0.791 |
| rs144033177 | 20 | 571467 | 0.98 | A | C | -0.090 | 0.011 | 7.32×10^-16^ | 0.045 | 0.027 | 0.091 | 0.036 | 0.034 | 0.284 | 0.065 | 0.055 | 0.236 |
| rs145878042 | 12 | 48143315 | 0.99 | A | G | 0.105 | 0.013 | 1.74×10^-15^ | 0.025 | 0.030 | 0.401 | 0.020 | 0.037 | 0.588 | -0.013 | 0.058 | 0.818 |
| rs146385050 | 17 | 60637258 | 0.18 | A | C | 0.021 | 0.004 | 3.32×10^-09^ | 0.007 | 0.009 | 0.411 | 0.019 | 0.011 | 0.104 | -0.013 | 0.018 | 0.478 |
| rs1468602 | 3 | 43742734 | 0.98 | T | C | 0.048 | 0.008 | 1.15×10^-10^ | -0.037 | 0.021 | 0.082 | -0.031 | 0.027 | 0.246 | -0.106 | 0.040 | 7.47×10^-03^ |
| rs148358468 | 2 | 219590348 | 0.05 | A | G | 0.043 | 0.006 | 4.85×10^-12^ | 0.000 | 0.017 | 0.991 | -0.012 | 0.022 | 0.587 | 0.043 | 0.033 | 0.191 |
| rs1520325 | 4 | 125182690 | 0.23 | T | C | 0.017 | 0.003 | 2.29×10^-09^ | -0.015 | 0.007 | 3.84×10^-02^ | -0.021 | 0.009 | 1.69×10^-02^ | 0.001 | 0.013 | 0.966 |
| rs1522811 | 2 | 226992454 | 0.23 | A | C | -0.023 | 0.003 | 5.31×10^-16^ | 0.013 | 0.007 | 0.078 | 0.023 | 0.009 | 1.27×10^-02^ | 0.005 | 0.014 | 0.704 |
| rs1534696 | 7 | 26397239 | 0.57 | A | C | -0.040 | 0.002 | 8.10×10^-64^ | 0.008 | 0.006 | 0.174 | 0.003 | 0.008 | 0.682 | 0.014 | 0.012 | 0.231 |
| rs1535179 | 1 | 23639009 | 0.93 | A | G | -0.030 | 0.005 | 4.62×10^-10^ | -0.002 | 0.013 | 0.899 | -0.021 | 0.016 | 0.192 | 0.013 | 0.025 | 0.605 |
| rs1578916 | 7 | 150281975 | 0.24 | T | C | 0.018 | 0.003 | 7.40×10^-11^ | -0.013 | 0.007 | 0.066 | -0.014 | 0.009 | 0.118 | -0.029 | 0.013 | 2.86×10^-02^ |
| rs1635853 | 7 | 28189549 | 0.41 | T | G | 0.016 | 0.002 | 7.52×10^-12^ | -0.011 | 0.006 | 0.059 | -0.027 | 0.008 | 3.98×10^-04^ | 0.001 | 0.011 | 0.927 |
| rs16907277 | 9 | 93972658 | 0.89 | A | G | -0.027 | 0.004 | 2.82×10^-12^ | 0.018 | 0.010 | 0.071 | 0.028 | 0.013 | 3.02×10^-02^ | 0.017 | 0.019 | 0.378 |
| rs16935416 | 9 | 17235156 | 0.10 | A | G | -0.026 | 0.004 | 6.54×10^-10^ | -0.020 | 0.011 | 0.068 | -0.014 | 0.014 | 0.292 | -0.028 | 0.021 | 0.176 |
| rs16975388 | 16 | 85261963 | 0.78 | C | G | 0.019 | 0.003 | 6.03×10^-10^ | 0.003 | 0.007 | 0.690 | -0.006 | 0.009 | 0.542 | -0.009 | 0.014 | 0.505 |
| rs16978854 | 19 | 13139660 | 0.04 | A | G | -0.042 | 0.006 | 7.51×10^-11^ | -0.072 | 0.018 | 8.42×10^-05^ | -0.062 | 0.023 | 8.25×10^-03^ | -0.049 | 0.036 | 0.171 |
| rs17101456 | 10 | 122875040 | 0.88 | A | G | -0.037 | 0.004 | 2.99×10^-23^ | 0.012 | 0.009 | 0.191 | 0.011 | 0.011 | 0.325 | -0.003 | 0.017 | 0.869 |
| rs17184382 | 15 | 63792486 | 0.57 | A | C | 0.014 | 0.002 | 1.30×10^-09^ | -0.016 | 0.006 | 1.07×10^-02^ | -0.014 | 0.008 | 0.072 | -0.027 | 0.012 | 2.28×10^-02^ |
| rs17292540 | 9 | 119351429 | 0.22 | C | G | -0.018 | 0.003 | 2.14×10^-10^ | -0.039 | 0.007 | 3.77×10^-08^ | -0.051 | 0.009 | 4.67×10^-09^ | -0.046 | 0.013 | 6.90×10^-04^ |
| rs17326656 | 2 | 48962291 | 0.23 | T | G | 0.025 | 0.003 | 9.44×10^-19^ | 0.002 | 0.008 | 0.818 | 0.011 | 0.009 | 0.229 | -0.009 | 0.014 | 0.524 |
| rs17437657 | 7 | 27249617 | 0.09 | A | G | -0.042 | 0.004 | 4.57×10^-22^ | 0.000 | 0.011 | 1.000 | 0.007 | 0.014 | 0.617 | -0.001 | 0.022 | 0.978 |
| rs17451107 | 3 | 156797609 | 0.62 | T | C | 0.031 | 0.002 | 3.50×10^-39^ | 0.004 | 0.006 | 0.475 | 0.003 | 0.008 | 0.721 | 0.012 | 0.012 | 0.299 |
| rs17457629 | 5 | 38795199 | 0.72 | A | T | 0.019 | 0.003 | 8.63×10^-12^ | -0.002 | 0.007 | 0.833 | -0.002 | 0.009 | 0.855 | 0.002 | 0.014 | 0.863 |
| rs174829 | 3 | 37535577 | 0.35 | A | G | -0.015 | 0.003 | 3.00×10^-09^ | 0.008 | 0.007 | 0.203 | 0.020 | 0.008 | 1.29×10^-02^ | 0.028 | 0.012 | 2.47×10^-02^ |
| rs1800978 | 9 | 107665978 | 0.88 | C | G | 0.032 | 0.004 | 5.11×10^-20^ | 0.017 | 0.009 | 0.059 | 0.008 | 0.011 | 0.504 | 0.029 | 0.017 | 0.091 |
| rs1877569 | 3 | 99224544 | 0.19 | A | G | 0.019 | 0.003 | 1.52×10^-10^ | 0.002 | 0.008 | 0.821 | 0.003 | 0.010 | 0.774 | -0.004 | 0.015 | 0.780 |
| rs1902066 | 6 | 81346033 | 0.45 | T | C | -0.021 | 0.002 | 3.11×10^-19^ | 0.027 | 0.006 | 9.58×10^-06^ | 0.026 | 0.008 | 6.73×10^-04^ | 0.025 | 0.011 | 3.16×10^-02^ |
| rs1983189 | 5 | 54886681 | 0.32 | A | G | 0.024 | 0.003 | 9.65×10^-21^ | 0.002 | 0.007 | 0.777 | 0.001 | 0.008 | 0.917 | -0.010 | 0.012 | 0.405 |
| rs1997833 | 20 | 39690342 | 0.71 | T | C | -0.016 | 0.003 | 7.95×10^-11^ | 0.010 | 0.007 | 0.121 | 0.013 | 0.008 | 0.105 | 0.006 | 0.013 | 0.648 |
| rs2028386 | 15 | 74226708 | 0.48 | C | G | -0.015 | 0.002 | 8.89×10^-10^ | 0.015 | 0.006 | 1.14×10^-02^ | 0.015 | 0.007 | 3.93×10^-02^ | 0.014 | 0.011 | 0.208 |
| rs20478 | 13 | 44686064 | 0.10 | A | G | 0.026 | 0.004 | 1.07×10^-10^ | -0.007 | 0.010 | 0.498 | -0.011 | 0.013 | 0.387 | -0.051 | 0.020 | 9.26×10^-03^ |
| rs2072209 | 7 | 107592198 | 0.93 | A | G | 0.028 | 0.005 | 1.60×10^-09^ | 0.020 | 0.014 | 0.148 | 0.022 | 0.018 | 0.218 | -0.019 | 0.026 | 0.472 |
| rs2075665 | 9 | 101749943 | 0.62 | A | T | 0.016 | 0.003 | 2.13×10^-11^ | -0.008 | 0.007 | 0.212 | -0.001 | 0.008 | 0.922 | -0.011 | 0.012 | 0.387 |
| rs2153155 | 4 | 26357893 | 0.65 | T | C | -0.018 | 0.003 | 3.66×10^-13^ | 0.009 | 0.006 | 0.150 | 0.006 | 0.008 | 0.447 | -0.002 | 0.012 | 0.868 |
| rs2166365 | 5 | 3597830 | 0.78 | A | G | 0.018 | 0.003 | 1.94×10^-09^ | 0.004 | 0.008 | 0.618 | 0.010 | 0.010 | 0.344 | 0.035 | 0.015 | 2.00×10^-02^ |
| rs2207132 | 20 | 39142516 | 0.03 | A | G | 0.069 | 0.008 | 2.22×10^-20^ | 0.001 | 0.020 | 0.949 | -0.014 | 0.024 | 0.562 | 0.033 | 0.038 | 0.383 |
| rs2236519 | 20 | 45529571 | 0.37 | A | G | 0.035 | 0.003 | 7.00×10^-45^ | 0.003 | 0.007 | 0.683 | 0.007 | 0.008 | 0.382 | 0.001 | 0.012 | 0.938 |
| rs2270445 | 17 | 8219478 | 0.51 | A | G | -0.017 | 0.002 | 3.29×10^-12^ | 0.015 | 0.006 | 1.66×10^-02^ | 0.015 | 0.008 | 0.051 | 0.025 | 0.012 | 3.61×10^-02^ |
| rs2272790 | 22 | 35680095 | 0.69 | A | G | -0.015 | 0.003 | 2.82×10^-09^ | 0.008 | 0.007 | 0.280 | 0.001 | 0.009 | 0.922 | -0.002 | 0.013 | 0.907 |
| rs2277339 | 12 | 57146069 | 0.89 | T | G | -0.023 | 0.004 | 2.99×10^-09^ | 0.038 | 0.010 | 1.29×10^-04^ | 0.043 | 0.012 | 5.16×10^-04^ | 0.046 | 0.019 | 1.73×10^-02^ |
| rs2294239 | 22 | 29449477 | 0.57 | A | G | 0.026 | 0.002 | 4.21×10^-28^ | 0.007 | 0.006 | 0.248 | 0.010 | 0.008 | 0.186 | 0.001 | 0.011 | 0.947 |
| rs2300481 | 2 | 66782467 | 0.39 | T | C | 0.021 | 0.003 | 9.28×10^-18^ | 0.009 | 0.006 | 0.163 | 0.010 | 0.008 | 0.198 | 0.002 | 0.012 | 0.855 |
| rs2320125 | 17 | 62008318 | 0.35 | T | C | 0.015 | 0.003 | 1.36×10^-09^ | -0.010 | 0.006 | 0.097 | -0.007 | 0.008 | 0.406 | 0.008 | 0.012 | 0.524 |
| rs2369633 | 1 | 205181062 | 0.10 | T | C | -0.038 | 0.004 | 3.76×10^-20^ | -0.005 | 0.011 | 0.668 | 0.004 | 0.013 | 0.771 | -0.002 | 0.020 | 0.931 |
| rs2373078 | 2 | 218392555 | 0.10 | T | C | 0.033 | 0.004 | 8.82×10^-18^ | -0.019 | 0.010 | 4.92×10^-02^ | -0.015 | 0.012 | 0.217 | -0.046 | 0.019 | 1.27×10^-02^ |
| rs2459732 | 5 | 4027324 | 0.45 | T | C | -0.018 | 0.002 | 4.59×10^-14^ | 0.005 | 0.006 | 0.423 | 0.006 | 0.008 | 0.465 | 0.000 | 0.011 | 0.979 |
| rs2474714 | 10 | 33495983 | 0.57 | A | G | 0.015 | 0.002 | 1.55×10^-10^ | -0.011 | 0.006 | 0.059 | -0.011 | 0.008 | 0.135 | -0.026 | 0.011 | 2.21×10^-02^ |
| rs2503100 | 6 | 100613915 | 0.84 | A | G | -0.036 | 0.003 | 4.05×10^-31^ | 0.009 | 0.009 | 0.327 | 0.004 | 0.011 | 0.695 | -0.027 | 0.017 | 0.103 |
| rs2645294 | 1 | 119574587 | 0.56 | T | C | 0.035 | 0.002 | 2.86×10^-51^ | 0.006 | 0.006 | 0.319 | 0.005 | 0.008 | 0.505 | 0.016 | 0.012 | 0.159 |
| rs2682386 | 3 | 101068873 | 0.37 | T | C | 0.015 | 0.002 | 3.36×10^-10^ | 0.002 | 0.006 | 0.745 | 0.000 | 0.008 | 0.972 | -0.023 | 0.012 | 4.49×10^-02^ |
| rs2701013 | 7 | 37423214 | 0.67 | T | G | -0.016 | 0.003 | 5.85×10^-10^ | -0.011 | 0.006 | 0.078 | -0.012 | 0.008 | 0.124 | -0.008 | 0.012 | 0.508 |
| rs2791550 | 1 | 219655369 | 0.25 | T | G | -0.058 | 0.002 | 1.04×10^-124^ | -0.013 | 0.007 | 4.19×10^-02^ | -0.020 | 0.008 | 1.32×10^-02^ | 0.014 | 0.013 | 0.266 |
| rs2836162 | 21 | 39504356 | 0.60 | A | G | 0.020 | 0.002 | 1.95×10^-17^ | 0.003 | 0.006 | 0.575 | 0.009 | 0.008 | 0.249 | -0.020 | 0.012 | 0.084 |
| rs28378811 | 21 | 46779091 | 0.47 | T | G | 0.019 | 0.003 | 4.08×10^-13^ | -0.010 | 0.006 | 0.108 | -0.012 | 0.008 | 0.124 | 0.018 | 0.011 | 0.115 |
| rs28562046 | 9 | 139241595 | 0.24 | C | G | -0.019 | 0.003 | 6.84×10^-10^ | 0.005 | 0.008 | 0.509 | 0.001 | 0.010 | 0.915 | 0.027 | 0.015 | 0.066 |
| rs2925128 | 14 | 98362355 | 0.37 | T | C | -0.020 | 0.002 | 2.40×10^-16^ | 0.018 | 0.006 | 5.36×10^-03^ | 0.024 | 0.008 | 2.35×10^-03^ | 0.000 | 0.012 | 0.993 |
| rs2925979 | 16 | 81534790 | 0.30 | T | C | 0.045 | 0.003 | 2.29×10^-72^ | -0.007 | 0.007 | 0.270 | -0.002 | 0.008 | 0.827 | 0.001 | 0.012 | 0.963 |
| rs2993481 | 1 | 2973433 | 0.65 | A | T | -0.022 | 0.003 | 6.21×10^-13^ | -0.018 | 0.009 | 4.67×10^-02^ | -0.015 | 0.011 | 0.190 | -0.026 | 0.017 | 0.133 |
| rs299615 | 5 | 34042097 | 0.15 | A | G | -0.019 | 0.003 | 4.80×10^-09^ | -0.023 | 0.009 | 6.54×10^-03^ | -0.025 | 0.011 | 2.12×10^-02^ | -0.016 | 0.016 | 0.310 |
| rs332105 | 2 | 119444229 | 0.55 | A | G | -0.019 | 0.002 | 2.41×10^-15^ | 0.003 | 0.006 | 0.664 | -0.003 | 0.008 | 0.657 | 0.012 | 0.012 | 0.314 |
| rs34000 | 5 | 141973501 | 0.60 | T | C | 0.019 | 0.003 | 1.15×10^-14^ | 0.003 | 0.007 | 0.638 | 0.004 | 0.009 | 0.666 | 0.014 | 0.013 | 0.294 |
| rs34311866 | 4 | 951947 | 0.82 | T | C | -0.030 | 0.004 | 8.74×10^-18^ | 0.010 | 0.008 | 0.243 | 0.025 | 0.010 | 1.52×10^-02^ | 0.004 | 0.015 | 0.806 |
| rs34781 | 5 | 102454439 | 0.30 | T | C | -0.018 | 0.003 | 4.25×10^-12^ | -0.009 | 0.007 | 0.172 | -0.001 | 0.008 | 0.880 | -0.018 | 0.012 | 0.144 |
| rs351407 | 1 | 212437442 | 0.37 | T | C | -0.016 | 0.002 | 8.35×10^-11^ | -0.003 | 0.006 | 0.620 | 0.004 | 0.008 | 0.599 | -0.008 | 0.012 | 0.480 |
| rs35632432 | 19 | 4499850 | 0.31 | A | G | -0.018 | 0.003 | 9.92×10^-10^ | 0.008 | 0.007 | 0.270 | 0.006 | 0.009 | 0.492 | 0.012 | 0.013 | 0.361 |
| rs3741378 | 11 | 65408937 | 0.14 | T | C | -0.029 | 0.003 | 8.76×10^-19^ | 0.006 | 0.009 | 0.516 | 0.011 | 0.011 | 0.336 | 0.005 | 0.017 | 0.759 |
| rs3749748 | 5 | 127350549 | 0.23 | T | C | -0.018 | 0.003 | 2.39×10^-10^ | 0.027 | 0.007 | 1.20×10^-04^ | 0.029 | 0.009 | 9.72×10^-04^ | 0.035 | 0.013 | 8.15×10^-03^ |
| rs3773240 | 3 | 78783084 | 0.23 | T | C | 0.017 | 0.003 | 6.39×10^-10^ | -0.006 | 0.007 | 0.448 | -0.006 | 0.009 | 0.510 | -0.005 | 0.014 | 0.736 |
| rs3792751 | 5 | 32773314 | 0.36 | T | C | 0.018 | 0.002 | 5.21×10^-14^ | 0.005 | 0.007 | 0.445 | 0.006 | 0.008 | 0.450 | 0.008 | 0.012 | 0.509 |
| rs3803042 | 12 | 54387947 | 0.43 | A | G | 0.030 | 0.002 | 5.36×10^-36^ | -0.014 | 0.006 | 2.50×10^-02^ | -0.016 | 0.008 | 0.052 | 0.001 | 0.012 | 0.909 |
| rs3843375 | 3 | 196813908 | 0.41 | A | G | 0.016 | 0.003 | 1.57×10^-09^ | 0.001 | 0.006 | 0.919 | 0.005 | 0.008 | 0.510 | -0.007 | 0.012 | 0.575 |
| rs39312 | 7 | 116954785 | 0.62 | A | C | -0.017 | 0.002 | 5.90×10^-13^ | 0.001 | 0.006 | 0.923 | -0.009 | 0.008 | 0.223 | 0.003 | 0.012 | 0.819 |
| rs3936510 | 5 | 55860866 | 0.20 | T | G | 0.049 | 0.003 | 4.28×10^-64^ | -0.020 | 0.008 | 1.18×10^-02^ | -0.030 | 0.010 | 2.25×10^-03^ | -0.014 | 0.015 | 0.338 |
| rs4081724 | 19 | 33824946 | 0.14 | A | G | -0.040 | 0.003 | 4.40×10^-32^ | -0.008 | 0.009 | 0.359 | -0.014 | 0.011 | 0.197 | -0.009 | 0.016 | 0.586 |
| rs4130827 | 12 | 66430452 | 0.71 | T | G | -0.024 | 0.003 | 6.69×10^-19^ | 0.003 | 0.007 | 0.712 | 0.000 | 0.009 | 0.976 | 0.013 | 0.014 | 0.363 |
| rs4243085 | 15 | 79057723 | 0.76 | C | G | -0.020 | 0.003 | 3.60×10^-10^ | 0.011 | 0.007 | 0.122 | 0.012 | 0.009 | 0.171 | 0.014 | 0.013 | 0.302 |
| rs4246243 | 12 | 27518517 | 0.49 | A | C | -0.016 | 0.002 | 2.29×10^-11^ | 0.007 | 0.006 | 0.255 | 0.015 | 0.008 | 4.81×10^-02^ | -0.013 | 0.012 | 0.275 |
| rs4285804 | 10 | 104386309 | 0.55 | A | T | 0.019 | 0.002 | 1.07×10^-16^ | 0.009 | 0.006 | 0.140 | 0.011 | 0.007 | 0.153 | -0.008 | 0.011 | 0.504 |
| rs4371408 | 20 | 51024233 | 0.63 | A | G | 0.016 | 0.003 | 1.94×10^-11^ | 0.012 | 0.007 | 0.075 | 0.016 | 0.008 | 4.98×10^-02^ | 0.008 | 0.012 | 0.530 |
| rs4372913 | 2 | 114517748 | 0.79 | A | G | -0.019 | 0.003 | 3.88×10^-11^ | -0.009 | 0.008 | 0.246 | -0.008 | 0.009 | 0.410 | -0.004 | 0.014 | 0.766 |
| rs4474021 | 8 | 12632903 | 0.31 | T | G | 0.016 | 0.003 | 1.53×10^-10^ | -0.006 | 0.007 | 0.404 | -0.006 | 0.009 | 0.532 | 0.001 | 0.014 | 0.950 |
| rs4531856 | 19 | 18388383 | 0.64 | T | C | -0.027 | 0.003 | 1.66×10^-27^ | -0.007 | 0.006 | 0.283 | -0.004 | 0.008 | 0.657 | 0.000 | 0.012 | 0.997 |
| rs4671193 | 2 | 67846288 | 0.36 | T | C | -0.021 | 0.003 | 3.44×10^-17^ | -0.015 | 0.006 | 1.67×10^-02^ | -0.008 | 0.008 | 0.296 | -0.017 | 0.012 | 0.147 |
| rs4686696 | 3 | 185516520 | 0.31 | A | G | 0.019 | 0.002 | 1.17×10^-14^ | -0.015 | 0.006 | 2.13×10^-02^ | -0.022 | 0.008 | 6.76×10^-03^ | -0.013 | 0.012 | 0.294 |
| rs4871958 | 8 | 25762216 | 0.51 | A | G | 0.016 | 0.002 | 5.90×10^-12^ | 0.001 | 0.006 | 0.888 | -0.001 | 0.008 | 0.894 | 0.001 | 0.012 | 0.933 |
| rs4894803 | 3 | 171800256 | 0.60 | A | G | 0.019 | 0.002 | 3.14×10^-14^ | 0.011 | 0.007 | 0.079 | 0.009 | 0.008 | 0.275 | 0.013 | 0.012 | 0.300 |
| rs4934618 | 10 | 34167569 | 0.42 | A | G | 0.018 | 0.002 | 3.66×10^-13^ | -0.015 | 0.006 | 1.42×10^-02^ | -0.005 | 0.008 | 0.557 | -0.028 | 0.012 | 2.02×10^-02^ |
| rs4964656 | 12 | 108594069 | 0.30 | C | G | -0.018 | 0.003 | 2.98×10^-12^ | 0.000 | 0.008 | 0.947 | -0.002 | 0.010 | 0.854 | 0.009 | 0.014 | 0.539 |
| rs501470 | 6 | 160770918 | 0.50 | T | G | 0.025 | 0.002 | 4.22×10^-27^ | -0.005 | 0.006 | 0.384 | -0.011 | 0.007 | 0.133 | -0.003 | 0.011 | 0.773 |
| rs5117 | 19 | 45418790 | 0.77 | T | C | 0.027 | 0.003 | 1.35×10^-17^ | 0.013 | 0.008 | 0.085 | 0.017 | 0.009 | 0.074 | 0.006 | 0.014 | 0.686 |
| rs552125 | 13 | 110938813 | 0.12 | T | C | -0.022 | 0.004 | 2.05×10^-10^ | -0.015 | 0.009 | 0.095 | -0.004 | 0.011 | 0.683 | -0.036 | 0.017 | 3.18×10^-02^ |
| rs55747707 | 7 | 73037366 | 0.20 | A | G | -0.036 | 0.003 | 2.19×10^-26^ | 0.026 | 0.008 | 1.52×10^-03^ | 0.031 | 0.011 | 3.49×10^-03^ | 0.017 | 0.016 | 0.284 |
| rs55818584 | 19 | 10332547 | 0.23 | A | T | -0.019 | 0.003 | 2.00×10^-09^ | 0.018 | 0.008 | 2.05×10^-02^ | 0.011 | 0.010 | 0.233 | 0.014 | 0.014 | 0.347 |
| rs55920843 | 2 | 158412701 | 0.99 | T | G | 0.117 | 0.013 | 1.89×10^-20^ | -0.046 | 0.032 | 0.159 | -0.049 | 0.041 | 0.227 | -0.154 | 0.061 | 1.23×10^-02^ |
| rs56271783 | 11 | 64004723 | 0.04 | C | G | 0.089 | 0.007 | 1.11×10^-41^ | -0.021 | 0.015 | 0.160 | 0.005 | 0.018 | 0.783 | -0.038 | 0.029 | 0.195 |
| rs5997898 | 22 | 31572963 | 0.42 | A | C | -0.014 | 0.002 | 4.39×10^-09^ | 0.001 | 0.006 | 0.841 | 0.004 | 0.008 | 0.575 | -0.023 | 0.012 | 4.78×10^-02^ |
| rs605066 | 6 | 139829666 | 0.58 | T | C | -0.035 | 0.002 | 3.52×10^-48^ | 0.000 | 0.006 | 0.970 | -0.005 | 0.008 | 0.496 | 0.015 | 0.012 | 0.192 |
| rs6062344 | 20 | 62696024 | 0.50 | T | C | -0.025 | 0.003 | 8.18×10^-25^ | 0.027 | 0.006 | 1.43×10^-05^ | 0.029 | 0.008 | 2.38×10^-04^ | 0.013 | 0.012 | 0.260 |
| rs62070804 | 17 | 27889643 | 0.02 | T | C | 0.079 | 0.010 | 9.66×10^-15^ | 0.027 | 0.032 | 0.403 | 0.007 | 0.040 | 0.868 | -0.006 | 0.063 | 0.919 |
| rs62271373 | 3 | 150066540 | 0.06 | A | T | 0.069 | 0.006 | 2.22×10^-32^ | -0.011 | 0.015 | 0.463 | -0.013 | 0.020 | 0.509 | 0.000 | 0.029 | 0.992 |
| rs62319568 | 4 | 120362961 | 0.86 | A | G | -0.027 | 0.004 | 9.66×10^-13^ | -0.003 | 0.009 | 0.759 | 0.005 | 0.011 | 0.680 | -0.002 | 0.017 | 0.888 |
| rs6426912 | 1 | 165321568 | 0.11 | T | C | 0.025 | 0.004 | 9.31×10^-11^ | -0.010 | 0.011 | 0.346 | -0.006 | 0.013 | 0.655 | 0.018 | 0.020 | 0.364 |
| rs6498114 | 16 | 10964118 | 0.77 | T | G | -0.017 | 0.003 | 1.47×10^-09^ | -0.001 | 0.008 | 0.934 | -0.007 | 0.010 | 0.465 | 0.017 | 0.014 | 0.242 |
| rs6688233 | 1 | 9335745 | 0.24 | T | C | 0.028 | 0.003 | 1.07×10^-23^ | 0.005 | 0.008 | 0.536 | 0.015 | 0.010 | 0.118 | -0.001 | 0.015 | 0.960 |
| rs6691427 | 1 | 203510048 | 0.49 | C | G | -0.022 | 0.003 | 4.80×10^-17^ | 0.009 | 0.007 | 0.197 | 0.014 | 0.009 | 0.107 | 0.004 | 0.014 | 0.790 |
| rs6719428 | 2 | 66238005 | 0.70 | T | C | -0.025 | 0.003 | 2.58×10^-22^ | 0.004 | 0.007 | 0.576 | 0.009 | 0.008 | 0.283 | 0.005 | 0.013 | 0.679 |
| rs6795831 | 3 | 129341403 | 0.82 | A | C | 0.052 | 0.003 | 4.65×10^-67^ | -0.017 | 0.008 | 2.83×10^-02^ | -0.026 | 0.010 | 7.13×10^-03^ | -0.016 | 0.015 | 0.282 |
| rs6878122 | 5 | 76427311 | 0.70 | A | G | -0.015 | 0.003 | 4.82×10^-09^ | -0.001 | 0.007 | 0.884 | 0.008 | 0.008 | 0.309 | -0.013 | 0.013 | 0.300 |
| rs6905288 | 6 | 43758873 | 0.57 | A | G | 0.061 | 0.002 | 3.87×10^-144^ | -0.020 | 0.006 | 1.15×10^-03^ | -0.024 | 0.008 | 2.10×10^-03^ | -0.004 | 0.012 | 0.709 |
| rs6932767 | 6 | 14595873 | 0.79 | T | G | 0.018 | 0.003 | 1.58×10^-09^ | -0.004 | 0.008 | 0.603 | -0.004 | 0.009 | 0.686 | -0.001 | 0.014 | 0.951 |
| rs6940715 | 6 | 133601845 | 0.89 | A | C | 0.028 | 0.004 | 1.44×10^-12^ | 0.005 | 0.010 | 0.628 | -0.008 | 0.013 | 0.533 | -0.001 | 0.019 | 0.952 |
| rs695115 | 18 | 2850548 | 0.30 | T | C | 0.018 | 0.003 | 6.78×10^-10^ | 0.011 | 0.007 | 0.127 | 0.016 | 0.009 | 0.075 | 0.012 | 0.014 | 0.387 |
| rs7014168 | 8 | 10641965 | 0.22 | A | G | 0.017 | 0.003 | 1.42×10^-09^ | -0.015 | 0.007 | 4.22×10^-02^ | -0.013 | 0.009 | 0.146 | -0.005 | 0.014 | 0.697 |
| rs7020604 | 9 | 112583554 | 0.66 | A | G | 0.019 | 0.003 | 2.32×10^-11^ | -0.010 | 0.007 | 0.128 | -0.009 | 0.008 | 0.279 | 0.000 | 0.012 | 0.975 |
| rs7086377 | 10 | 3596526 | 0.39 | T | G | 0.018 | 0.002 | 3.60×10^-13^ | -0.008 | 0.006 | 0.171 | -0.008 | 0.008 | 0.311 | -0.014 | 0.012 | 0.242 |
| rs7102 | 16 | 11642242 | 0.65 | T | C | -0.019 | 0.002 | 1.27×10^-14^ | -0.004 | 0.006 | 0.533 | 0.002 | 0.008 | 0.769 | -0.008 | 0.012 | 0.529 |
| rs711869 | 2 | 13073967 | 0.56 | A | G | -0.017 | 0.002 | 4.83×10^-13^ | 0.003 | 0.006 | 0.631 | 0.000 | 0.008 | 0.983 | -0.007 | 0.012 | 0.535 |
| rs7119797 | 11 | 62166235 | 0.49 | T | C | -0.015 | 0.002 | 1.13×10^-10^ | 0.010 | 0.006 | 0.113 | 0.013 | 0.008 | 0.081 | 0.016 | 0.011 | 0.150 |
| rs71363019 | 18 | 47014122 | 0.03 | T | C | 0.046 | 0.008 | 1.25×10^-09^ | 0.009 | 0.017 | 0.598 | -0.004 | 0.022 | 0.858 | 0.007 | 0.034 | 0.831 |
| rs71439172 | 2 | 25584162 | 0.14 | A | G | -0.029 | 0.004 | 1.99×10^-14^ | -0.022 | 0.009 | 1.38×10^-02^ | -0.012 | 0.011 | 0.287 | -0.020 | 0.017 | 0.236 |
| rs7154498 | 14 | 58762532 | 0.28 | C | G | 0.023 | 0.003 | 2.56×10^-15^ | 0.008 | 0.007 | 0.221 | 0.003 | 0.008 | 0.680 | 0.012 | 0.013 | 0.358 |
| rs7221005 | 17 | 67590431 | 0.33 | T | C | 0.019 | 0.003 | 5.37×10^-13^ | 0.000 | 0.007 | 0.976 | 0.007 | 0.008 | 0.402 | 0.001 | 0.012 | 0.963 |
| rs727428 | 17 | 7537792 | 0.46 | T | C | 0.019 | 0.002 | 8.73×10^-15^ | 0.002 | 0.006 | 0.760 | 0.001 | 0.007 | 0.884 | 0.001 | 0.011 | 0.907 |
| rs7279347 | 21 | 47537882 | 0.51 | A | G | -0.015 | 0.002 | 2.49×10^-10^ | 0.011 | 0.006 | 0.063 | 0.020 | 0.007 | 6.08×10^-03^ | 0.003 | 0.011 | 0.784 |
| rs72801474 | 5 | 132444128 | 0.09 | A | G | -0.031 | 0.005 | 2.12×10^-11^ | -0.036 | 0.012 | 3.45×10^-03^ | -0.049 | 0.016 | 1.54×10^-03^ | -0.012 | 0.024 | 0.619 |
| rs72837816 | 2 | 111916868 | 0.90 | A | C | 0.040 | 0.005 | 9.51×10^-19^ | 0.017 | 0.010 | 0.075 | 0.011 | 0.012 | 0.338 | 0.015 | 0.018 | 0.414 |
| rs72959041 | 6 | 127454893 | 0.05 | A | G | 0.217 | 0.006 | 1.00×10^-200^ | -0.059 | 0.015 | 9.44×10^-05^ | -0.072 | 0.019 | 1.12×10^-04^ | -0.008 | 0.029 | 0.785 |
| rs7395513 | 11 | 69262756 | 0.44 | A | G | -0.027 | 0.003 | 1.10×10^-23^ | -0.037 | 0.006 | 1.16×10^-09^ | -0.040 | 0.007 | 5.75×10^-08^ | 0.003 | 0.011 | 0.791 |
| rs7451021 | 6 | 130381246 | 0.32 | T | C | -0.018 | 0.003 | 3.37×10^-12^ | -0.044 | 0.007 | 8.71×10^-12^ | -0.042 | 0.008 | 1.40×10^-07^ | -0.060 | 0.012 | 1.15×10^-06^ |
| rs747249 | 11 | 130271647 | 0.36 | A | G | 0.016 | 0.003 | 1.02×10^-10^ | -0.004 | 0.007 | 0.602 | -0.011 | 0.009 | 0.193 | 0.016 | 0.013 | 0.218 |
| rs74809942 | 2 | 191749705 | 0.92 | T | C | 0.029 | 0.005 | 2.31×10^-09^ | -0.003 | 0.011 | 0.821 | -0.021 | 0.014 | 0.144 | 0.008 | 0.022 | 0.699 |
| rs7492628 | 14 | 91547136 | 0.68 | C | G | -0.029 | 0.003 | 1.45×10^-28^ | 0.005 | 0.007 | 0.447 | 0.001 | 0.008 | 0.930 | 0.031 | 0.012 | 1.18×10^-02^ |
| rs74963256 | 20 | 51588847 | 0.90 | T | C | -0.031 | 0.005 | 6.73×10^-12^ | 0.002 | 0.011 | 0.861 | 0.002 | 0.014 | 0.896 | -0.008 | 0.020 | 0.688 |
| rs757608 | 17 | 59497277 | 0.32 | A | G | 0.019 | 0.002 | 1.98×10^-14^ | 0.011 | 0.006 | 0.093 | 0.010 | 0.008 | 0.186 | 0.003 | 0.012 | 0.779 |
| rs7585974 | 2 | 172377212 | 0.18 | C | G | 0.028 | 0.003 | 7.28×10^-16^ | 0.023 | 0.008 | 3.52×10^-03^ | 0.025 | 0.010 | 8.86×10^-03^ | 0.008 | 0.015 | 0.601 |
| rs76306613 | 7 | 106235348 | 0.14 | T | G | 0.023 | 0.004 | 1.95×10^-09^ | -0.015 | 0.009 | 0.099 | -0.019 | 0.011 | 0.097 | -0.007 | 0.017 | 0.700 |
| rs7679205 | 4 | 5003150 | 0.12 | A | G | 0.024 | 0.004 | 4.25×10^-09^ | -0.006 | 0.010 | 0.526 | -0.003 | 0.013 | 0.793 | -0.027 | 0.019 | 0.170 |
| rs7680787 | 4 | 122624065 | 0.65 | T | C | 0.016 | 0.002 | 2.39×10^-11^ | 0.011 | 0.006 | 0.103 | 0.015 | 0.008 | 0.063 | -0.001 | 0.012 | 0.915 |
| rs7783857 | 7 | 130439058 | 0.73 | C | G | -0.032 | 0.003 | 4.10×10^-35^ | 0.022 | 0.007 | 1.80×10^-03^ | 0.026 | 0.009 | 2.76×10^-03^ | 0.008 | 0.013 | 0.552 |
| rs7795371 | 7 | 77362504 | 0.56 | A | G | 0.022 | 0.002 | 1.10×10^-19^ | -0.004 | 0.006 | 0.511 | -0.001 | 0.008 | 0.899 | -0.005 | 0.012 | 0.700 |
| rs7813520 | 8 | 26263260 | 0.70 | T | C | 0.015 | 0.003 | 1.32×10^-09^ | 0.002 | 0.007 | 0.795 | 0.020 | 0.009 | 2.16×10^-02^ | -0.019 | 0.013 | 0.145 |
| rs7834111 | 8 | 135698274 | 0.58 | T | C | 0.017 | 0.002 | 1.00×10^-12^ | -0.001 | 0.006 | 0.845 | -0.004 | 0.008 | 0.588 | 0.005 | 0.012 | 0.657 |
| rs7858732 | 9 | 131563540 | 0.75 | A | G | -0.016 | 0.003 | 4.22×10^-09^ | -0.007 | 0.007 | 0.359 | -0.011 | 0.009 | 0.219 | 0.016 | 0.013 | 0.222 |
| rs7903146 | 10 | 114758349 | 0.28 | T | C | 0.022 | 0.003 | 1.54×10^-17^ | 0.038 | 0.007 | 1.67×10^-08^ | 0.031 | 0.008 | 1.90×10^-04^ | 0.039 | 0.013 | 2.00×10^-03^ |
| rs7932891 | 11 | 10921512 | 0.30 | A | G | 0.017 | 0.003 | 6.61×10^-11^ | -0.016 | 0.007 | 3.40×10^-02^ | -0.020 | 0.009 | 3.24×10^-02^ | -0.007 | 0.014 | 0.611 |
| rs7954697 | 12 | 125335253 | 0.61 | A | C | 0.016 | 0.002 | 4.59×10^-12^ | 0.004 | 0.006 | 0.494 | 0.002 | 0.008 | 0.843 | 0.012 | 0.012 | 0.289 |
| rs797486 | 13 | 51221618 | 0.89 | A | C | 0.038 | 0.004 | 4.32×10^-27^ | -0.010 | 0.009 | 0.249 | -0.011 | 0.011 | 0.317 | 0.002 | 0.017 | 0.918 |
| rs7993238 | 13 | 22473593 | 0.65 | A | C | -0.015 | 0.003 | 3.89×10^-09^ | -0.004 | 0.007 | 0.586 | -0.001 | 0.008 | 0.868 | -0.017 | 0.013 | 0.173 |
| rs8054299 | 16 | 53498655 | 0.69 | C | G | 0.021 | 0.003 | 1.87×10^-15^ | -0.008 | 0.006 | 0.210 | -0.013 | 0.008 | 0.102 | 0.018 | 0.012 | 0.128 |
| rs805768 | 20 | 5666891 | 0.24 | T | C | 0.018 | 0.003 | 4.65×10^-09^ | -0.013 | 0.008 | 0.110 | 0.040 | 0.074 | 0.585 | 0.159 | 0.117 | 0.174 |
| rs8073599 | 17 | 65367094 | 0.37 | T | C | 0.014 | 0.002 | 4.28×10^-09^ | -0.005 | 0.007 | 0.489 | 0.002 | 0.008 | 0.792 | -0.005 | 0.013 | 0.685 |
| rs8074638 | 17 | 53731579 | 0.19 | A | G | 0.021 | 0.003 | 1.56×10^-11^ | 0.024 | 0.008 | 3.84×10^-03^ | 0.024 | 0.011 | 2.53×10^-02^ | 0.027 | 0.016 | 0.094 |
| rs8078513 | 17 | 17431208 | 0.05 | A | C | 0.042 | 0.006 | 1.28×10^-14^ | -0.011 | 0.013 | 0.389 | -0.034 | 0.016 | 4.03×10^-02^ | -0.039 | 0.025 | 0.120 |
| rs8079062 | 17 | 74255029 | 0.92 | A | G | -0.039 | 0.004 | 2.99×10^-19^ | 0.007 | 0.012 | 0.572 | 0.002 | 0.015 | 0.914 | -0.001 | 0.022 | 0.964 |
| rs8086644 | 18 | 60788559 | 0.17 | A | G | 0.019 | 0.003 | 4.97×10^-09^ | -0.006 | 0.008 | 0.458 | -0.013 | 0.010 | 0.212 | -0.022 | 0.016 | 0.162 |
| rs8094261 | 18 | 20746728 | 0.79 | C | G | 0.019 | 0.003 | 1.39×10^-10^ | -0.017 | 0.008 | 2.36×10^-02^ | -0.020 | 0.010 | 3.77×10^-02^ | -0.013 | 0.015 | 0.392 |
| rs8103017 | 19 | 55999142 | 0.70 | C | G | -0.023 | 0.003 | 1.48×10^-14^ | -0.019 | 0.007 | 8.94×10^-03^ | -0.021 | 0.009 | 1.87×10^-02^ | -0.022 | 0.014 | 0.102 |
| rs821107 | 8 | 89500663 | 0.29 | A | C | 0.021 | 0.003 | 1.19×10^-12^ | -0.001 | 0.007 | 0.922 | -0.003 | 0.008 | 0.751 | 0.015 | 0.013 | 0.217 |
| rs881299 | 8 | 38332249 | 0.59 | T | C | -0.016 | 0.003 | 1.89×10^-09^ | 0.008 | 0.006 | 0.211 | 0.014 | 0.008 | 0.072 | -0.001 | 0.012 | 0.953 |
| rs890498 | 15 | 42111298 | 0.65 | C | G | -0.021 | 0.003 | 5.27×10^-16^ | -0.007 | 0.006 | 0.284 | -0.009 | 0.008 | 0.235 | 0.001 | 0.012 | 0.941 |
| rs905938 | 1 | 154991389 | 0.72 | T | C | 0.032 | 0.003 | 1.41×10^-34^ | 0.020 | 0.007 | 4.31×10^-03^ | 0.021 | 0.009 | 1.55×10^-02^ | 0.008 | 0.013 | 0.526 |
| rs910382 | 20 | 51699189 | 0.49 | A | G | -0.026 | 0.002 | 8.90×10^-27^ | 0.007 | 0.006 | 0.281 | 0.005 | 0.008 | 0.518 | 0.018 | 0.012 | 0.136 |
| rs917191 | 7 | 80570871 | 0.57 | C | G | 0.022 | 0.002 | 1.04×10^-19^ | -0.005 | 0.007 | 0.463 | 0.004 | 0.008 | 0.639 | -0.015 | 0.013 | 0.223 |
| rs931619 | 5 | 178514729 | 0.36 | A | G | 0.015 | 0.002 | 1.96×10^-09^ | 0.004 | 0.006 | 0.497 | 0.004 | 0.008 | 0.647 | 0.013 | 0.012 | 0.302 |
| rs9415106 | 10 | 77295957 | 0.71 | A | G | 0.017 | 0.003 | 6.71×10^-11^ | 0.011 | 0.007 | 0.114 | 0.020 | 0.009 | 1.79×10^-02^ | -0.006 | 0.013 | 0.628 |
| rs9436299 | 1 | 65892888 | 0.66 | A | C | 0.017 | 0.003 | 2.93×10^-11^ | -0.006 | 0.007 | 0.387 | 0.005 | 0.008 | 0.517 | -0.007 | 0.012 | 0.568 |
| rs952632 | 12 | 124469738 | 0.66 | A | G | 0.056 | 0.003 | 2.80×10^-111^ | -0.007 | 0.007 | 0.315 | -0.004 | 0.008 | 0.630 | 0.008 | 0.012 | 0.500 |
| rs954244 | 2 | 121309231 | 0.76 | C | G | -0.021 | 0.003 | 1.97×10^-13^ | 0.007 | 0.007 | 0.316 | 0.005 | 0.009 | 0.610 | 0.008 | 0.014 | 0.546 |
| rs9644033 | 8 | 23610639 | 0.75 | A | T | 0.038 | 0.003 | 3.00×10^-42^ | -0.009 | 0.007 | 0.175 | -0.007 | 0.009 | 0.409 | -0.013 | 0.013 | 0.341 |
| rs9674436 | 16 | 1342841 | 0.47 | A | G | 0.017 | 0.003 | 1.71×10^-10^ | 0.009 | 0.006 | 0.158 | 0.008 | 0.008 | 0.303 | -0.003 | 0.012 | 0.820 |
| rs9678859 | 2 | 100288478 | 0.18 | A | G | 0.023 | 0.003 | 1.62×10^-13^ | 0.000 | 0.008 | 0.965 | 0.006 | 0.010 | 0.546 | -0.009 | 0.015 | 0.546 |
| rs979012 | 20 | 6623374 | 0.36 | T | C | 0.020 | 0.002 | 1.90×10^-17^ | 0.011 | 0.006 | 0.092 | 0.013 | 0.008 | 0.093 | 0.025 | 0.012 | 4.11×10^-02^ |
| rs9848655 | 3 | 138108352 | 0.16 | A | G | 0.029 | 0.003 | 4.30×10^-20^ | 0.001 | 0.008 | 0.931 | -0.001 | 0.010 | 0.954 | -0.008 | 0.016 | 0.607 |
| rs9871235 | 3 | 107494345 | 0.61 | A | G | 0.014 | 0.002 | 9.59×10^-10^ | 0.008 | 0.006 | 0.188 | 0.011 | 0.008 | 0.157 | 0.007 | 0.012 | 0.532 |
| rs988799 | 4 | 106126413 | 0.79 | C | G | -0.023 | 0.003 | 3.14×10^-15^ | -0.041 | 0.007 | 7.01×10^-09^ | -0.052 | 0.009 | 2.23×10^-09^ | -0.011 | 0.013 | 0.407 |
| rs9892728 | 17 | 40913366 | 0.45 | T | C | 0.020 | 0.003 | 1.24×10^-13^ | -0.002 | 0.006 | 0.722 | -0.003 | 0.007 | 0.656 | 0.007 | 0.011 | 0.530 |
| rs9991328 | 4 | 89713121 | 0.47 | T | C | 0.041 | 0.002 | 5.34×10^-70^ | 0.008 | 0.006 | 0.160 | 0.000 | 0.007 | 0.953 | 0.015 | 0.011 | 0.194 |
| **childhood BMI** |  |  |  |  |  |  |  |  |  |  |  |  |  |  |  |  |  |
| rs1094647 | 1 | 205655378 | 0.55 | G | A | 0.037 | 0.006 | 3.49×10^-10^ | 0.008 | 0.006 | 0.176 | 0.009 | 0.007 | 0.235 | 0.006 | 0.011 | 0.624 |
| rs11030391 | 11 | 28644626 | 0.63 | A | G | 0.035 | 0.007 | 2.87×10^-07^ | -0.005 | 0.006 | 0.469 | -0.003 | 0.008 | 0.699 | 0.006 | 0.012 | 0.605 |
| rs11215427 | 11 | 115093438 | 0.73 | G | C | 0.039 | 0.007 | 1.26×10^-08^ | -0.007 | 0.007 | 0.346 | 0.000 | 0.009 | 0.995 | 0.005 | 0.013 | 0.705 |
| rs114285994 | 16 | 19935763 | 0.88 | G | A | 0.060 | 0.010 | 9.87×10^-10^ | -0.014 | 0.009 | 0.126 | -0.003 | 0.011 | 0.813 | -0.035 | 0.017 | 3.87×10^-02^ |
| rs114670539 | 2 | 207064335 | 0.05 | T | C | 0.088 | 0.015 | 2.22×10^-09^ | -0.027 | 0.014 | 4.90×10^-02^ | -0.004 | 0.017 | 0.817 | 0.017 | 0.026 | 0.515 |
| rs116664060 | 6 | 31592524 | 0.19 | C | G | 0.052 | 0.009 | 3.78×10^-09^ | -0.002 | 0.008 | 0.797 | -0.007 | 0.010 | 0.497 | 0.019 | 0.015 | 0.216 |
| rs11676272 | 2 | 25141538 | 0.47 | G | A | 0.072 | 0.006 | 1.78×10^-33^ | -0.034 | 0.006 | 5.83×10^-08^ | -0.033 | 0.008 | 2.76×10^-05^ | -0.054 | 0.012 | 6.82×10^-06^ |
| rs12042908 | 1 | 74997762 | 0.45 | A | G | 0.060 | 0.006 | 7.62×10^-24^ | -0.006 | 0.006 | 0.285 | -0.012 | 0.007 | 0.105 | -0.014 | 0.011 | 0.228 |
| rs12641981 | 4 | 45179883 | 0.43 | T | C | 0.047 | 0.007 | 9.45×10^-12^ | -0.014 | 0.006 | 1.73×10^-02^ | -0.018 | 0.007 | 1.31×10^-02^ | -0.022 | 0.011 | 0.053 |
| rs13107325 | 4 | 103188709 | 0.07 | T | C | 0.099 | 0.014 | 7.67×10^-13^ | 0.019 | 0.012 | 0.119 | 0.020 | 0.015 | 0.178 | -0.006 | 0.023 | 0.776 |
| rs144376234 | 1 | 110114504 | 0.04 | T | C | 0.112 | 0.018 | 2.45×10^-10^ | -0.069 | 0.018 | 7.87×10^-05^ | -0.042 | 0.022 | 0.051 | -0.106 | 0.034 | 1.82×10^-03^ |
| rs17817449 | 16 | 53813367 | 0.40 | G | T | 0.073 | 0.007 | 9.18×10^-26^ | -0.057 | 0.006 | 6.22×10^-21^ | -0.056 | 0.008 | 5.59×10^-14^ | -0.074 | 0.012 | 1.80×10^-10^ |
| rs2076308 | 6 | 50791640 | 0.19 | C | G | 0.058 | 0.008 | 2.08×10^-13^ | 0.005 | 0.008 | 0.502 | 0.016 | 0.010 | 0.095 | -0.042 | 0.015 | 4.40×10^-03^ |
| rs4477562 | 13 | 54104968 | 0.13 | T | C | 0.069 | 0.009 | 8.83×10^-15^ | 0.005 | 0.009 | 0.618 | 0.009 | 0.012 | 0.423 | 0.036 | 0.018 | 4.31×10^-02^ |
| rs543874 | 1 | 177889480 | 0.20 | G | A | 0.077 | 0.008 | 3.14×10^-22^ | -0.034 | 0.008 | 1.00×10^-05^ | -0.025 | 0.009 | 7.28×10^-03^ | -0.014 | 0.014 | 0.327 |
| rs56133711 | 11 | 27723334 | 0.25 | A | G | 0.056 | 0.007 | 6.22×10^-16^ | -0.005 | 0.007 | 0.495 | -0.012 | 0.009 | 0.164 | 0.002 | 0.013 | 0.899 |
| rs571312 | 18 | 57839769 | 0.23 | A | C | 0.049 | 0.008 | 4.53×10^-10^ | -0.021 | 0.007 | 3.63×10^-03^ | -0.013 | 0.009 | 0.122 | -0.024 | 0.013 | 0.076 |
| rs61765651 | 1 | 72754314 | 0.83 | C | T | 0.050 | 0.008 | 2.05×10^-10^ | -0.008 | 0.008 | 0.286 | -0.003 | 0.010 | 0.747 | -0.018 | 0.015 | 0.244 |
| rs62107261 | 2 | 422144 | 0.95 | T | C | 0.121 | 0.019 | 9.55×10^-11^ | -0.006 | 0.017 | 0.745 | -0.032 | 0.021 | 0.130 | -0.013 | 0.032 | 0.681 |
| rs62500888 | 8 | 28061823 | 0.57 | A | G | 0.037 | 0.006 | 3.49×10^-10^ | -0.013 | 0.006 | 2.86×10^-02^ | -0.005 | 0.007 | 0.546 | -0.010 | 0.011 | 0.377 |
| rs7138803 | 12 | 50247468 | 0.38 | A | G | 0.073 | 0.007 | 9.18×10^-26^ | -0.004 | 0.006 | 0.560 | -0.013 | 0.008 | 0.085 | 0.005 | 0.012 | 0.681 |
| rs76227980 | 18 | 58036384 | 0.98 | C | T | 0.146 | 0.024 | 5.89×10^-10^ | -0.015 | 0.022 | 0.488 | -0.003 | 0.027 | 0.925 | -0.015 | 0.042 | 0.730 |
| rs7719067 | 5 | 153538241 | 0.42 | A | G | 0.037 | 0.006 | 3.49×10^-10^ | -0.010 | 0.006 | 0.086 | -0.003 | 0.008 | 0.652 | -0.020 | 0.011 | 0.079 |
| rs939584 | 2 | 621558 | 0.83 | T | C | 0.093 | 0.009 | 2.49×10^-25^ | -0.032 | 0.008 | 4.78×10^-05^ | -0.027 | 0.010 | 4.68×10^-03^ | -0.073 | 0.015 | 5.39×10^-07^ |
| ^#^ Only lists SNPs of the exposures after matching and harmonizing with the outcomes.  Abbreviations: BMI, body mass index; WHR, waist-to-hip ratio; WHR_adj_BMI, waist-to-hip ratio adjusted for body mass index; BC, breast cancer; ER, estrogen receptor. | | | | | | | | | | | | | | | | | |

| **Supplementary Table 2.** Estimates of total effects for obesity-related traits on the risk of BC using univariable Mendelian randomization analysis. | | | | | | | | | | | | | | | | | | | |
| --- | --- | --- | --- | --- | --- | --- | --- | --- | --- | --- | --- | --- | --- | --- | --- | --- | --- | --- | --- |
|  |  | Inverse-variance weighted | | |  | MR-Egger | | | | |  | Weighted median | | |  | MR-PRESSO | | | |
| Traits | NO.  SNP | OR (95% CI) | *P-value* | *P-_FDR_* |  | Intercept(se) | *P^#^* | OR (95% CI) | *P-value* | *P-_FDR_* |  | OR  (95%  CI) | *P-value* | *P-_FDR_* |  | NO.  SNP ^&^ | OR  (95%  CI) | *P-value* | *P-_FDR_* |
| **BC overall** |  |  |  |  |  |  |  |  |  |  |  |  |  |  |  |  |  |  |  |
| BMI | 279 | 0.89 (0.83−0.96) | 2.06×10^−3^ | 6.46×10^−3^ |  | 0.01  (0.00) | 4.54×10^-5^ | 0.60 (0.49−0.73) | 1.12×10^−6^ | 6.00×10^−5^ |  | 0.87 (0.81−0.94) | 2.18×10^−4^ | 9.81×10^−4^ |  | 270 | 0.94 (0.88−0.99) | 3.50×10^−2^ | 0.05 |
| WHR | 199 | 0.87 (0.80−0.96) | 3.77×10^−3^ | 9.70×10^−3^ |  | 0.00  (0.00) | 0.07 | 0.72 (0.58−0.90) | 4.65×10^−3^***** | 1.07×10^−2^ |  | 0.83 (0.77−0.89) | 1.83×10^−6^***** | 6.00×10^−5^ |  | 183 | 0.90 (0.84−0.96) | 9.72×10^−4^***** | 3.33×10^−3^ |
| WHR_adj_BMI | 257 | 0.94 (0.88−1.00) | 0.06 | 0.07 |  | 0.00  (0.00) | 0.16 | 0.85 (0.73−0.99) | 3.46×10^−2^ | 0.05 |  | 0.86 (0.81−0.92) | 1.16×10^−5^***** | 1.02×10^−4^ |  | 244 | 0.93 (0.88−0.98) | 3.77×10^−3^***** | 9.70×10^−3^ |
| childhood BMI | 24 | 0.78 (0.70−0.87) | 4.58×10^−6^ | 6.60×10^−5^ |  | 0.01  (0.01) | 0.49 | 0.69 (0.50−0.97) | 4.52×10^−2^ | 0.06 |  | 0.84 (0.77−0.93) | 4.98×10^−4^ | 1.89×10^−3^ |  | 22 | 0.81 (0.74−0.88) | 5.43×10^−5^***** | 3.55×10^−4^ |
| **ER+** |  |  |  |  |  |  |  |  |  |  |  |  |  |  |  |  |  |  |  |
| BMI | 279 | 0.90 (0.83−0.97) | 6.37×10^−3^***** | 1.35×10^−2^ |  | 0.01  (0.00) | 4.01×10^-3^ | 0.65 (0.52−0.82) | 2.85×10^−4^***** | 1.21×10^−3^ |  | 0.81 (0.73−0.89) | 1.00×10^−5^***** | 1.02×10^−4^ |  | 272 | 0.93 (0.87−1.00) | 0.06 | 0.07 |
| WHR | 199 | 0.88 (0.80−0.98) | 1.84×10^−2^***** | 3.31×10^−2^ |  | 0.01  (0.00) | 4.74×10^-2^ | 0.70 (0.55−0.90) | 5.79×10^−3^***** | 1.26×10^−2^ |  | 0.87 (0.79−0.96) | 3.93×10^−3^***** | 9.75×10^−3^ |  | 186 | 0.89 (0.82−0.96) | 2.30×10^−3^***** | 6.91×10^−3^ |
| WHR_adj_BMI | 256 | 0.92 (0.86−1.00) | 4.38×10^−2^ | 0.05 |  | 0.00  (0.00) | 0.16 | 0.83 (0.70−0.98) | 3.00×10^−2^ | 0.05 |  | 0.93 (0.86−1.01) | 0.08 | 0.09 |  | 249 | 0.91 (0.85−0.97) | 3.43×10^−3^***** | 9.50×10^−3^ |
| childhood BMI | 24 | 0.80 (0.72−0.89) | 4.53×10^−5^***** | 3.26×10^−4^ |  | 0.01  (0.01) | 0.29 | 0.68 (0.49−0.94) | 2.90×10^−2^ | 4.75×10^−2^ |  | 0.82 (0.74−0.90) | 9.23×10^−5^***** | 5.11×10^−4^ |  | 23 | 0.85 (0.78−0.92) | 8.42×10^−4^***** | 3.03 ×10^−3^ |
| **ER–** |  |  |  |  |  |  |  |  |  |  |  |  |  |  |  |  |  |  |  |
| BMI | 279 | 0.85 (0.76−0.95) | 4.76×10^−3^***** | 1.07×10^−2^ |  | 0.01(0.00) | 2.82×10^-4^ | 0.49 (0.36−0.67) | 1.28×10^−5^***** | 1.02×10^−4^ |  | 0.80 (0.70−0.92) | 1.77×10^−3^***** | 5.81×10^−3^ |  | 275 | 0.89 (0.81−0.99) | 3.58×10^−2^ | 0.05 |
| WHR | 199 | 0.90 (0.81−1.01) | 0.06 | 0.07 |  | 0.00(0.00) | 0.23 | 0.78 (0.59−1.02) | 0.07 | 0.08 |  | 0.93 (0.81−1.07) | 0.32 | 0.34 |  | 195 | 0.92 (0.84−1.01) | 0.09 | 0.10 |
| WHR_adj_BMI | 256 | 0.98 (0.90−1.06) | 0.62 | 0.64 |  | 0.00(0.00) | 0.50 | 0.93 (0.77−1.11) | 0.41 | 0.43 |  | 0.97 (0.86−1.09) | 0.63 | 0.64 |  | 254 | 0.96 (0.89−1.04) | 0.29 | 0.31 |
| childhood BMI | 24 | 0.71 (0.60−0.85) | 1.40×10^−4^***** | 6.82×10^−4^ |  | 0.02(0.02) | 0.34 | 0.55 (0.32−0.95) | 4.36×10^−2^ | 0.06 |  | 0.80 (0.67−0.96) | 1.39×10^−2^***** | 2.63×10^−2^ |  | 21 | 0.78 (0.68−0.91) | 4.07×10^−3^***** | 9.76×10^−3^ |
| Asterisk (^#^) denotes *P* -value for intercept (pleiotropy) in MR-Egger analysis. Asterisk (^&^) denotes Number of single nucleotide polymorphisms (SNPs) of instrumental variables in MR-PRESSO analysis.  Abbreviations: NO.SNP, the number of SNPs of instrumental variables; BMI, body mass index; WHR, waist-to-hip ratio; WHR_adj_BMI, waist-to-hip ratio adjusted for body mass index; BC, breast cancer; ER, estrogen receptor; OR, odds ratio; 95% CI, 95% confidence interval. | | | | | | | | | | | | | | | | | | | |

**Supplementary Figure 2.** Sensitive analyses of total effects for obesity-related traits on the risk of BC by excluding pleiotropic single nucleotide polymorphisms (SNPs) or excluding palindromic SNPs. Inverse-variance weighted approach was only used in the sensitive analysis. Boxes denote the point estimates of the causal effects, and error bars denote 95% confidence intervals.


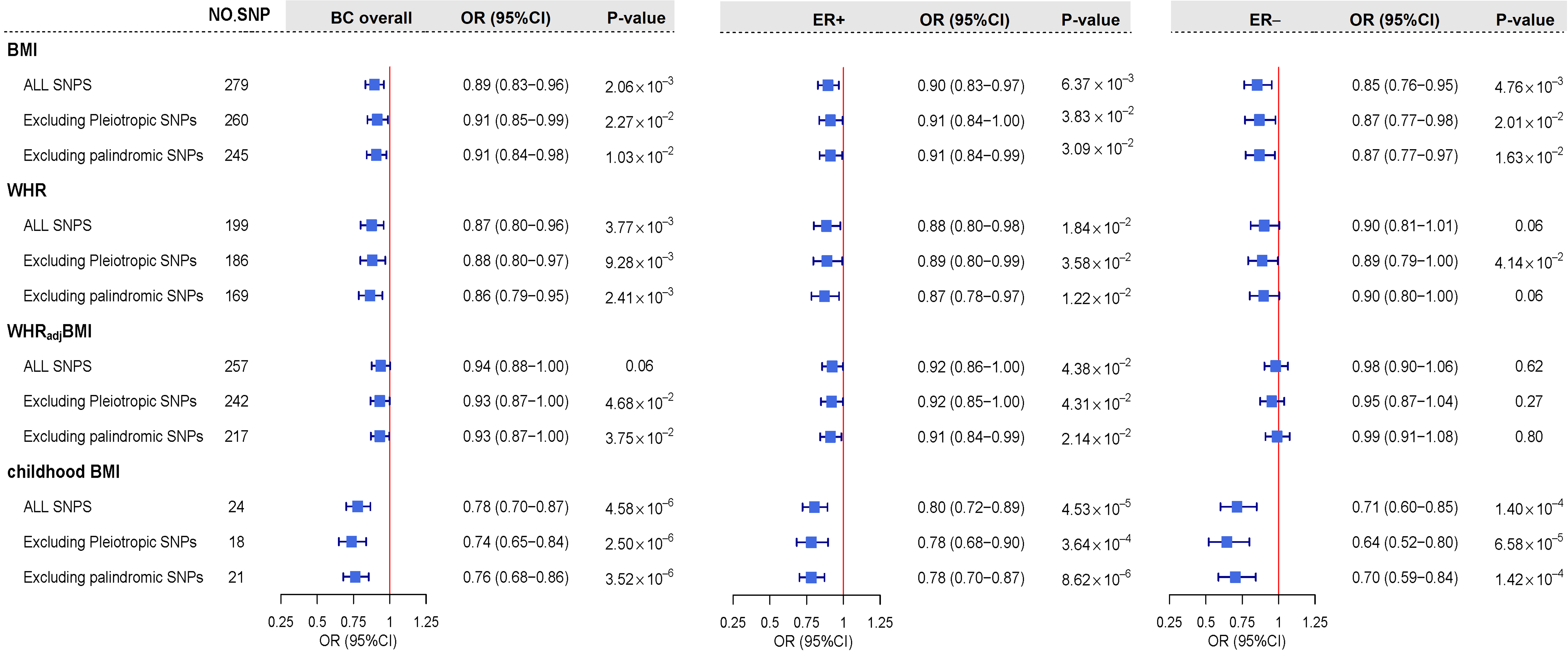


Abbreviations: BMI, body mass index; WHR, waist-to-hip ratio; WHR_adj_BMI, waist-to-hip ratio adjusted for body mass index; BC, breast cancer; ER, estrogen receptor; AAM, age at menarche; ANM, age at natural menopause; NO.SNP, the number of SNPs of instrumental variables; OR, odds ratio; 95%CI, 95% confidence interval.


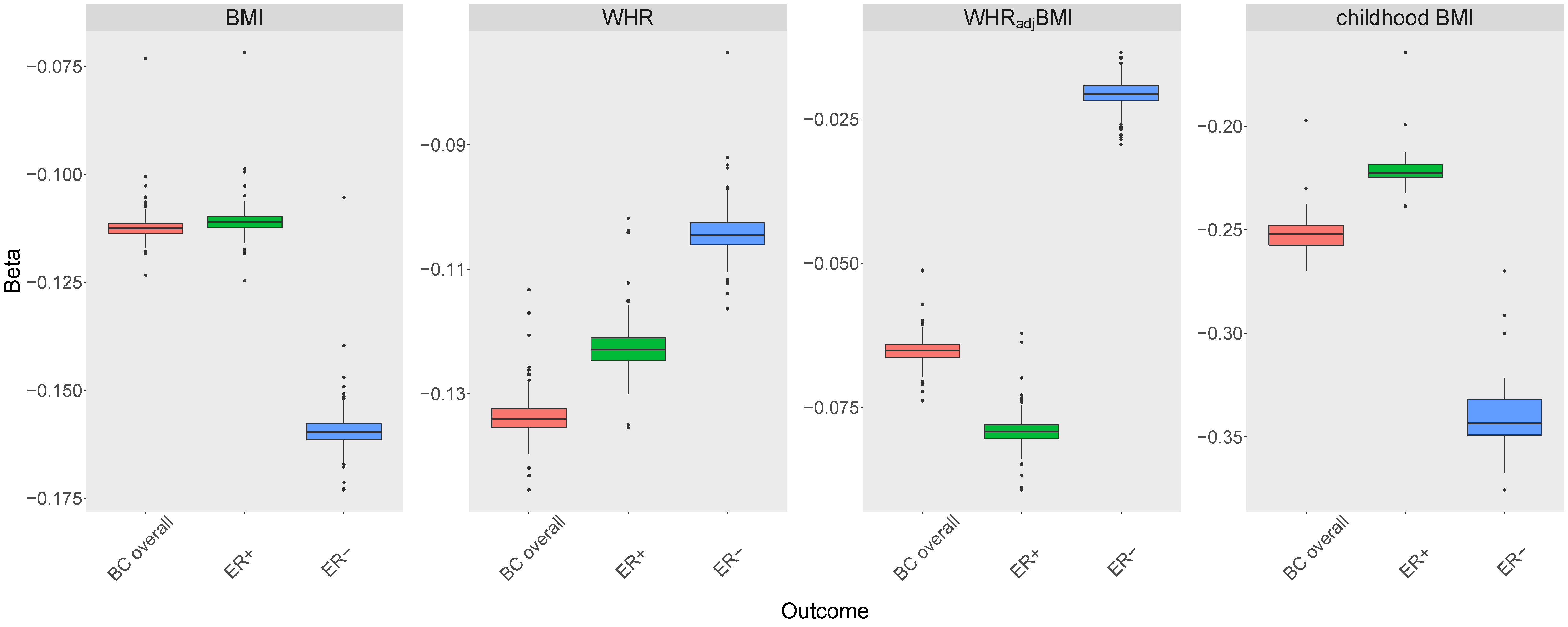


**Supplementary Figure 3.** Box plots representing results from Leave-one-out analysis which excluded each single nucleotide polymorphism (SNP) to estimate the effects of obesity-related traits on BC. The red boxes denote the effects of obesity-related traits on BC overall, the green boxes denote the effects of obesity-related traits on ER+ subtype of BC, and the blue boxes denote the effects of obesity-related traits on ER**–** subtype of BC.

Abbreviations: BMI, body mass index; WHR, waist-to-hip ratio; WHR_adj_BMI, waist-to-hip ratio adjusted for body mass index; BC, breast cancer; ER, estrogen receptor.

| **Supplementary Table 3**. Bidirectional Mendelian randomization analysis estimating the causal effects of BC overall on obesity-related traits. | | | |
| --- | --- | --- | --- |
| Traits | NO.SNP | OR (95% CI) | *P-value* |
| BMI | 149 | 1.00 (0.97-1.02) | 0.66 |
| WHR | 149 | 1.01 (0.99-1.02) | 0.52 |
| WHR_adj_BMI | 149 | 1.01 (0.99-1.02) | 0.28 |
| childhood BMI | 151 | 0.98 (0.95-1.01) | 0.13 |
| Abbreviations: NO.SNP, the number of SNPs of instrumental variables; BMI, body mass index; WHR, waist-to-hip ratio; WHRadjBMI, waist-to-hip ratio adjusted for body mass index; BC, breast cancer; ER, estrogen receptor; OR, odds ratio; 95%CI, 95% confidence interval. | | | |

| **Supplementary Table 4**. Results of genetic correlation ($r_{g}$) across each obesity trait and BC. | | | | | | | |
| --- | --- | --- | --- | --- | --- | --- | --- |
|  | BMI | WHR | WHR_adj_BMI | childhood BMI | BC overall | ER+ | ER– |
| BMI | - | 0.46  (0.03) | -0.06  (0.03) | 0.65  (0.03) | -0.03  (0.02) | -0.04  (0.02) | -0.03  (0.03) |
| WHR | 4.74×10^−68^ | - | 0.86  (0.01) | 0.21  (0.03) | 0.02  (0.02) | 0.01  (0.03) | 0.02  (0.03) |
| WHR_adj_BMI | 1.10×10^−3^ | <1×10^−200^ | - | -0.14  (0.03) | 0.04  (0.02) | 0.03  (0.03) | 0.03  (0.03) |
| childhood BMI | 1.40×10^−112^ | 7.58×10^−12^ | 4.43×10^−6^ | - | -0.06  (0.03) | -0.08  (0.03) | -0.07  (0.05) |
| BC overall | 0.10 | 0.37 | 0.08 | 4.98×10^−2^ | - | 0.99  (0.01) | 0.74  (0.03) |
| ER+ | 0.12 | 0.70 | 0.22 | 1.82×10^−2^ | <1×10^−200^ | - | 0.62  (0.03) |
| ER**–** | 0.37 | 0.50 | 0.26 | 0.20 | 2.39×10^−182^ | 1.38×10^−86^ | - |
| Upper triangle: Genetic correlations (standard error); Lower triangle: *P*-value for linkage-disequilibrium score regression (LDSC).  Abbreviations: BMI, body mass index; WHR, waist-to-hip ratio; WHRadjBMI, waist-to-hip ratio adjusted for body mass index; BC, breast cancer; ER, estrogen receptor. | | | | | | | |
